# Plasma lipidomic biomarker analysis reveals distinct lipid changes in vascular dementia

**DOI:** 10.1016/j.csbj.2020.06.001

**Published:** 2020-06-09

**Authors:** Yue Liu, Daniel K.Y. Chan, Anbupalam Thalamuthu, Wei Wen, Jiyang Jiang, Matthew Paradise, Teresa Lee, John Crawford, Matthew Wai Kin Wong, Ying Hua Xu, Anne Poljak, Russell Pickford, Perminder S. Sachdev, Nady Braidy

**Affiliations:** a Centre for Healthy Brain Ageing (CHeBA), School of Psychiatry, University of New South Wales, Sydney, Australia; b Department of Aged Care and Rehabilitation, Bankstown Hospital, Bankstown, NSW, Australia; c Neuroscience Research Australia, Randwick, Australia; d Mark Wainwright Analytical Centre, University of New South Wales, Sydney, Australia; e School of Medical Sciences, University of New South Wales, Sydney, Australia; f Neuropsychiatric Institute, Euroa Centre, Prince of Wales Hospital, Sydney, Australia

**Keywords:** Small vessel disease, Lipids, Plasma biomarkers, Vascular dementia

## Abstract

Vascular dementia (VaD) is a complex neurocognitive disorder secondary to a variety of cerebrovascular lesions. Numerous studies have shown that lipid metabolism is involved in the pathobiology of the disease. We examined the plasma lipid profiles in VaD, with the expectation of identifying reliable lipid biomarkers for VaD. 49 VaD patients and 48 healthy controls were recruited from Bankstown-Lidcombe Hospital in Sydney, Australia. Lipids were extracted by single phase 1-butanol/methanol, and untargeted analysis was performed by liquid chromatography coupled-mass spectrometry (LC–MS/MS). Univariate analysis of variance was used to examine the differences in lipid classes and individual lipids between VaD and control groups. In an independent sample of 161 subjects from the Older Australian Twins Study (OATS), elastic net penalization for the generalized linear model (Glmnet) and Random Forest were applied to the lipid levels to subcategorise the sample into vascular cognitive impairment and controls. Most lipids belonging to the classes of ceramides (Cer), cholesterol esters (ChE) and phospholipids were significantly lower in VaD plasma, while glycerides were elevated compared to controls. Levels of ChE, Cer and the two lipid classes together achieved the best accuracy in discriminating VaD from controls, with more than 80% accuracy. The probable VaD group in the OATS sample predicted by the lipid levels showed greater impairment in most cognitive domains, especially attention and processing speed and executive function from controls but did not differ in white matter hyperintensities and DTI measures. As a conclusion, plasma lipids levels, in particular Cer and ChE, are abnormal in VaD and may help discriminate them from healthy controls. Understanding the basis of these differences may provide insights into the pathobiology of VaD.

## Graphical abstract

**Figure:**
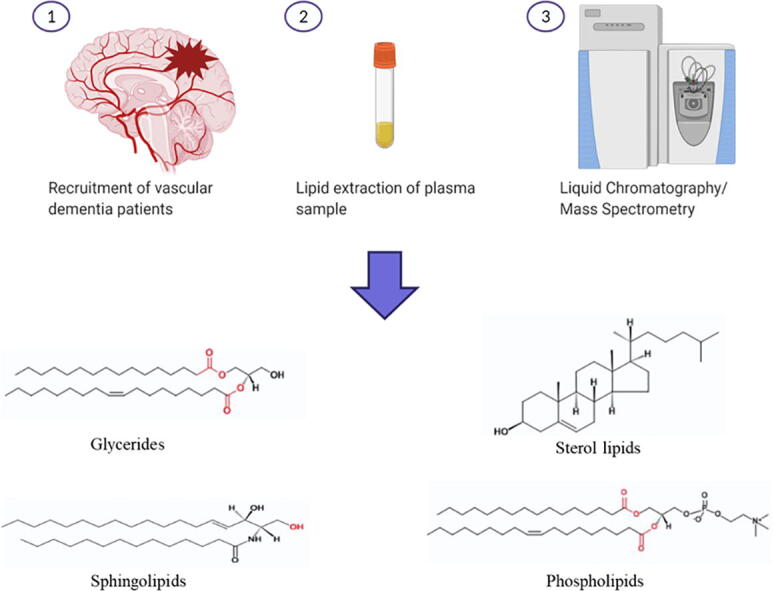

## 1 Introduction

Vascular dementia (VaD) is the second most common cause of dementia after Alzheimer’s disease (AD), accounting for 10–20% of cases. The prevalence increases with age, which is estimated to be 1.0 by the age of 71–79 years and reaching 4.1% by 80–89 years of age [1]. Many patients have a mixed diagnosis, with both vascular and Alzheimer’s pathologies being present. The pathobiology of VaD is complex, with a variety of vascular lesions likely to contribute. In the past, multiple large infarcts were considered to be the most common cause of VaD, but pathological studies from large cohorts have shown that cerebral small vessel disease (CSVD) accounts for most cases of VaD.

Cerebrovascular disease, both due to large and small vessel pathology, is best characterised by magnetic resonance imaging (MRI). Various modalities of MRI have been used to image, large and small infarcts, lacunes, white matter hyperintensities, changes in diffusivity, microbleeds and changes in blood flow, all leading to cerebral dysfunction and cognitive deficits. In the absence of MRI, a history of stroke / transient ischaemic attack or the presence of vascular risk factors such as hypertension, diabetes, hyperlipidemia, and smoking have often been used to suggest cerebrovascular pathology. Other biomarkers of VaD are however lacking.

Our focus in this study is on lipids as potential biomarkers of VaD. The rationale for this stems from the fact that VaD is frequently characterised by major changes in the white matter, a part of the brain very rich in lipids. Perturbation of brain lipid metabolism in VaD was investigated by Wallin et al. chiefly using thin-layer chromatography. They observed substantial reductions in cerebrosides and sulfatides and slight changes in some cholesterols and phospholipids in the white matter of VaD patients [2]. We argue that this disturbance should be reflected in the levels of plasma lipids. With advances in mass spectrometry methods and liquid chromatography techniques, recent lipidomics strategies have been able to detect thousands of lipid species in the blood with great sensitivity. In this study, we comprehensively profiled plasma lipids in patients with VaD and age-matched, non-demented controls using state-of-the-art liquid-chromatography coupled to mass spectrometry (LC/MS) as the principal analytical platform.

## 2 Material and methods

### 2.1 Study sample

VaD and control participants were recruited from the Memory Clinic and geriatric ward at Bankstown-Lidcombe Hospital, Bankstown, Australia. The inclusion criteria for VaD were: (1) All participants were aged >65 years; (2) Meeting the diagnosis criteria of the National Institute of Neurological Disorders and Stroke (NINCDS) and the Association Internationale pour la Recherce et l’Enseignement en Neurosciences (AIREN) [3]; (3) Clinically diagnosed by an experienced geriatrician, a psychogeriatrician and/or a neurologist, An independent geriatrician’s opinion was sought for uncertain cases and such participants would only be included if both geriatricians were in consensus of the VaD diagnosis; (4) At least one cerebral imaging modality – CT and/or MRI was needed to corroborate that the diagnosis was specifically small vessel VaD; and (5) The MMSE score was between 10 and 24 for the diagnosis of mild to moderate dementia. Exclusion criteria were: (1) Current diagnosis of malignancy; and (2) presence of life-threatening illnesses, acute psychiatric disorder; concomitant with AD component or other pathologies which were predominant aetiology of dementia.

### 2.2 Replication sample

Participants in the Older Australian Twins Study (OATS) comprised the replication sample. The detailed methodology of OATS has been described previously [4]. The OATS sample consists of twins and hence we randomly sampled one twin from each family resulting in 161 independent samples. The inclusion criteria were as follows: age ≥ 65 years, having a consenting cotwin and enough English proficiency to undertake a neuropsychological assessment. Exclusion criteria were as follows: current diagnosis of malignancy, life-threatening illness, acute psychiatric disorder, or intellectual handicap. A self-report subjective cognitive complaints questionnaire, and a comprehensive assessment using a computerized battery as well as paper and pencil tests to examine various cognitive domains including premorbid intellectual functioning, attention, memory, visuospatial function, language, executive function, information processing speed and fine motor skills. Structural MRI, including T1-weighted 3D, fluid attenuation inversion recovery (FLAIR) and diffusion tensor imaging (DTI) scans were used for the computation of neuroimaging measures such as the whole brain white matter hyperintensity (WMH) volume, average fractional anisotropy (FA), mean diffusivity (MD), axonal diffusivity (AD), radial diffusivity (RD) and peak width of skeletonized mean diffusivity (PSMD) [5].

### 2.3 Ethics approval

This VaD study has been approved by the South Western Sydney Local Health District Human Research Ethics Committee and institutional human ethics committees at each investigational site. All responses to questionnaires, and blood samples were collected in accordance with the ethical guidelines mandated by the South Western Sydney Local Health District Human Research Ethics Committee. All individuals were over 18 years of age and were approached using approved ethical guidelines. All participants will provide written consent. The OATS study has been approved by the human ethics committees of the Australian Twin Registry, University of New South Wales, University of Melbourne, Queensland Institute of Medical Research and the South Eastern Sydney & Illawarra Area Health Service.

### 2.4 Plasma collection, handling and storage

Blood was collected, and plasma was processed and stored under strict conditions to minimize pre-analytical variability [6], [7]. Briefly, fasting EDTA plasma was separated from whole blood within 1 h of venepuncture and immediately stored at −80 °C prior to bio-banking. Lipid extractions were performed within 15 min of thawing and extracts stored at −80 °C. The controls and VaD samples were mixed up and randomized for the subsequent lipid extraction and Liquid Chromatography/Mass Spectrometry.

### 2.5 Lipid extraction

We used single phase 1-butanol/methanol to extract lipids from plasma as previously described [8]**.** Briefly, 10 µL internal lipid standards (ISTDs) were added to 10 µL aliquot of each plasma sample. 100 µL of 1-butanol-Methanol (1:1 v/v) containing 5 mM ammonium formate was used to dissolve the mixture. Samples were vortexed for 10 s and then sonicated for 1 h. Afterwards, samples were centrifuged at 13,000g for 10 min. The supernatant was transferred into a fresh Eppendorf tube. A further 100 µL of 1-butanol/methanol (1:1 v/v) with 5 mM ammonium formate was added to the white pellet to re-extract any remaining lipids. The supernatant was dried in a speed vacuum centrifuge for 40–60 min. The lipids were reconstituted by adding 100 µL of 1-butanol/methanol (1:1 v/v) containing 5 mM ammonium formate to each tube. The entire contents were transferred into a 300 µL glass Chromacol vial with a glass insert prior to LC-MS.

### 2.6 Internal standards

Internal standards were purchased from *Avanti* (Alabaster, United States). The deuterated internal standards used were ceramide (Cer) d18:1/12:0, phosphatidylcholine (PC) 15:0–18:1(d7), phosphatidylethanolamine (PE) 15:0–18:1(d7), phosphatidylinositol (PI) 15:0–18:1(d7), lyso-phosphatidylcholine (LPC) 18:1(d7), cholesterol ester (ChE) 18:1(d7), diacylglycerol(DG)15:0–18:1(d7), triacylglycerol(TG) 15:0–18:1(d7)-15:0, and sphingomyelin (SM)18:1(d9).

### 2.7 Liquid Chromatography/Mass spectrometry

Lipid analysis was performed by LC ESI-MS/MS using a Thermo QExactive Plus Orbitrap mass spectrometer as previously described [8]. A Waters ACQUITY UPLC CSH™ C18 1.7 μm, 2.1 × 100 mm column was used for liquid chromatography at a flow rate of 260 gl/min, using the following gradient condition: 32% solvent B to 100% over 25 min, a return to 32% B and finally 32% B for 5 min prior to the next injection. Solvents A and B consisted of acetonitrile: MiIIiQ water (6:4 v/v) and isopropanol:acetonitrile (9: I v/v) respectively, both containing 10 mM ammonium formate and 0.1% formic acid. The first 3 min of eluent, containing the eluted salts, was diverted to waste. Product ion scan in positive and negative ion modes were performed to analyse the individual lipid species. The order of sampling was randomised prior to analysis. Examples of lipids mass spectrometry spectrum were shown in Supplementary Fig. 1.

### 2.8 Statistical methods

#### 2.8.1 Single analyte analysis

After inputting lipid MS spectrum results into Lipidsearch software version 4.2 (Thermo Fisher Scientific, Sydney, NSW AU), lipids were identified and quantified in the software according to accurate mass and fragment matching. Phospholipids, glycolipids, sphingolipids and neutral lipids were included in the database. False positives were checked manually. The LC–MS data was exported into Microsoft Excel and normalized by dividing the abundance of internal standards for each lipid class before multivariate analyses. According to the “80% rule”, peaks present in >80% samples of either group were kept for further analysis.

#### 2.8.2 Univariate analysis

The data of overall lipids group LPC, SM/Cer and LPC/PC were square root transformed and groups DG, PE, PI, SM and TG were natural log transformed to normalize their distribution. All individual lipids species were square root transformed to reduce the skewness. All lipid comparisons were adjusted for age, sex, hypertension, diabetes and status by univariate general linear model using SPSS 25. Adjusted means and mean differences of lipids were obtained from estimated marginal means. To compare all the lipid species, the mean difference (MD) was used, We used a significance threshold of 0.05 after Bonferroni correction for all comparisons (0.05/667 = 7.50*10^−5^). Partial correlation was performed to explore correlation among lipid species. Standard mean difference was utilized to measure lipids differences between hypertensive and non-hypertensive, diabetic and non-diabetic subjects in all disease group, Control group and VaD group separately. The hypertension comparison results were adjusted for age, sex and diabetes. The diabetes comparison results were corrected for age, sex and hypertension using a Univariate general linear model. The factor of disease group was adjusted as well in all group comparison.

#### 2.8.3 Classification of VaD vs control using Random Forests

We have used two machine learning algorithms, *glmnet* (elastic net penalization for the generalized linear model [glm]) and Random Forest (RF), to classify VaD vs control samples. The *glmnet* uses a combination of two penalty function with two tuning parameters to shrink the beta coefficients in the glm [9]. We have used the R language version 3.5.1 [10] package caret [11] for fitting the elastic net glm model with default options for the *glmnet* algorithm to identify the optimum values for the tuning parameters.

Random forest (RF) is an ensemble algorithm which builds several hundred rule-based decision trees and combines them into a single model. The method can be used for both continuous and categorical response variables. Random forest is a very popular choice among many machine learning algorithms as they achieve very high accuracy and provide good features like variable of importance, missing value imputation and outlier detection with less number of tuning parameters. The RF model was also implemented using the care package. The tuning parameter “mtry”, the random number of predictors at each split, used by the caret package was found to be 2 in most our analysis. The default number of 500 trees decision trees were used for our analysis.

All the variables were centered and scaled in the pre-processing step. The data was randomly split into 70% training and 30% tests samples maintaining the proportion of cases and controls in the training and test samples as in full data. For the training data, the algorithms were run with 3 cross validation with 5 repeats. To avoid bias due to single random split of the original data, we have repeated the analysis 10 times and the results were summarized over the 10 iterations.

The classification accuracy of the glmnet and RF models were examined based on several subgroups of the lipid species. The Receiver Operating Curve (ROC) and area under curve (AUC) were obtained using the R package pROC [12]. Average sensitivity (proportion of VaD cases predicted by the model in the test data), specificity (proportion of controls predicted by the model in the test data) and the AUC across the 10 iterations are reported. The RF algorithm returns a measure of variable of importance, which the estimated increase in the error rate (mean decrease in Gini index) if the data for the variable is permuted in the dataset. The predictors were rank ordered using the variable of importance and averaged across the 10 iterations. Similarly, beta-coefficients from the optimum glmnet model were averaged over 10 iterations.

#### 2.8.4 Classification of VaD vs control using average beta-coefficients:

The probability of VaD given the data for the lipids, P (VaD | x) can be obtained based on the average beta-coefficients.

Assuming the logistic regression model for VaD vs Controls,

$$P\left(Y_{i}|x\right)=\frac{\exp(b_{o}+xB)}{1+exp(b_{o}+xB)}$$

where *Y_i_* = 1 for VaD and Y_i_ = 0 for controls, b_0_ is the intercept and ***x*** is a row vector of scaled lipid levels and *B* is the column vector of estimated beta coefficients.

#### 2.8.5 Phenotype variance explained based on average beta-coefficients

Given the vector y = (y_1_,y_2_,…,y_n_) of case control status (y_i_ = 1 for VaD and y_i_ = 0 for control), the log likelihood function given the lipid profile *x* can be calculated as

$$LL1=\sum_{i=1}^{n}{\{y}_{i}log(P\left(y_{i}|x\right)+\left(1-y_{i}\right)\left(1-log\left(P\left(y_{i}|x\right)\right)\right\}$$

where $P\left(y_{i}|x\right)$ is the probability of VaD, which can be calculated using the formula given above. Similarly,the log likelihood under the null model (*LL0*) without any covariate can obtained by putting ***x = 0.***

In addition to the ROC analysis, we have examined the variance explained by the average model derived using the glmnet algorithm. The pseudo R-square for the logistic regression using the Nagelkerke formula [13] is

$$R_{\mathrm{Nag}}^{2}=\frac{\left\{1-\exp\left(-\frac{2\left(LL1-LL0\right)}{n}\right)\right\}}{\left\{1-\exp\left(LL0\frac{2}{n}\right)\right\}}$$

The coefficient of Discrimination given by Tjur [14] is

$$R_{\mathrm{Tjur}}^{2}=\frac{\left\{\sum_{y_{i}=1}P\left(y_{i}=1|x\right)\right\}}{n1}-\frac{\left\{\sum_{y_{i}=0}P\left(y_{i}=0|x\right)\right\}}{n0}$$

where n1 and n2 are the number of cases and controls in the sample. The above formula of coefficient of determination is just the difference between average probabilities of cases and controls.

## 3 Results

### 3.1 Patient demographics

This study recruited 48 VaD patients and 49 age and sex matched cognitively normal controls from Bankstown-Lidcombe hospital and the Sydney community. The demographics of all participants are summarized in Table 1. As expected, multiple cognitive domains detected by MMSE including orientation, immediate memory, memory recall, attention and visuospatial were significantly lower in VaD subjects. Healthy controls had higher frequency of subjects with secondary school or higher education (χ^2^ = 4.461, p = 0.035). The VaD and normal controls did not differ in vascular risk factors, including frequency of hypertension, diabetes and current smokers. There were no significant difference of HDL and LDL and lipid medication between 2 groups. VaD patients presented significantly lower BMI. However, only 18/48 vascular dementia patients have height and weight information, which induced a large part of missing BMI data in VaD group. We did not collect enough data of LDL and HDL, with 5 controls and 6 VaD patients. The current data showed no difference of HDL and LDL between 2 groups. Dietary habits including consumption of fruits, vegetables, red meat, fish, beans, curry and eggs were not differed between control and VaD groups.

**Table 1:** Characteristics of VaD patients.

	Healthy Control (49)	Vascular Dementia (48)	Statistics	P value
**Age**	83.41(4.33)	82.47(5.79)	t = 0.840	p = 0.403
**Sex** (*Male percentage*)	53.10%	52.10%	χ2 = 0.009	p = 0.923
**BMI**	26.69(3.8)	24.29(3.81)	t = 2.085	p = 0.041
**Education**				
*Primary school*	18.40%	39.10%	χ2 = 6.044	p = 0.049
*Secondary school*	65.30%	54.30%		
*Tertiary school or higher*	16.30%	6.50%		
**Diabetes**	12.20%	25%	χ2 = 2.610	p = 0.106
**Hypertension**	55.10%	64.60%	χ2 = 3.590	p = 0.166
**Current smokers**	4.10%	2.10%	χ2 = 0.323	p = 0.570
**Current drinkers**	63.27%	45.83%	χ2 = 2.973	p = 0.085
**Dietary^a^**				
Fruits	4.59(0.91)	4.25(1.31)	t = 1.492	p = 0.139
Vegetables	4.65(0.69)	4.35(1.10)	t = 1.603	p = 0.112
Red meat	3.27(1.13)	3.44(1.03)	t = −0.783	p = 0.435
Fish	2.8(0.87)	2.44(0.99)	t = 1.902	p = 0.060
Beans	3.04(1.47)	3.02(1.06)	t = 0.077	p = 0.939
Curry	1.08(1.19)	1.38(1.00)	t = −1.313	p = 0.192
Eggs	2.17(1.27)	2.54(1.03)	t = −1.564	p = 0.121
**HDL^b^**	1.16(0.30)	1.22(0.29)	t = −0.332	p = 0.259
**LDL^b^**	3.02(1.13)	2.28(0.91)	t = 1.204	p = 0.765
**Lipid lowering drugs**	40.82%	31.25%	χ2 = 0.962	p = 0.327
**MMSE**				
*Orientation*	9.98(0.14)	6.68(2.67)	t = 8.55	p < 0.001
*Registration*	3.00(0.00)	3.10(1.00)	t = −0.68	P = 0.498
*Attention*	4.75(0.76)	3.29(1.89)	t = 4.91	p < 0.001
*Memory recall*	2.54(0.62)	1.73(1.58)	t = 3.27	P = 0.002
*Language*	8.04(0.46)	7.22(1.27)	t = 4.17	p < 0.001
*Visospatial*	0.92(0.28)	0.51(0.51)	t = 4.76	p < 0.001

a. Dietary scores: 0 = Never, 1 = Barely, 2 = occasionally (<1 time/week), 3 = occasionally (1–3 times/week), 4 = occasionally (4–6 times/week), 5 = almost every day year around. b. The number of subjects having HDL and LDL data: control 5, vascular dementia 6.

### 3.2 Differences in group lipids between control and vascular dementia patients

A total of 667 distinct lipid species from 9 lipid subclasses including neutral lipids such as ChE, glycerolipids DG, TG; sphingolipids including Cers and SMs; and phospholipid subclasses including PC, PE, and PI were analyzed adjusted for age, sex, hypertension and diabetes(Table 2). Ceramides were observed to be significantly lower in VaD. There was no significant difference in total SM lipids. However, the ratio of SM/Cer was upregulated in VaD. Most phospholipids presented significant changes in VaD plasma. PC and PE was both decreased in VaD plasma. No significance was found in PI lipids. The lyso-phospholipids, LPC showed significantly reduced levels in VaD. The ratio of LPC/PC augmented significantly in VaD subjects. Cholesterol esters (ChE) were significantly lower and glycerides increased in VaD. Most of results did not change when additionally corrected for current drinking status, dietary habits and lipid lowering drugs, except that LPC and PC were no longer significantly different between 2 groups.

**Table 2:** Mean value of transformed group lipids in VaD and Control group.

Lipid groups	FC	Control^a^		VaD^a^		MD^a^	pvalue^a^	Control^b^		VaD^b^		MD^b^	pvalue^b^
	(VaD/ Control)	Mean	SE	Mean	SE	(VaD-Control)		Mean	SE	Mean	SE	(VaD-Control)	
GroupCer	0.610	5.733	0.243	3.468	0.246	−2.265	<0.001	5.738	0.264	3.495	0.261	−2.244	<0.001
GroupSM	0.764	9.113	0.073	9.141	0.074	0.028	0.793	9.117	0.081	9.146	0.08	0.029	0.811
SM/Cer	1.161	42.359	1.682	54.113	1.699	11.754	<0.001	42.504	1.815	54.13	1.793	11.627	<0.001
GroupChE	0.814	755.285	27.686	596.979	27.978	−158.307	<0.001	758.276	31.234	597.749	30.872	−160.527	0.001
GroupDG	1.282	6.012	0.064	6.277	0.064	0.265	0.005	5.993	0.067	6.285	0.066	0.293	0.004
GroupTG	1.319	4.464	0.043	4.695	0.043	0.231	<0.001	4.471	0.045	4.686	0.045	0.215	0.002
GroupLPC	0.756	4.799	0.185	4.233	0.187	−0.566	0.036	4.781	0.193	4.28	0.191	−0.501	0.083
GroupPC	0.838	229.266	10.427	192.618	10.537	−36.648	0.016	226.11	11.288	195.891	11.157	−30.219	0.074
LPC/PC	0.947	0.284	0.013	0.339	0.013	0.055	0.004	0.285	0.014	0.338	0.014	0.053	0.013
GroupPE	0.763	2.544	0.052	2.329	0.053	−0.215	0.005	2.524	0.057	2.349	0.056	−0.176	0.04
GroupPI	0.926	3.816	0.035	3.731	0.035	−0.085	0.089	3.821	0.036	3.723	0.036	−0.097	0.073

a. Mean value of transformed group lipids (adjusted for age, sex, diabetes, hypertension); b. Mean value of transformed group lipids (adjusted for age, sex, diabetes, hypertension, current drinkers, dietary and lipid-lowering drugs).

FC: fold change of original lipid abundance in VaD/control. MD: adjusted mean difference.

Cer: ceramide; PC:phosphatidylcholines; PE: phosphatidylethanolamines; PI: phosphatidylinositol (PI); LPC: lyso-phatidylcholines; ChE: cholesterol esters, DG: diacylglycerol(DG); TG: triacylglycerols; SM: sphingomyelin.

#### 3.2.1 Ceramides and sphingolipids

The level of various Cer species were lower in plasma of VaD patients (Supplementary Tables 1 and 2). Molecular profiles of the sphingolipidome showed that Cer species comprising short-chain fatty acyls (16-18C) and long chain fatty acids were both lower in VaD. Only some Cers comprising 16 C tended to be slightly higher in VaD. SM species did not significantly differ between VaD patients and controls after Bonferroni correction. In contrast to high variance in changes of dihydroxy SM lipids between VaD patients and controls, most trihydroxy SM in general showed a tendency to be higher in VaD patients. The significance of these lipid differences did not change after adjusting for lipid lowering drugs

#### 3.2.2 Phospholipids

VaD patients had higher levels of plasma LPC (Supplementary Tables 1 and 2). LPC (22:6) showed a significantly higher level in VaD. Major phospholipids classes including PC, PE and PI showed a tendency to be lower in VaD patients compared to healthy controls. PC lipids containing highly unsaturated fatty acid chains were frequently affected. This included PC(22:6/13:0), PC(36:6), PC(37:6), PC(38:3), PC(38:5), PC(38:6), PC(38:8), PC(39:3), PC(40:7), PC(40:8), PC(40:9) , PC(42:7), PC(42:9), and PC(42:10). The slightly unsaturated PC lipids comprising PC (37:1) and PC (38:1) were significantly lower in VaD. With regards to PE lipids, all significant differences were observed in lipids containing fatty acid chain of 22:6, and included PE(16:0/22:6)(MD = −0.887, p = 6.16E−5),PE(18:0/22:6)(MD = −1.003, p = 7.50E−6), PE(18:0p/22:6) (MD = −0.990, p = 9.50E−6), PE(18:1p/22:6) (MD = −1.032, p = 4.34E−6) and PE(20:0p/22:6) (SMD = −0.928, p = 2.98E−5). Consistent with PE, only 1 PI containing the fatty acid chain (22:6), PI (18:0/22:6) (MD = −1.294, p = 2.20E−8) was significantly lower in VaD patients.

#### 3.2.3 Cholesterol esters and glycerolipids

Most ChEs showed lower abundance in VaD patients (Supplementary Tables 1 and 2). Significant group differences were observed for ChE (16:0), ChE (17:0), ChE (18:1), ChE (18:1), ChE (20:3), ChE (20:4) and ChE (20:5). DGs were mainly higher in VaD patients compared to healthy controls. Only DG (12:0/20:5) (MD = 0.951, p = 1.95E−05) and DG (18:0/18:0) (MD = 1.199, p = 1.59E−07) showed significance. 49 triglycerides showed significant differences between VaD patients compared to healthy controls. TGs showed significantly higher abundance in VaD. However, other TGs, and particularly those containing the fatty acid chain 15:0 was lower in plasma from VaD patients.

### 3.3 Partial correlations between significantly different lipids

After adjustment for age, sex, hypertension and diabetes, we found high correlation among Cers, and monohydroxy Cers in particular (Supplementary Fig. 2). In addition, ChEs, PE lipids and certain PCs were observed to be correlated with Cers although the correlations were weaker for monohydroxy Cers. LPC (22:6) showed low correlation with almost all other lipid species. ChEs and PCs showed weak association with each lipid groups separately. Phospholipids did not present high association with ChE, except for ChE (20:5).

### 3.4 Analysis of lipid differences between hypertension and type 2 diabetes

**Fig. 1:**
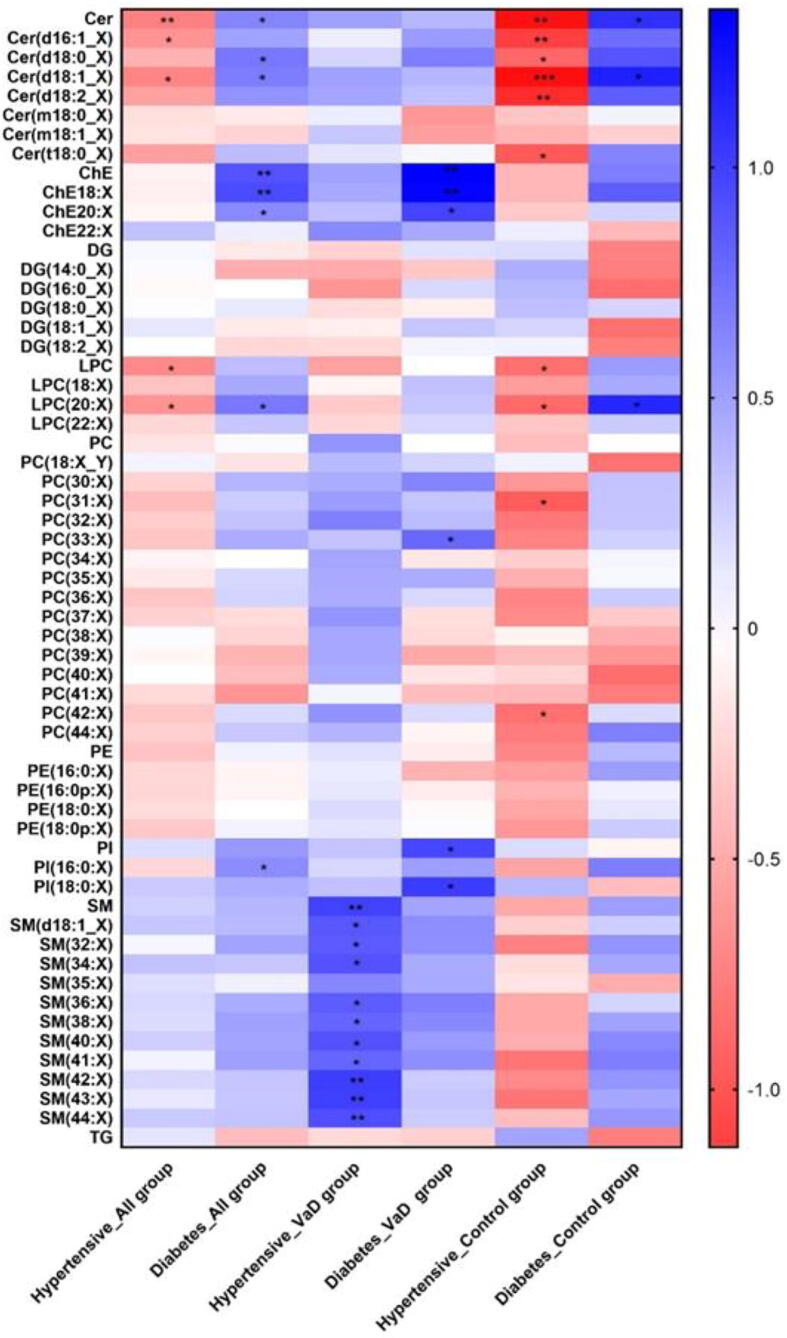
Standard mean difference of lipid classes. (* p < 0.05, ** p < 0.01, *** p < 0.001 **** p < 0.0001, adjusted for age, sex, diabetes, hypertension, current smoking and BMI).

Stratified analysis was performed to find additional clinical significance by combinatorial effects between lipid groups and environmental risk factors such as hypertension and type 2 diabetes Fig. 1. We observed that in the disease and control group, the hypertension group showed significantly lower plasma cers while diabetes showed an opposite effect. There was no significant difference in cers in VaD with or without hypertension or diabetes. However, all VaD patients showed a higher trend of cer under the influence of these 2 risk factors. In contrast to Cer, SMs were increased in VaD patients with a high blood pressure. Almost all SMs increased in the presence of hypertension.

ChE were only elevated in VaD patients with diabetes and in all groups, and no significant difference was observed in hypertensive subjects. The glycerolipids, DGs and TGs did not change significantly in the presence of hypertension or diabetes suggesting that pathways involved in DG and TG metabolism are not affected by these risk factors.

As well, some phospholipid species differed significantly in the presence of hypertension or diabetes. LPCs were affected in all group or Control group. Plasma group LPC lipid levels were higher in hypertensive patients although decreases in the subgroup LPC (20:X) was observed in diabetic patients. Plasma levels of PC (30:X) and PC (44:X) decreased in hypertensive cognitively normal subjects but PC (33:X) was upregulated in diabetic VaD patients. VaD patients with diabetes also showed higher group PIs and PI (18:0X) compared to patients without diabetes.

### 3.5 Classification of VaD vs control

**Fig. 2:**
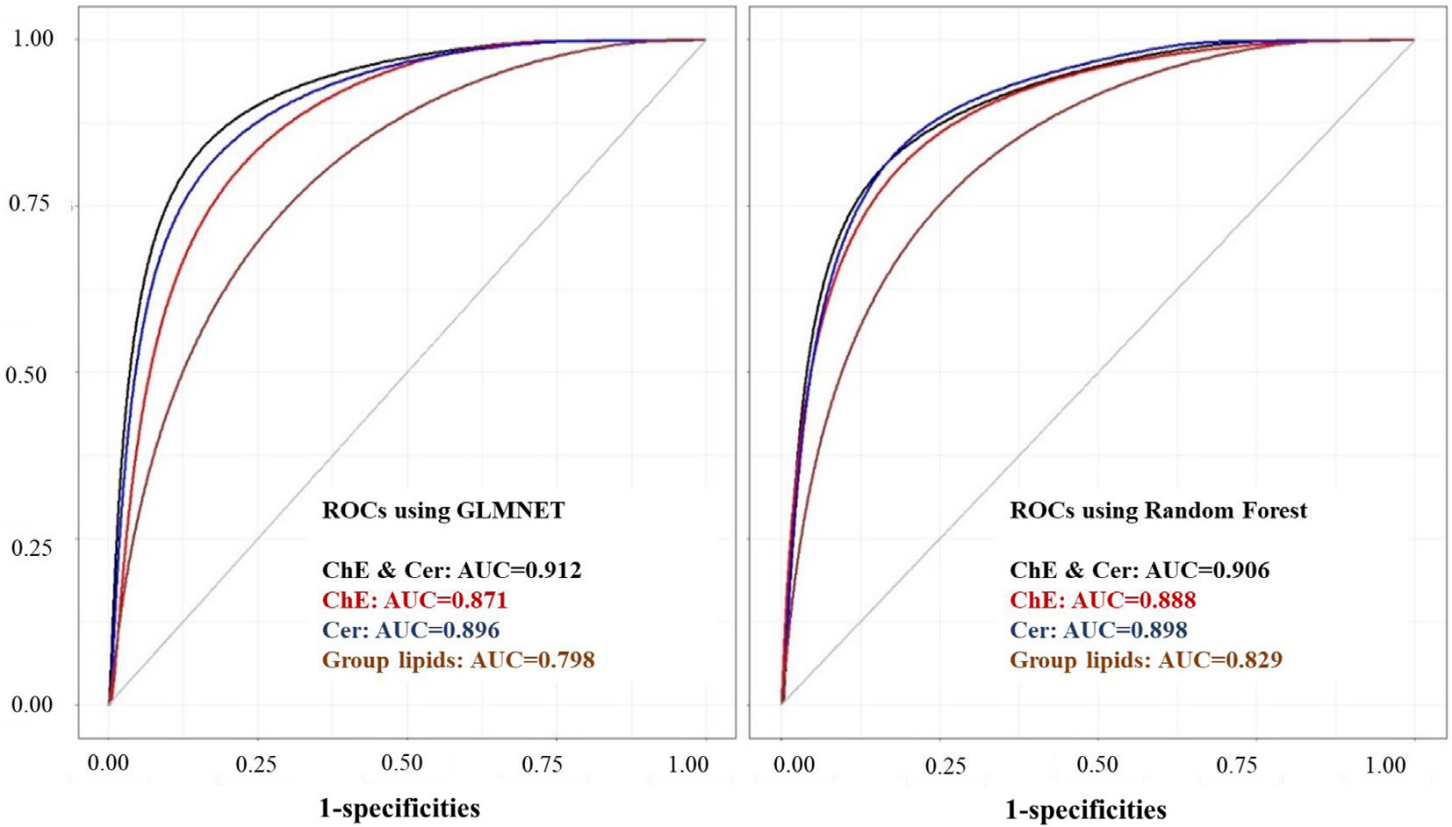
ROC of ChE, Cer and group lipids by GLMnet and Random Forest.

We considered several classes of lipids for classification of VaD vs controls. The glmnet net and Random forest algorithms with 70% training and 30% test was used in all the analyses. The analyses were repeated 10 times and results were summarized across the 10 iterations. The algorithms were run in turn using the full list of individual lipids, all the 9 sub classes of lipids, group lipids and combined ChE and ceramides. The average sensitivity, specificity and AUC of the test data are summarized in Table 3. The ROC curves for top 3 groups, ChE, Cer and group lipids (AUC > 0.85 in RF) and the combined ChE and Cer variables are presented in Fig. 2.

**Table 3:** Random forest and GLM results of group lipids.

Lipid Class	RF Sensitivity	RF Specificity	RF AUC	GLM Sensitivity	GLM Specificity	GLM AUC	AUC diff	R^2^ Nag	R^2^ Tjur
All Lipids	0.69	0.82	0.83	0.78	0.67	0.80	0.03	0.58	0.42
Cer	0.87	0.83	0.90	0.85	0.77	0.90	<0.01	0.79	0.59
ChE	0.82	0.80	0.89	0.80	0.81	0.87	0.02	0.77	0.61
ChE & Cer	0.82	0.85	0.91	0.85	0.87	0.91	−0.01	0.91	0.79
DG	0.76	0.77	0.81	0.70	0.75	0.78	0.03	0.52	0.35
LPC	0.71	0.75	0.78	0.70	0.63	0.71	0.07	0.63	0.44
PC	0.74	0.78	0.81	0.68	0.77	0.80	0.01	0.74	0.52
PE	0.74	0.74	0.80	0.73	0.74	0.79	0.01	0.56	0.38
PI	0.78	0.78	0.83	0.75	0.74	0.82	0.01	0.64	0.48
SM	0.62	0.68	0.71	0.72	0.62	0.73	−0.02	0.69	0.50
TG	0.85	0.77	0.81	0.82	0.77	0.83	−0.01	0.65	0.45

R^2^Nag and R^2^Tjur are the variances explained by the lipids in the lipid classes using the Naglkerke and Tjur methods. Abbreviations: RF – Random Forrest; GLM – glmnet.

The RF algorithm performed slightly better than the glmnet for most of the VaD vs controls analyses. However, in all the measures of classification accuracy, the magnitude of difference between these two algorithms was very small. In the case of group lipids, RF gave better specificity and reduced sensitivity.

In general, the lipid subclasses LPC and SM had less classification accuracy compared to the other subclasses. The top 3 subgroups (ChE & Cer, ChE, Cer) resulted in good classification accuracy with more than 85% AUC. The classification model using all the lipid species (667 lipids in total) did not give improved prediction accuracy compared to the other groups of lipids and hence the results for the full list of lipids are not summarized here.

The lipids in the subgroups were ranked based on the variable of importance and the average rank across the 10 iterations are presented in the supplementary table 3. The average beta coefficients from the optimal glmnet model is also included in the Supplementary Table 3. In general, there was good consistency between the variables of importance of the RF model and the magnitude of the beta-coefficients in the glmnet.

The average beta-coefficients of lipid classes were used in logistic regression to derive the predicted probabilities. The model based on the average beta-coefficients explained a large proportion the phenotypic variance as captured by the two measures of R-square. Consistent with the AUC, higher variance was explained by combined lipid classes ChE & Cer. There was a high correlation (0.98) between two measures of R-square. Though the measure R^2^ -Tjur was always less, it had a slightly better correlation with the AUC measures compared to the Naglkerke’s R^2^ measure.

### 3.6 Validation of a plasma lipid-based classification into ‘vascular cognitive impairment’ and normal in the independent OATS sample

We examined the validity of the lipid-based classification in the independent OATS cohort which comprises non-demented community dwelling twins aged 65 and years. One twin from each pair was randomly selected resulting in 161 independent samples. We restricted the validation analysis to the top performing lipid group (Cer & ChE). The lipid measures were used to classify the OATS into putative ‘vascular’ and ‘normal’ groups. The two groups were then compared using a comprehensive battery of cognitive tests and neuroimaging data. We examined the profile of the predicted vascular cognitive impairment and controls from wave 3 on several cognitive measures, whole brain white matter hyper intensities (WMH), average DTI measures FA, MD, RD, AD, and PSMD.

The *t*-test comparing the mean difference by using z scores between the putative ‘vascular’ and normals within the validation OATS subsample (n = 161) is given in the Table 4. The vascular group (n = 46) had poorer performance in all cognitive domains compared to the ‘normal’ group (n = 115), especially in attention and processing speed (t = 2.433, p = 0.018) and executive function (t = 2.908, p = 0.005). There is no significant difference between the two groups in WMHs and the standard DTI measures. However, the peak width of skeletonized mean diffusivity (PSMD) was lower in the ‘vascular’ group (t = 2.783, p = 0.008).

**Table 4:** Difference of Cognitive function, whole brain white matter hyperintensities (WMHs) and DTI measures between predicted VaD and predicted control samples in OATS.

Variable	Mean Controls	Mean Predicted Vascular Cognitive Impairment (VCI)	t-value	p-value
Global Cognition	0.114	−0.375	2.922	0.005
Attention Processing Speed	0.099	−0.326	2.433	0.018
Memory	0.087	−0.288	2.296	0.024
Verbal Memory	0.079	−0.260	2.097	0.039
Language	0.083	−0.270	1.919	0.059
Visuo Spatial Memory	0.056	−0.185	1.461	0.148
Executive Function	0.117	−0.376	2.908	0.005
wholeBrainWMHvol_mm3	−0.004	0.013	−0.095	0.925
FA*	0.039	−0.130	0.795	0.431
MD*	0.029	−0.096	0.641	0.524
AD*	0.019	−0.053	0.351	0.727
RD*	0.025	−0.085	0.544	0.589
PSMD*	0.127	−0.425	2.783	0.008

Abbreviations: FA - fractional anisotropy; MD – mean diffusivity; AD – axial diffusivity; RD – radial diffusivity; PSMD - peak width of skeletonized mean diffusivity. * whole brain average.

## 4 Discussion

To our knowledge, this is the first and largest comprehensive nontargeted plasma lipidomics study in VaD to date. We identified a combination of lipids that classified VaD patients with high accuracy (>80%) compared to control subjects. While our lipidomics signature is unlikely to be useful for a diagnostic purpose yet, it highlights several important biological processes that may be associated with the pathogenesis of VaD.

### 4.1 Identification of the roles of lipids associated with VaD

Sphingolipids belong to a class of lipids composed of a sphingoid base backbone that is modified to form Cer and other important lipids, such as SM and glycosphingolipids. Sphingolipids are essential for the maintenance of membrane structure stabilization, cell-to-cell recognition and secondary messenger signalling [15]. Altered sphingolipid metabolism has been associated with the pathogenesis of several neurodegenerative diseases such as AD, various cancers and the metabolic syndrome [16], [17], [18], [19]. Contrary to a previous study [20] that reported an increase in SM in the white matter of VaD patients, we did not observe significant changes in SM between controls and VaD. However, the SM/Cer ratio was significantly higher in VaD, which may indicate an increase in Cer channelled into SM in the plasma of VaD patients. The increase in serum SM level has been shown to be a major risk factor for cardiovascular diseases [21], and is strongly correlated with parameters of insulin resistance, and lipid metabolism, which are major risk factors for dementia [22]. The synthesis of SM from SM synthase 2 has been associated with increased vascular risk in animals exposed to a high fat diet [23]. Our data also suggest that trihydroxy SM are more associated with VaD than other SM species, although further research is needed to validate these findings, especially during the earlier stages of the disease.

Ceramides are a family of lipids that consist of sphingosine covalently linked to a fatty acid and are densely located in the cell membrane. Our study observed significantly lower plasma levels of Cers. Consistent with this, a recent lipidomics study of post mortem brain tissues of VaD subjects reported an overall decrease in sphingolipids, including decreases in very long chain Cers [20]. Cer deficiency has been shown to be related to white matter demyelination in MS patients [24]. Severe white matter changes leading to demyelination and axonal loss play a role in the broad functional brain changes underlying vascular cognitive impairment and in the associated cerebral atrophy. Decreased Cer was also found to be related to mitochondrial dysfunction in genetic neurological diseases [25]. Furthermore, the ablation of Cer synthase 2 can induce chronic oxidative stress by disrupting mitochondrial respiratory chain function [26].

Phospholipids, including PC and PE, were downregulated in VaD plasma. Polyunsaturated PC lipids were specifically lower compared with healthy controls, supporting a possible neuroprotective role of highly unsaturated fatty acid chain in phospholipids. Accumulating evidence has found a link between PC levels and cognitive function, with low plasma PC levels being highly predictive of cognitive decline and age-related membrane degeneration [27]. PC may provide a significant supply of choline and maintains structural integrity of the neuronal membrane [27]. PC has been positively associated with catalytic activity of gamma-secretase enzyme by regulating cell membrane thickness and the lipid microenvironment [28]. We found an overall decrease in PC levels in the plasma of VaD subjects. A decline in PCs and LPCs has been previously reported in blood and postmortem brain samples in several models for neurological and cardiovascular diseases [29], [30]. All these metabolites were measured using the same technological platform in our sample set. This supports the importance of PCs in VaD pathophysiology, and many VaD patients have been shown to have associated AD pathology.

Decreased levels of PCs may be associated with irregular phospholipase A_2_ (PLA_2_) activity [31]. PLA_2_ are enzymes that are involved in the production of free fatty acids and LPCs. It has been demonstrated that Aβ_42_ peptide increases PLA_2_ activity [32]. We also found lower levels of LPCs in VaD patients compared to non-demented controls. LPCs are formed by a PLA_2_ catalysed reaction and are acylated with acetyl-CoA to regulate normal neural membrane composition. Impaired PLA_2_ activity may be due to altered levels of LPC and an increase in the LPC/PC ratio [33].Taken together, these studies provide support for impaired anabolism of phospholipids in various biological specimens in accordance with increased phospholipid catabolism due to overactivation of phospholipase A_2_ (PLA2) in a clinical setting. Increased LPC production from PC is regulated by cytosolic PLA2G4A in Land’s cycle and the secretory soluble PLA2G2A leading to abnormal intracellular Ca2+ signalling via the G-protein coupled receptor GPR132, G2A [34]. Increased intracellular Ca2+ can promote the activity of CAMKII and together with Cer-PP2A-GSK3β, can induce tau hyperphosphorylation and the formation of NFTs. The insignificant difference of LPC and PC after controlling for alcohol and dietary habits might indicate the important effect of changed food consumption caused by dementia status on plasma LPC and PC. However, the ration of LPC/PC were still significantly higher in plasma of VaD subjects, showing important role of increased production of LPC from PC in VaD pathobiology.

PE is the key regulator of cell membrane fluidity [35], which is important for cell trafficking, including cell binding to insulin. In addition, PE influences a variety of cellular processes and the stability and function of numerous membrane proteins. Mice lacking either of the two major PE-producing pathways are vulnerable to chronic lipid-induced ER stress [36]. Since a multitude of neuropathological processes can lead to a decrease in PC and PE, it is likely that reduced levels of these phospholipids are linked to neuronal death. Moreover, there are some risk factors in VaD patients that may act independently of large and small vessel disease. Increases in the activities of these enzymes is likely to represent a compensatory mechanism in response to low PE/PC. It has been suggested that the nutritional precursors, uridine, docosahexaenoic acid (DHA), and choline, can increase the levels of PC and PE, and other lipids including SM and PI in the brain, to promote synaptogenesis and neurite outgrowth which is damaged by amyloid plaques. Increasing PC increases SM levels since PC is one of two substrates for SM biosynthesis. All the significantly altered PE lipids contained C22:6 chains. Consistently, Aid, S., et al. previously demonstrated that a dietary-induced depletion of DHA (C22:6), which is a precursor for phospholipids, increased the spontaneous release of acetylcholine (ACh) in the rat hippocampus and reduced its potassium chloride evoked–release [37]. This treatment is likely to promotes dendritic spine and synapse formation.

PI is another phospholipid that is involved in mediating Ca^2+^ mobilization in response to many hormones, neurotransmitters, growth factors [38]. While PI remained unchanged overall, we observed a significant reduction in PIs comprising fatty acid chain C22:6 (PI18.0/22.6) in VaD. This is the first study to identify distinct changes in the sn-1 and sn-2 fatty acyl chains. Alterations in the fatty acid composition of PI and acyl chain remodelling may affect neuronal function and play a causal role in VaD. Further studies are necessary to elucidate the role of reduced PI18.0/22.6 in VaD. Interestingly, uridine can be converted to CTP in the brain which leads to a subsequent increase in the level of CDP-DG, which is utilised as a substrate for PI synthesis [39].

Most ChEs were found to be lower in the plasma of VaD subjects compared to controls. ChE are produced in the plasma by the conversion of fatty acids to cholesterol from PC by the enzymatic activity of cholesterol acyl transferase (LCAT) [40]. While free cholesterol can be taken up by APOE containing liposomes (e.g. HDL) and is bound to the outer particle surface, esterification enhances cholesterol uptake within the interior of the lipoproteins and enhances cholesterol transport through the blood stream. LCAT has a preference for highly unsaturated fatty acid chain PC and can link the reduction in PC to dysregulation of specific steps in cholesterol metabolism in VaD [41]. Another enzyme, acyl-coenzyme A can also esterify cholesterol in other tissues. About one-third of ChE are transferred from HDL to APOB- containing lipoproteins e.g. VLDL. This process leads to lower plasma HDL levels and reduces HDL size as observed in type 2 diabetes, and reduced ChE transfer protein leads to increased HDL ad lower LDL, which is phenotypically antiatherogenic [42].

We also found significantly higher levels of DG (12:0/20:5) and DG (18:0/18:0) in the plasma of VaD subjects. DGs are important in maintaining structural integrity and signal transduction [43]. The levels of DGs are regulated by four major metabolic pathways: i) conversion toglycerophospholipids; ii) phosphorylation via DG kinase to generate phosphatidic acids; iii) hydrolysis by DG lipase, lipoprotein lipase (LPL), and hormone-sensitive lipase to generate MAGs; and iv) acylation by DG acyltransferase to synthesize TGs [44]. The conversion of DGs to phosphatidic acid by DG kinase has been reported to be decreased in the AD brain and in the same mechanism may well be responsible for higher levels of selected DG species in VaD [45]. Glycerophospholipid degradation may also play a role in elevations in DG levels in VaD, since PI levels appeared to be lower in VaD as well. Additionally, selective decreases in PE suggest that increased PE degradation may be involved in increased DG levels [46]. Higher DG levels, via enhanced PE degradation, may occur in response to conversion of DG by phospholipase C; breakdown of phosphatidic acid by phospholipase D and conversion to DG by phosphatidic acid phosphatase parallel to deacylation of PE to lysophosphatidylethanolamine (LPE) by PLA2 culminating in the breakdown of LPE to DG [43]. Metabolic syndrome, which is a risk factor for VaD, may also be linked to increased DG levels via increased glycolysis and hyperglycemia is a risk factor for VaD [47]. With regards to SM metabolism, DGs are synthesised from PC by a reaction catalysed by sphingomyelin synthase which involves transfer of the phosphocholine headgroup to a Cer [8], and a significant reduction in the levels of PC was observed in VaD subjects.

We also found significantly higher plasma TG levels in VaD subjects. Numerous studies have indicated a relationship between high TG levels and poor cognitive performance in diabetic patients [48], [49]. A similar finding was reported in the Honolulu-Asia Aging Study in which a 1 SD increase in TG levels during midlife significantly increased the risk of dementia a quarter of a decade later [50]. Increased TG is also an independent risk factor for CVD independent of HDL cholesterol. However, the exact mechanism by which TG can contribute to dementia remains unclear, and most studies have focussed on total or LDL cholesterol with a large variance in results [51]. Our findings suggest that higher levels of plasma TGs indicate that TG pools may also contribute to increased DGs in VaD and may be involved in underlying cognitive dysfunction in VaD.

### 4.2 Changes in the lipidome and risk for VaD

Although the underlying pathobiology and risk factors for incident VaD remain unclear, several potentially modifiable risk factors have been identified. For example, mid-life hypertension increases the likelihood of developing dementia. As well, diabetes can also increase the risk of developing dementia. We therefore aimed to investigate whether there is any prospective association between the lipidomic signature and hypertension and diabetes as two major risk factors for VaD.

We observed a significant decrease in Cer levels in hypertensive patients in both VaD and control groups. There was no significant difference in Cer in VaD with or without presence of hypertension or diabetes. However, all VaD patients showed a higher trend of Cer under the influence of these 2 risk factors. This is important since increased Cer can contribute to vasoconstriction and ischaemic injury through increased levels of TXA2 release in the vasculature [52]. Increased Cer can also impair the activity of glycogen synthase 3 activity which may lead to some of the physiological changes observed in type 2 diabetes and VaD [53]. In contrast to Cer, SMs were increased in VaD patients with a high blood pressure. Almost all SMs increased in the presence of hypertension, leading to altered vasomotor function which can increase the risk of developing incident VaD [54].

We also found an increase in ChE in diabetic patients and no significant changes were reported in hypertensive patients. A previous study in type 1 diabetic subjects showed that LCAT activity was positively correlated with cholesterol ester transfer protein (CETP) activity, total cholesterol, free cholesterol, LDL-C, CETP concentration, and LDL-cholesteryl ester, while it was negatively correlated with cholesteryl ester to free cholesterol ratio [55]. As well, increased serum cholesterol esterification rate has been shown to be an important predictor for developing diabetes [55], [56]. These results suggest that accelerated LCAT and CETP may be due to increased accumulation of LDL-ChE in diabetes but not VaD.

Phospholipids also play an important role in both hypertension and diabetes. We found an increase in LPC in hypertensive patients compared to diabetics. LPC can activate several secondary messenger signalling molecules and stimulate intracellular Ca2+ influx contributing to vascular injury. Increased LPC can impair the endothelium-dependent relaxations mediated by endothelium-derived relaxing factors and influence contractile responses in vascular smooth muscle [57]. On the other hand, reduced LPC levels have been inversely correlated with insulin resistance although (20:X) was reduced in diabetics [58]. Since LPC20:X can induce prostacyclin production, reduced levels are likely to promote platelet activation and further enhance vasoconstriction and vascular injury in diabetic subjects [59].

### 4.3 The lipidome signature as a predictor of VaD

We also examined whether a battery of plasma lipids can be used to discriminate VaD patients from controls. All individual lipids, 9 subgroup lipids and group lipids were used in the random forest algorithm. We observed that 4 classification models, using either ChE & Cer, ChE, Cer or group lipids, had significant power in discriminating VaD from controls, with >85% AUC and >80% sensitivity and specificity. Consistent with results from our association analysis, Cer and ChE ranked as the top two in the order of importance for classification of VaD. Additionally, individual lipids in Cer, ChE and combined Cer and ChE showed even higher AUC than group lipids. Therefore, measurement of these group lipids using mass spectrometry will provide further clues for elucidating the underlying mechanisms of VaD and provide additional drug targets. Taken together, our findings suggest that alterations in both cholesterol and sphingolipid metabolism may play an important role in the pathobiology of VaD.

### 4.4 Application of VaD classification model by lipids in an independent sample

We applied our VaD discrimination model to the OATS dataset which, while comprising non-demented individuals, had many with significant cerebrovascular pathology as determined by MRI. The plasma lipid levels were used to divide the sample into ‘vascular’ and ‘normal’ groups, which were shown to differ in cognitive function, small vessel disease burden and DTI measures. In particular, the two groups differed in attention, processing speed and executive function, cognitive domains known to be preferentially affected in vascular cognitive disorders. While the WMH burden was not different between the two groups, they differed on PSMD, which has been shown to be an excellent marker of cerebrovascular pathology [5]. Since this was a relatively healthy sample, further validation in VaD cases is needed in independent studies.

### 4.5 Association of lipids to cerebral vascular disease and Alzheimer’s disease

Vascular dementia was mainly contributed by cerebral vascular disease (CVD). Most studies have shown an inverse relationship between dyslipidemia and both WMH and cerebral microbleeds, providing evidence that lower lipid levels may play a role in cerebral small vessel disease [60]. Increasing plasma triglycerides have been related to larger WMHs volumes and severity of lacunes [61], which is similar to vascular dementia lipid changes in our study. Recent study on relationship between sphingolipids and WMHs observed SM 38:1 and Cer 34:1 significantly correlated with higher degree of WMHs [62]. Besides, there is a direct relationship between cholesterol levels and ischemic stroke. However, there is no complete lipidomics study on cerebral vascular disease. Future studies are need to discriminate lipids of CVD and VaD and therefore provide clues on mechanisms processing from cerebral vascular disease to vascular dementia [60], [61], [62].

VaD and Alzheimer’s disease share a series of risk factors and pathobiological mechanism and always coexist in elderly. Increasing ceramides have been confirmed to be related with AD and amyloid proteins [63], which is opposite to VaD ceramides changes in this study. The correlation of ceramides abnormalities with neurological disorders was in debate and might dependent on the length of its structure. Future studies are need to specify individual ceramide difference between AD and VaD. Consistent with VaD, multiple studies have found reductions in various classes of phospholipids, including PCs, PEs and PIs in AD patients compared with healthy controls [64], which might indicate the similar changes in cell membrane dysfunction in these two types of dementia.

### 4.6 Limitations and future prospective

Firstly, the sample size was around 50 each in VaD and paired control group, which is sufficient power for analysing 9 lipid classes. However, the strength of data is limited for examining the large number of individual lipids, considering the large numbers. Bonferroni correction was used to reduce the bias, although a larger cohort needs to be replicated in the future.

Secondly, we did not have enough data of weight changes or LDL data which were correlated with changed energy intake or food consumption. However, we studied the effects of alcohol consumption and dietary habits on lipid changes between control and VaD. Therefore, the significant difference of Cer, ChE, DG, TG and PE between normal and demented patients after correction of dietary and alcohol still revealed their possible role in the pathobiology of VaD.

Thirdly, the diagnosis of VaD was made by specialist clinicians and supported by imaging of small vessel diseases. However, one cannot exclude the presence of other concomitant pathology in these patients. It is known that many patients diagnosed with VaD have associated AD and other pathologies. These patients were included in our study when their dementia was predominantly of vascular aetiology. It is likely that our metabolite signal may not be VaD specific but may also represent poor health and presence of other underlying comorbid conditions, thus impacting on the relevance of lipid-based biomarkers in future clinical trials. Examining the lipidomic profiles of other disorders such as AD may increase the specificity of our panel. Additionally, cognitively normal controls are not free of cerebrovascular pathology which is very common in this age group [65]. This would reduce the significance of certain lipids.

Fourthly, our study is useful for exploring clinical biomarkers of VaD because plasma lipids are relatively convenient and fast to be obtained. However, plasma lipids may also be affected by damage to peripheral organs as well, which limits the accuracy of any association between changes in plasma lipid levels and brain pathology. The lipidomics of CSF or post-mortem brain samples are in need in the future to fully explore lipid-related mechanisms in VaD.

Finally, the OATS cohort that we used for the validation of our lipids classification model was non-demented. The lipid profile generated by using VaD samples are correlated not only with cerebral vascular changes and related pathobiological mechanisms, but also with the degree of neural death and synaptic loss which are directly related with dementia symptoms. Therefore, independent replication in a larger VaD cohort is needed.

## CRediT authorship contribution statement

**Yue Liu:** Data curation, Formal analysis, Writing - original draft, Writing - review & editing. **Daniel K.Y. Chan:** Resources, Investigation, Project administration. **Anbupalam Thalamuthu:** Data curation, Formal analysis. **Wei Wen:** Data curation. **Jiyang Jiang:** Data curation. **Matthew Paradise:** Data curation. **Teresa Lee:** Data curation. **John Crawford:** Formal analysis. **Matthew Wai Kin Wong:** Methodology, Data curation. **Ying Hua Xu:** Data curation. **Anne Poljak:** Methodology. **Russell Pickford:** Methodology. **Perminder S. Sachdev:** Conceptualization, Supervision, Investigation, Writing - original draft, Project administration, Funding acquisition. **Nady Braidy:** Conceptualization, Supervision, Funding acquisition, Methodology, Investigation, Project administration, Writing - original draft, Writing - review & editing.

## Declaration of Competing Interest

The authors declare that they have no known competing financial interests or personal relationships that could have appeared to influence the work reported in this paper.

## References

[1] Plassman B.L., Langa K.M., Fisher G.G., Heeringa S.G., Weir D.R., Ofstedal M.B. (2007). Prevalence of dementia in the United States: the aging, demographics, and memory study. Neuroepidemiology.

[2] Wallin A., Gottfries C.G., Karlsson I., Svennerholm L. (1989). Decreased myelin lipids in alzheimer's disease and vascular dementia. Acta Neurol Scand.

[3] Roman G.C., Tatemichi T.K., Erkinjuntti T., Cummings J.L., Masdeu J.C., Garcia J.H. (1993). Vascular dementia: diagnostic criteria for research studies. Report of the ninds-airen international workshop. Neurology.

[4] Sachdev P.S., Lammel A., Trollor J.N., Lee T., Wright M.J., Ames D. (2009). A comprehensive neuropsychiatric study of elderly twins: the older australian twins study. Twin Res Hum Genet.

[5] Baykara E., Gesierich B., Adam R., Tuladhar A.M., Biesbroek J.M., Koek H.L. (2016). A novel imaging marker for small vessel disease based on skeletonization of white matter tracts and diffusion histograms. Ann Neurol.

[6] Wong M.W., Braidy N., Poljak A., Pickford R., Thambisetty M., Sachdev P.S. (2017). Dysregulation of lipids in alzheimer's disease and their role as potential biomarkers. Alzheimer's Dementia: J Alzheimer's Assoc.

[7] Hyotylainen T., Oresic M. (2015). Optimizing the lipidomics workflow for clinical studies–practical considerations. Anal Bioanal Chem.

[8] Wong M.W.K., Braidy N., Pickford R., Vafaee F., Crawford J., Muenchhoff J. (2019). Plasma lipidome variation during the second half of the human lifespan is associated with age and sex but minimally with bmi. PLoS One.

[9] Friedman J., Hastie T., Tibshirani R. (2010). Regularization paths for generalized linear models via coordinate descent. J Stat Softw.

[10] Team R.C.R. (2018). A language and environment for statistical. Computing.

[11] Kuhn M. Caret: Classification and regression training. R package version. 2018.

[12] Robin X., Turck N., Hainard A., Tiberti N., Lisacek F., Sanchez J.C. (2011). Proc: an open-source package for r and s+ to analyze and compare roc curves. BMC Bioinf.

[13] Nagelkerke N.J.D. (1991). A note on a general definition of the coefficient of determination. Biometrika.

[14] Tjur T. (2009). Coefficients of determination in logistic regression models—a new proposal: the coefficient of discrimination. Am Statist.

[15] Olsen A.S.B., Faergeman N.J. (2017). Sphingolipids: membrane microdomains in brain development, function and neurological diseases. Open Biol.

[16] Varma V.R., Oommen A.M., Varma S., Casanova R., An Y., Andrews R.M. (2018). Brain and blood metabolite signatures of pathology and progression in alzheimer disease: a targeted metabolomics study. PLoS Med.

[17] Han X., Rozen S., Boyle S.H., Hellegers C., Cheng H., Burke J.R. (2011). Metabolomics in early Alzheimer's disease: Identification of altered plasma sphingolipidome using shotgun lipidomics. PLoS One.

[18] Mielke M.M., Lyketsos C.G. (2010). Alterations of the sphingolipid pathway in Alzheimer's disease: new biomarkers and treatment targets?. Neuromol Med.

[19] Hussain G., Wang J., Rasul A., Anwar H., Imran A., Qasim M. (2019). Role of cholesterol and sphingolipids in brain development and neurological diseases. Lipids Health Dis.

[20] Lam S.M., Wang Y., Duan X., Wenk M.R., Kalaria R.N., Chen C.P. (2014). Brain lipidomes of subcortical ischemic vascular dementia and mixed dementia. Neurobiol Aging.

[21] Jiang X.C., Paultre F., Pearson T.A., Reed R.G., Francis C.K., Lin M. (2000). Plasma sphingomyelin level as a risk factor for coronary artery disease. Arterioscler Thromb Vasc Biol.

[22] Li Z., Zhang H., Liu J., Liang C.P., Li Y., Li Y. (2011). Reducing plasma membrane sphingomyelin increases insulin sensitivity. Mol Cell Biol.

[23] Deevska G.M., Sunkara M., Morris A.J., Nikolova-Karakashian M.N. (2012). Characterization of secretory sphingomyelinase activity, lipoprotein sphingolipid content and ldl aggregation in ldlr-/- mice fed on a high-fat diet. Biosci Rep.

[24] Dasgupta S., Ray S.K. (2017). Diverse biological functions of sphingolipids in the cns: ceramide and sphingosine regulate myelination in developing brain but stimulate demyelination during pathogenesis of multiple sclerosis. J Neurol Psychol.

[25] Schwartz N.U., Linzer R.W., Truman J.P., Gurevich M., Hannun Y.A., Senkal C.E. (2018). Decreased ceramide underlies mitochondrial dysfunction in charcot-marie-tooth 2f. FASEB J.

[26] Zigdon H., Kogot-Levin A., Park J.W., Goldschmidt R., Kelly S., Merrill A.H. (2013). Ablation of ceramide synthase 2 causes chronic oxidative stress due to disruption of the mitochondrial respiratory chain. J Biol Chem.

[27] Zamroziewicz M.K., Zwilling C.E., Barbey A.K. (2016). Inferior prefrontal cortex mediates the relationship between phosphatidylcholine and executive functions in healthy, older adults. Front Aging Neurosci.

[28] Kang M.S., Baek S.H., Chun Y.S., Moore A.Z., Landman N., Berman D. (2013). Modulation of lipid kinase pi4kiialpha activity and lipid raft association of presenilin 1 underlies gamma-secretase inhibition by ginsenoside (20s)-rg3. J Biol Chem.

[29] Ojo J.O., Algamal M., Leary P., Abdullah L., Mouzon B., Evans J.E. (2019). Converging and differential brain phospholipid dysregulation in the pathogenesis of repetitive mild traumatic brain injury and Alzheimer's disease. Front Neurosci.

[30] Wilkins J.M., Trushina E. (2017). Application of metabolomics in Alzheimer's disease. Front Neurol.

[31] Dennis E.A., Cao J., Hsu Y.H., Magrioti V., Kokotos G. (2011). Phospholipase a2 enzymes: physical structure, biological function, disease implication, chemical inhibition, and therapeutic intervention. Chem Rev.

[32] Hicks J.B., Lai Y., Sheng W., Yang X., Zhu D., Sun G.Y. (2008). Amyloid-beta peptide induces temporal membrane biphasic changes in astrocytes through cytosolic phospholipase a2. Biochim Biophys Acta.

[33] Sun G.Y., Shelat P.B., Jensen M.B., He Y., Sun A.Y., Simonyi A. (2010). Phospholipases a2 and inflammatory responses in the central nervous system. Neuromol Med.

[34] Rosa A.O., Rapoport S.I. (2009). Intracellular- and extracellular-derived ca(2+) influence phospholipase a(2)-mediated fatty acid release from brain phospholipids. Biochim Biophys Acta.

[35] Dawaliby R., Trubbia C., Delporte C., Noyon C., Ruysschaert J.M., Van Antwerpen P. (2016). Phosphatidylethanolamine is a key regulator of membrane fluidity in eukaryotic cells. J Biol Chem.

[36] Fullerton M.D., Hakimuddin F., Bonen A., Bakovic M. (2009). The development of a metabolic disease phenotype in ctp: Phosphoethanolamine cytidylyltransferase-deficient mice. J Biol Chem.

[37] Aid S., Vancassel S., Linard A., Lavialle M., Guesnet P. (2005). Dietary docosahexaenoic acid [22: 6(n-3)] as a phospholipid or a triglyceride enhances the potassium chloride-evoked release of acetylcholine in rat hippocampus. J Nutr.

[38] Arancio O. (2008). Pip2: a new key player in Alzheimer's disease. Cellscience.

[39] Wurtman R.J., Cansev M., Sakamoto T., Ulus I. (2010). Nutritional modifiers of aging brain function: Use of uridine and other phosphatide precursors to increase formation of brain synapses. Nutr Rev.

[40] Mapstone M., Cheema A.K., Fiandaca M.S., Zhong X., Mhyre T.R., MacArthur L.H. (2014). Plasma phospholipids identify antecedent memory impairment in older adults. Nat Med.

[41] Proitsi P., Kim M., Whiley L., Pritchard M., Leung R., Soininen H. (2015). Plasma lipidomics analysis finds long chain cholesteryl esters to be associated with Alzheimer's disease. Transl Psychiatry.

[42] Barter P., Rye K.A. (2011). Cholesteryl ester transfer protein inhibition to reduce cardiovascular risk: where are we now?. Trends Pharmacol Sci.

[43] Wood P.L., Medicherla S., Sheikh N., Terry B., Phillipps A., Kaye J.A. (2015). Targeted lipidomics of fontal cortex and plasma diacylglycerols (dag) in mild cognitive impairment and Alzheimer's disease: validation of dag accumulation early in the pathophysiology of Alzheimer's disease. J Alzheimer's Dis: JAD.

[44] Carrasco S., Merida I. (2007). Diacylglycerol, when simplicity becomes complex. Trends Biochem Sci.

[45] Shin J., Xie D., Zhong X.P. (2013). Microrna-34a enhances t cell activation by targeting diacylglycerol kinase zeta. PLoS One.

[46] Chan R.B., Oliveira T.G., Cortes E.P., Honig L.S., Duff K.E., Small S.A. (2012). Comparative lipidomic analysis of mouse and human brain with alzheimer disease. J Biol Chem.

[47] Polewski M.A., Burhans M.S., Zhao M., Colman R.J., Shanmuganayagam D., Lindstrom M.J. (2015). Plasma diacylglycerol composition is a biomarker of metabolic syndrome onset in rhesus monkeys. J Lipid Res.

[48] Luchsinger J.A., Ma Y., Christophi C.A., Florez H., Golden S.H., Hazuda H. (2017). Metformin, lifestyle intervention, and cognition in the diabetes prevention program outcomes study. Diabetes Care.

[49] Parthasarathy V., Frazier D.T., Bettcher B.M., Jastrzab L., Chao L., Reed B. (2017). Triglycerides are negatively correlated with cognitive function in nondemented aging adults. Neuropsychology.

[50] Kalmijn S., Foley D., White L., Burchfiel C.M., Curb J.D., Petrovitch H. (2000). Metabolic cardiovascular syndrome and risk of dementia in japanese-american elderly men. The honolulu-asia aging study. Arterioscler Thromb Vasc Biol.

[51] Lee J.S., Chang P.Y., Zhang Y., Kizer J.R., Best L.G., Howard B.V. (2017). Triglyceride and hdl-c dyslipidemia and risks of coronary heart disease and ischemic stroke by glycemic dysregulation status: the strong heart study. Diabetes Care.

[52] Bautista-Perez R., del Valle-Mondragon L., Cano-Martinez A., Perez-Mendez O., Escalante B., Franco M. (2015). Involvement of neutral sphingomyelinase in the angiotensin ii signaling pathway. Am J Physiol Renal Physiol.

[53] Sokolowska E., Blachnio-Zabielska A. (2019). The role of ceramides in insulin resistance. Front Endocrinol (Lausanne).

[54] Spijkers L.J., van den Akker R.F., Janssen B.J., Debets J.J., De Mey J.G., Stroes E.S. (2011). Hypertension is associated with marked alterations in sphingolipid biology: a potential role for ceramide. PLoS One.

[55] Martinelli A.E.M., Maranhao R.C., Carvalho P.O., Freitas F.R., Silva B.M.O., Curiati M.N.C. (2018). Cholesteryl ester transfer protein (cetp), hdl capacity of receiving cholesterol and status of inflammatory cytokines in patients with severe heart failure. Lipids Health Dis.

[56] Tanaka S., Yasuda T., Ishida T., Fujioka Y., Tsujino T., Miki T. (2013). Increased serum cholesterol esterification rates predict coronary heart disease and sudden death in a general population. Arterioscler Thromb Vasc Biol.

[57] Miwa Y., Hirata K., Kawashima S., Akita H., Yokoyama M. (1997). Lysophosphatidylcholine inhibits receptor-mediated ca2+ mobilization in intact endothelial cells of rabbit aorta. Arterioscler Thromb Vasc Biol.

[58] Barber M.N., Risis S., Yang C., Meikle P.J., Staples M., Febbraio M.A. (2012). Plasma lysophosphatidylcholine levels are reduced in obesity and type 2 diabetes. PLoS One.

[59] Kaur R., Kaur M., Singh J. (2018). Endothelial dysfunction and platelet hyperactivity in type 2 diabetes mellitus: molecular insights and therapeutic strategies. Cardiovasc Diabetol.

[60] Yaghi S., Elkind M.S. (2015). Lipids and cerebrovascular disease: research and practice. Stroke.

[61] Schilling S., Tzourio C., Dufouil C., Zhu Y., Berr C., Alperovitch A. (2014). Plasma lipids and cerebral small vessel disease. Neurology.

[62] Azizkhanian I., Sheth S.A., Iavarone A.T., Lee S., Kakarla V., Hinman J.D. (2019). Plasma lipid profiling identifies biomarkers of cerebral microvascular disease. Front Neurol.

[63] He X., Huang Y., Li B., Gong C.X., Schuchman E.H. (2010). Deregulation of sphingolipid metabolism in Alzheimer's disease. Neurobiol Aging.

[64] Kosicek M., Hecimovic S. (2013). Phospholipids and Alzheimer's disease: alterations, mechanisms and potential biomarkers. Int J Mol Sci.

[65] Matsumoto M. (2006). cerebrovascular disease in the elderly people. Nihon Ronen Igakkai Zasshi.

