# Supplementary material for: Plasma lipidomic biomarker analysis reveals distinct lipid changes in vascular dementia
Source: Comput Struct Biotechnol J. 2020 Jun 9;18:1613–24. doi: 10.1016/j.csbj.2020.06.001 (PMC7334482; doi:10.1016/j.csbj.2020.06.001)
Supplement: Supplementary data 1 [file mmc1.docx]

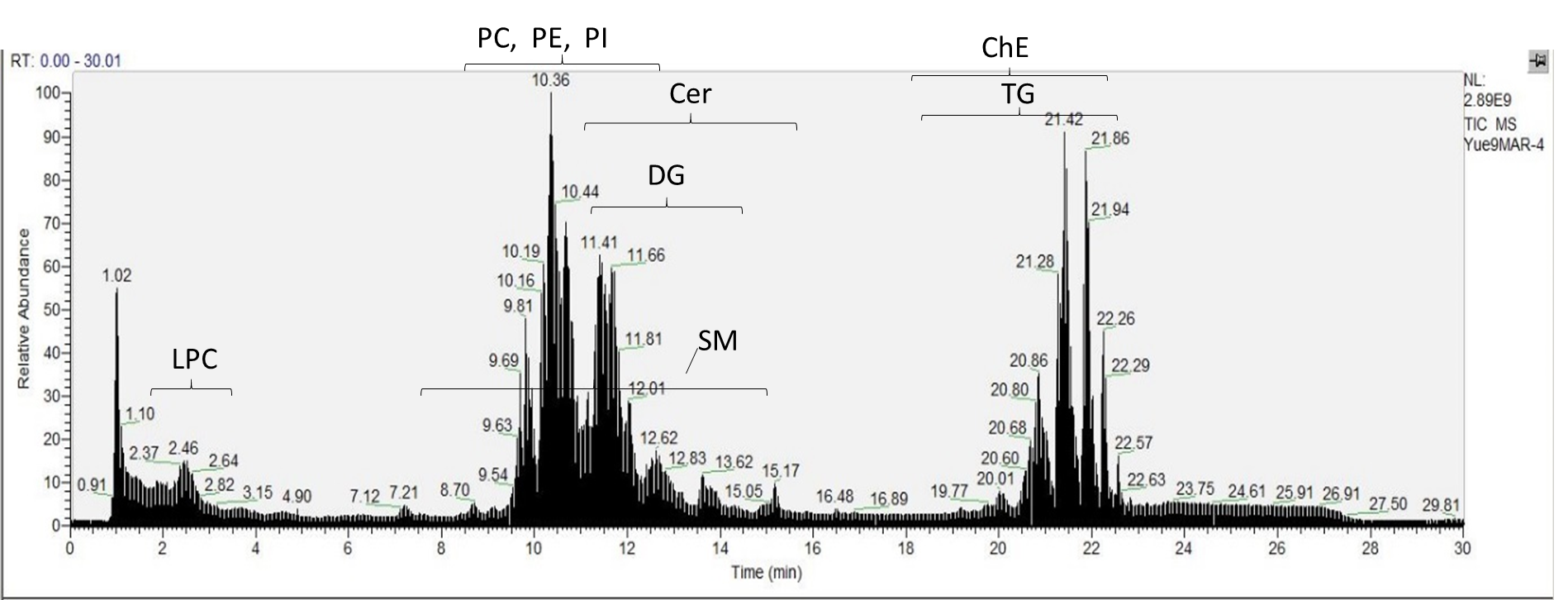


Supplementary figure 1. Corresponding plasma sample in positive ion mode. Typical retention time ranges of elution (minutes) are provided for various lipid classes.
