# Supplementary material for: Plasma lipidomic biomarker analysis reveals distinct lipid changes in vascular dementia
Source: Comput Struct Biotechnol J. 2020 Jun 9;18:1613–24. doi: 10.1016/j.csbj.2020.06.001 (PMC7334482; doi:10.1016/j.csbj.2020.06.001)
Supplement: Supplementary data 2 [file mmc2.docx]

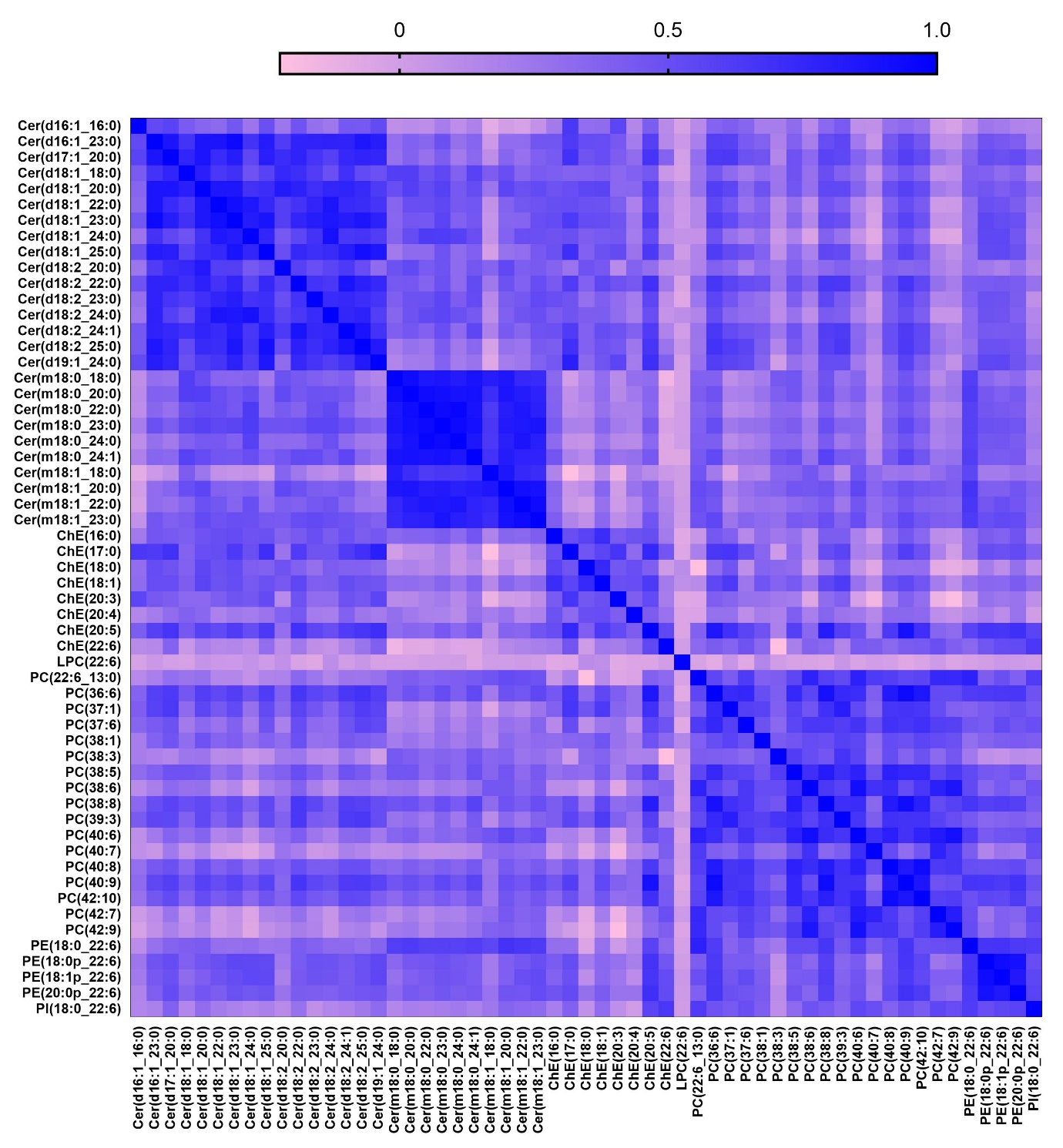


**Supplementary figure 2.** **Partial correlation among significant lipids** (adjusted for disease groups, age, sex, diabetes and hypertension)
