# Supplementary material for: Plasma lipidomic biomarker analysis reveals distinct lipid changes in vascular dementia
Source: Comput Struct Biotechnol J. 2020 Jun 9;18:1613–24. doi: 10.1016/j.csbj.2020.06.001 (PMC7334482; doi:10.1016/j.csbj.2020.06.001)
Supplement: Supplementary data 3 [file mmc3.docx]

Supplementary table 1. Mean value of significant sqrt transformed lipid species (Unianova analysis adjusted for age, sex, diabetes, hypertension)

| **Lipids** | **Control** | | **VaD** | | **MD**  **(VaD-Control)** | **P value** |
| --- | --- | --- | --- | --- | --- | --- |
|  | **Mean** | **SE** | **Mean** | **SE** |  |  |
| **Cer** | | | | | | |
| Cer(d16:1_16:0) | 0.129475 | 0.004425 | 0.101613 | 0.004472 | -9.27E-01 | 3.02E-05 |
| Cer(d17:1_20:0) | 0.090037 | 0.002852 | 0.063968 | 0.002882 | -1.35E+00 | 7.00E-09 |
| Cer(d18:1_18:0) | 0.291658 | 0.008034 | 0.232823 | 0.008119 | -1.08E+00 | 1.78E-06 |
| Cer(d18:1_24:0) | 1.150984 | 0.026497 | 0.904584 | 0.026776 | -1.37E+00 | 4.00E-09 |
| Cer(d18:1_24:1) | 0.920033 | 0.029231 | 0.738418 | 0.029539 | -9.14E-01 | 3.77E-05 |
| Cer(d18:1_25:0) | 0.257921 | 0.006486 | 0.204955 | 0.006554 | -1.20E+00 | 1.49E-07 |
| Cer(d18:1_26:1) | 0.111728 | 0.004119 | 0.086873 | 0.004162 | -8.88E-01 | 5.99E-05 |
| Cer(d18:2_22:0) | 0.204075 | 0.007644 | 0.146697 | 0.007724 | -1.10E+00 | 1.05E-06 |
| Cer(d18:2_24:1) | 0.371872 | 0.010974 | 0.281297 | 0.011089 | -1.21E+00 | 1.14E-07 |
| Cer(d18:2_25:0) | 0.126402 | 0.003531 | 0.092786 | 0.003568 | -1.40E+00 | 2.00E-09 |
| Cer(d19:1_24:0) | 0.319029 | 0.010032 | 0.242881 | 0.010138 | -1.12E+00 | 8.21E-07 |
| Cer(m18:0_18:0) | 0.088449 | 0.003415 | 0.059538 | 0.003451 | -1.25E+00 | 6.00E-08 |
| Cer(m18:0_20:0) | 0.099802 | 0.003439 | 0.066997 | 0.003475 | -1.40E+00 | 2.00E-09 |
| Cer(m18:0_22:0) | 0.146191 | 0.004574 | 0.102859 | 0.004622 | -1.39E+00 | 3.00E-09 |
| Cer(m18:0_24:1) | 0.118354 | 0.005164 | 0.082572 | 0.005218 | -1.02E+00 | 5.41E-06 |
| Cer(m18:1_18:0) | 0.08768 | 0.004264 | 0.055517 | 0.004308 | -1.11E+00 | 9.41E-07 |
| Cer(m18:1_24:1) | 0.19619 | 0.006559 | 0.155567 | 0.006628 | -9.12E-01 | 3.97E-05 |
| **ChE** | | | | | | |
| ChE(16:0) | 3.206909 | 0.062071 | 2.723681 | 0.062725 | -1.15E+00 | 4.64E-07 |
| ChE(17:0) | 0.579874 | 0.020429 | 0.447322 | 0.020644 | -9.55E-01 | 1.81E-05 |
| ChE(18:0) | 1.678965 | 0.035614 | 1.38123 | 0.035989 | -1.23E+00 | 8.30E-08 |
| ChE(18:1) | 9.072682 | 0.176291 | 7.512771 | 0.178149 | -1.30E+00 | 1.80E-08 |
| ChE(20:3) | 3.902688 | 0.098913 | 3.263353 | 0.099956 | -9.51E-01 | 1.94E-05 |
| ChE(20:4) | 14.07438 | 0.30035 | 11.87958 | 0.303515 | -1.08E+00 | 1.86E-06 |
| ChE(20:5) | 4.593777 | 0.193176 | 3.030475 | 0.195212 | -1.19E+00 | 1.86E-07 |
| ChE(22:6) | 4.749246 | 0.14742 | 3.786178 | 0.148974 | -9.62E-01 | 1.61E-05 |
| **DG** | | | | | | |
| DG(12:0_20:5) | 1.028787 | 0.083752 | 1.570017 | 0.084635 | 9.51E-01 | 1.95E-05 |
| DG(18:0_18:0) | 0.691529 | 0.05154 | 1.111271 | 0.052083 | 1.20E+00 | 1.59E-07 |
| **LPC** | | | | | | |
| LPC(22:6) | 0.377303 | 0.019649 | 0.54474 | 0.019856 | 1.25E+00 | 5.10E-08 |
| LPC(24:0) | 0.088116 | 0.002939 | 0.105625 | 0.00297 | 8.77E-01 | 7.30E-05 |
| **PC** | | | | | | |
| PC(22:6_13:0) | 0.48828 | 0.016694 | 0.371374 | 0.01687 | -1.03E+00 | 4.41E-06 |
| PC(34:5) | 0.102598 | 0.005405 | 0.068424 | 0.005462 | -9.31E-01 | 2.82E-05 |
| PC(36:5) | 1.738419 | 0.067853 | 1.306342 | 0.068568 | -9.37E-01 | 2.50E-05 |
| PC(36:6) | 0.277119 | 0.00994 | 0.191833 | 0.010044 | -1.26E+00 | 4.20E-08 |
| PC(37:1) | 0.204193 | 0.007829 | 0.150695 | 0.007912 | -1.01E+00 | 7.07E-06 |
| PC(37:6) | 0.278331 | 0.010498 | 0.200291 | 0.010609 | -1.09E+00 | 1.29E-06 |
| PC(38:1) | 0.262856 | 0.011935 | 0.187625 | 0.012061 | -9.28E-01 | 2.97E-05 |
| PC(38:3) | 2.463355 | 0.060377 | 2.103478 | 0.061013 | -8.77E-01 | 7.26E-05 |
| PC(38:5) | 1.99513 | 0.069088 | 1.490765 | 0.069816 | -1.07E+00 | 1.90E-06 |
| PC(38:6) | 3.795809 | 0.102627 | 3.070746 | 0.103708 | -1.04E+00 | 3.70E-06 |
| PC(38:8) | 0.12395 | 0.00459 | 0.09305 | 0.004638 | -9.91E-01 | 9.34E-06 |
| PC(39:3) | 0.145809 | 0.004575 | 0.112982 | 0.004623 | -1.06E+00 | 2.71E-06 |
| PC(40:6) | 2.07241 | 0.057536 | 1.556652 | 0.058142 | -1.32E+00 | 1.30E-08 |
| PC(40:7) | 0.997433 | 0.038845 | 0.626358 | 0.039254 | -1.41E+00 | 2.00E-09 |
| PC(40:8) | 0.440858 | 0.009855 | 0.357667 | 0.009959 | -1.24E+00 | 6.50E-08 |
| PC(40:9) | 0.086032 | 0.003471 | 0.057127 | 0.003507 | -1.23E+00 | 9.10E-08 |
| PC(42:10) | 0.183112 | 0.004481 | 0.146222 | 0.004528 | -1.21E+00 | 1.22E-07 |
| PC(42:7) | 0.205557 | 0.007341 | 0.14686 | 0.007418 | -1.18E+00 | 2.48E-07 |
| PC(42:9) | 0.108933 | 0.003316 | 0.087599 | 0.003351 | -9.47E-01 | 2.10E-05 |
| **PE** | | | | | | |
| PE(16:0_22:6) | 0.714113 | 0.029175 | 0.538347 | 0.029483 | -8.87E-01 | 6.16E-05 |
| PE(18:0_22:6) | 0.622197 | 0.025974 | 0.445255 | 0.026248 | -1.00E+00 | 7.50E-06 |
| PE(18:0p_22:6) | 0.730266 | 0.020722 | 0.59088 | 0.020941 | -9.90E-01 | 9.50E-06 |
| PE(18:1p_22:6) | 0.52346 | 0.015148 | 0.417289 | 0.015308 | -1.03E+00 | 4.34E-06 |
| PE(20:0p_22:6) | 0.149114 | 0.004978 | 0.117739 | 0.005031 | -9.28E-01 | 2.98E-05 |
| **PI** | | | | | | |
| PI(18:0_22:6) | 1.029129 | 0.022036 | 0.835389 | 0.022268 | -1.29E+00 | 2.20E-08 |
| **TG** | | | | | | |
| TG(10:0_18:2_18:3) | 0.039956 | 0.01059 | 0.106868 | 0.010701 | 9.30E-01 | 2.85E-05 |
| TG(12:0_12:0_12:0) | 0.076769 | 0.005932 | 0.040963 | 0.005994 | -8.89E-01 | 5.97E-05 |
| TG(12:0_18:2_22:6) | 0.04572 | 0.002689 | 0.027027 | 0.002718 | -1.02E+00 | 5.11E-06 |
| TG(14:0_14:0_22:6) | 0.08001 | 0.003756 | 0.051439 | 0.003796 | -1.12E+00 | 7.84E-07 |
| TG(14:0_18:3_22:6) | 0.037332 | 0.002446 | 0.022414 | 0.002472 | -8.98E-01 | 5.07E-05 |
| TG(15:0_12:0_16:0) | 0.073242 | 0.004593 | 0.038756 | 0.004641 | -1.11E+00 | 1.04E-06 |
| TG(15:0_12:0_18:1) | 0.076406 | 0.004392 | 0.041953 | 0.004438 | -1.15E+00 | 3.89E-07 |
| TG(15:0_12:0_18:2) | 0.084928 | 0.00456 | 0.051519 | 0.004608 | -1.08E+00 | 1.76E-06 |
| TG(15:0_14:0_16:1) | 0.137047 | 0.006863 | 0.090141 | 0.006936 | -1.01E+00 | 7.05E-06 |
| TG(15:0_14:0_18:2) | 0.24899 | 0.009172 | 0.184376 | 0.009269 | -1.04E+00 | 3.92E-06 |
| TG(15:0_14:0_20:5) | 0.026657 | 0.002099 | 0.012916 | 0.002121 | -9.64E-01 | 1.55E-05 |
| TG(15:0_16:1_18:2) | 0.302211 | 0.00777 | 0.255892 | 0.007852 | -8.77E-01 | 7.24E-05 |
| TG(15:0_16:1_18:3) | 0.055808 | 0.002882 | 0.036403 | 0.002912 | -9.91E-01 | 9.32E-06 |
| TG(15:0_18:1_20:5) | 0.089631 | 0.004742 | 0.050497 | 0.004792 | -1.21E+00 | 1.15E-07 |
| TG(15:0_18:1_22:6) | 0.143998 | 0.005718 | 0.101146 | 0.005778 | -1.10E+00 | 1.09E-06 |
| TG(15:0_18:2_22:6) | 0.082762 | 0.004055 | 0.046226 | 0.004098 | -1.33E+00 | 1.10E-08 |
| TG(16:0_14:0_20:5) | 0.145807 | 0.00825 | 0.089425 | 0.008337 | -1.01E+00 | 7.06E-06 |
| TG(16:0_16:0_18:1) | 1.6793 | 0.042907 | 1.978033 | 0.043359 | 1.02E+00 | 4.94E-06 |
| TG(16:0_16:1_18:1) | 2.237965 | 0.037066 | 2.459005 | 0.037456 | 8.78E-01 | 7.21E-05 |
| TG(16:0_16:1_20:5) | 0.160546 | 0.004998 | 0.202641 | 0.005051 | 1.24E+00 | 6.80E-08 |
| TG(16:0_18:1_18:1) | 2.925707 | 0.070485 | 3.525688 | 0.071228 | 1.25E+00 | 5.20E-08 |
| TG(16:0_18:1_18:2) | 3.299132 | 0.087264 | 3.861078 | 0.088184 | 9.48E-01 | 2.07E-05 |
| TG(16:0_18:1_20:5) | 0.136283 | 0.006526 | 0.188277 | 0.006595 | 1.17E+00 | 2.70E-07 |
| TG(16:0_18:1_23:0) | 0.543903 | 0.01374 | 0.643522 | 0.013885 | 1.07E+00 | 2.19E-06 |
| TG(16:0_18:2_20:5) | 0.209425 | 0.008085 | 0.27378 | 0.00817 | 1.17E+00 | 2.76E-07 |
| TG(16:0_20:4_22:6) | 0.16753 | 0.007958 | 0.114338 | 0.008042 | -9.84E-01 | 1.07E-05 |
| TG(16:0_22:6_22:6) | 0.098644 | 0.006266 | 0.041831 | 0.006332 | -1.33E+00 | 9.00E-09 |
| TG(16:0e_16:0_18:2) | 0.083767 | 0.004826 | 0.114901 | 0.004877 | 9.49E-01 | 2.00E-05 |
| TG(18:0_18:1_18:1) | 1.516099 | 0.043678 | 1.810253 | 0.044138 | 9.91E-01 | 9.28E-06 |
| TG(18:0_18:1_20:4) | 0.077932 | 0.002921 | 0.102935 | 0.002952 | 1.26E+00 | 4.50E-08 |
| TG(18:0_18:1_22:6) | 0.446977 | 0.015052 | 0.539644 | 0.015211 | 9.06E-01 | 4.37E-05 |
| TG(18:0_20:5_22:6) | 0.070848 | 0.003711 | 0.045363 | 0.003751 | -1.01E+00 | 6.45E-06 |
| TG(18:0e_16:0_18:1) | 0.042071 | 0.002154 | 0.05701 | 0.002177 | 1.02E+00 | 5.34E-06 |
| TG(18:0e_16:0_20:4) | 0.068986 | 0.004048 | 0.096175 | 0.004091 | 9.89E-01 | 9.77E-06 |
| TG(18:1_12:0_12:0) | 0.39896 | 0.021315 | 0.265412 | 0.021539 | -9.22E-01 | 3.28E-05 |
| TG(18:1_14:0_18:1) | 0.381168 | 0.014975 | 0.253433 | 0.015133 | -1.26E+00 | 4.90E-08 |
| TG(18:1_17:1_22:6) | 1.891827 | 0.061486 | 2.313775 | 0.062134 | 1.01E+00 | 6.52E-06 |
| TG(18:1_18:1_18:1) | 2.094555 | 0.078669 | 2.619358 | 0.079498 | 9.82E-01 | 1.11E-05 |
| TG(18:1_18:1_18:3) | 0.739733 | 0.036441 | 0.963696 | 0.036825 | 9.05E-01 | 4.49E-05 |
| TG(18:1_18:1_20:4) | 0.698688 | 0.030822 | 0.965495 | 0.031147 | 1.27E+00 | 3.30E-08 |
| TG(18:1_18:2_20:4) | 0.136835 | 0.007701 | 0.198853 | 0.007782 | 1.19E+00 | 2.09E-07 |
| TG(18:1_18:2_22:6) | 0.107364 | 0.008136 | 0.171467 | 0.008221 | 1.16E+00 | 3.51E-07 |
| TG(18:1_18:3_20:4) | 0.233962 | 0.005751 | 0.272458 | 0.005812 | 9.85E-01 | 1.04E-05 |
| TG(18:1_20:4_22:0) | 0.51673 | 0.015023 | 0.6372 | 0.015181 | 1.18E+00 | 2.31E-07 |
| TG(18:1_22:5_22:6) | 0.058365 | 0.003967 | 0.030976 | 0.004009 | -1.02E+00 | 5.82E-06 |
| TG(18:2_22:6_22:6) | 0.039246 | 0.002825 | 0.022392 | 0.002855 | -8.78E-01 | 7.17E-05 |
| TG(18:3_18:2_18:3) | 0.194763 | 0.009676 | 0.123251 | 0.009778 | -1.09E+00 | 1.46E-06 |
| TG(18:4_16:0_18:1) | 0.106435 | 0.003848 | 0.142932 | 0.003888 | 1.40E+00 | 2.00E-09 |
| TG(20:0_18:1_18:1) | 0.563839 | 0.020484 | 0.70851 | 0.0207 | 1.04E+00 | 3.73E-06 |
| TG(20:0e_18:1_18:2) | 0.047943 | 0.002733 | 0.067057 | 0.002761 | 1.03E+00 | 4.51E-06 |
| TG(20:5_18:2_18:2) | 0.129147 | 0.00733 | 0.17848 | 0.007407 | 9.91E-01 | 9.39E-06 |
| TG(20:5_18:2_22:6) | 0.036714 | 0.002719 | 0.019262 | 0.002748 | -9.45E-01 | 2.19E-05 |
