# Supplementary material for: Plasma lipidomic biomarker analysis reveals distinct lipid changes in vascular dementia
Source: Comput Struct Biotechnol J. 2020 Jun 9;18:1613–24. doi: 10.1016/j.csbj.2020.06.001 (PMC7334482; doi:10.1016/j.csbj.2020.06.001)
Supplement: Supplementary data 4 [file mmc4.docx]

Supplementary Table 2. Mean and SE value of all sqrt transformed lipid species (Unianova analysisadjusted for age, sex, diabetes, hypertension)

| **Lipid species** | **Control** | | **VaD** | | **SMD (VaD-Control)** | **p-value** |
| --- | --- | --- | --- | --- | --- | --- |
|  | **Mean** | **SE** | **Mean** | **SE** |  |  |
| **Cer** | | | | | | |
| Cer(d16:0_24:1) | 0.06264 | 0.003265 | 0.046575 | 0.0033 | -7.24E-01 | 9.03E-04 |
| Cer(d16:1_16:0) | 0.129475 | 0.004425 | 0.101613 | 0.004472 | -9.27E-01 | 3.02E-05 |
| Cer(d16:1_21:2) | 0.11022 | 0.007296 | 0.124161 | 0.007372 | 2.81E-01 | 1.86E-01 |
| Cer(d16:1_23:0) | 0.243282 | 0.006416 | 0.175859 | 0.006484 | -1.55E+00 | <1E-10 |
| Cer(d17:1_20:0) | 0.090037 | 0.002852 | 0.063968 | 0.002882 | -1.35E+00 | 7.00E-09 |
| Cer(d18:0_16:0) | 0.133914 | 0.004776 | 0.115316 | 0.004826 | -5.73E-01 | 7.90E-03 |
| Cer(d18:0_18:0) | 0.116114 | 0.005394 | 0.100071 | 0.005451 | -4.38E-01 | 4.08E-02 |
| Cer(d18:0_22:0) | 0.224704 | 0.007277 | 0.18829 | 0.007354 | -7.36E-01 | 7.45E-04 |
| Cer(d18:0_23:0) | 0.160704 | 0.005487 | 0.134965 | 0.005545 | -6.90E-01 | 1.51E-03 |
| Cer(d18:0_24:0) | 0.24835 | 0.008048 | 0.20744 | 0.008133 | -7.48E-01 | 6.21E-04 |
| Cer(d18:0_24:1) | 0.176824 | 0.007784 | 0.15542 | 0.007866 | -4.05E-01 | 5.82E-02 |
| Cer(d18:1_16:0) | 0.424339 | 0.009019 | 0.376345 | 0.009114 | -7.83E-01 | 3.54E-04 |
| Cer(d18:1_18:0) | 0.291658 | 0.008034 | 0.232823 | 0.008119 | -1.08E+00 | 1.78E-06 |
| Cer(d18:1_20:0) | 0.377628 | 0.009078 | 0.273643 | 0.009174 | -1.69E+00 | <1E-10 |
| Cer(d18:1_22:0) | 0.933941 | 0.019898 | 0.732001 | 0.020108 | -1.49E+00 | <1E-10 |
| Cer(d18:1_23:0) | 0.642277 | 0.014547 | 0.496109 | 0.014701 | -1.48E+00 | <1E-10 |
| Cer(d18:1_24:0) | 1.150984 | 0.026497 | 0.904584 | 0.026776 | -1.37E+00 | 4.00E-09 |
| Cer(d18:1_24:1) | 0.920033 | 0.029231 | 0.738418 | 0.029539 | -9.14E-01 | 3.77E-05 |
| Cer(d18:1_25:0) | 0.257921 | 0.006486 | 0.204955 | 0.006554 | -1.20E+00 | 1.49E-07 |
| Cer(d18:1_26:1) | 0.111728 | 0.004119 | 0.086873 | 0.004162 | -8.88E-01 | 5.99E-05 |
| Cer(d18:2_20:0) | 0.153467 | 0.003716 | 0.109998 | 0.003755 | -1.72E+00 | <1E-10 |
| Cer(d18:2_22:0) | 0.204075 | 0.007644 | 0.146697 | 0.007724 | -1.10E+00 | 1.05E-06 |
| Cer(d18:2_23:0) | 0.268278 | 0.013451 | 0.196651 | 0.013592 | -7.84E-01 | 3.51E-04 |
| Cer(d18:2_24:0) | 0.565916 | 0.011231 | 0.418686 | 0.01135 | -1.93E+00 | <1E-10 |
| Cer(d18:2_24:1) | 0.371872 | 0.010974 | 0.281297 | 0.011089 | -1.21E+00 | 1.14E-07 |
| Cer(d18:2_25:0) | 0.126402 | 0.003531 | 0.092786 | 0.003568 | -1.40E+00 | 2.00E-09 |
| Cer(d19:1_24:0) | 0.319029 | 0.010032 | 0.242881 | 0.010138 | -1.12E+00 | 8.21E-07 |
| Cer(d19:1_24:1) | 0.200638 | 0.009677 | 0.155102 | 0.009779 | -6.93E-01 | 1.46E-03 |
| Cer(d19:2_23:0+O) | 0.093956 | 0.003868 | 0.074954 | 0.003908 | -7.23E-01 | 9.18E-04 |
| Cer(m18:0_18:0) | 0.088449 | 0.003415 | 0.059538 | 0.003451 | -1.25E+00 | 6.00E-08 |
| Cer(m18:0_20:0) | 0.099802 | 0.003439 | 0.066997 | 0.003475 | -1.40E+00 | 2.00E-09 |
| Cer(m18:0_22:0) | 0.146191 | 0.004574 | 0.102859 | 0.004622 | -1.39E+00 | 3.00E-09 |
| Cer(m18:0_23:0) | 0.090848 | 0.002455 | 0.061815 | 0.002481 | -1.74E+00 | <1E-10 |
| Cer(m18:0_24:0) | 0.147659 | 0.004185 | 0.102856 | 0.004229 | -1.58E+00 | <1E-10 |
| Cer(m18:0_24:1) | 0.118354 | 0.005164 | 0.082572 | 0.005218 | -1.02E+00 | 5.41E-06 |
| Cer(m18:1_18:0) | 0.08768 | 0.004264 | 0.055517 | 0.004308 | -1.11E+00 | 9.41E-07 |
| Cer(m18:1_20:0) | 0.14589 | 0.00491 | 0.093917 | 0.004962 | -1.56E+00 | <1E-10 |
| Cer(m18:1_22:0) | 0.226651 | 0.006512 | 0.151169 | 0.00658 | -1.71E+00 | <1E-10 |
| Cer(m18:1_23:0) | 0.140879 | 0.003889 | 0.092815 | 0.00393 | -1.82E+00 | <1E-10 |
| Cer(m18:1_24:0+O) | 0.086872 | 0.002608 | 0.072608 | 0.002636 | -8.05E-01 | 2.48E-04 |
| Cer(m18:1_24:1) | 0.19619 | 0.006559 | 0.155567 | 0.006628 | -9.12E-01 | 3.97E-05 |
| Cer(m18:1_24:2) | 0.044587 | 0.003238 | 0.036922 | 0.003273 | -3.49E-01 | 1.02E-01 |
| Cer(t16:1_12:0) | 0.166834 | 0.006398 | 0.181496 | 0.006465 | 3.37E-01 | 1.13E-01 |
| Cer(t16:1_16:0) | 0.132876 | 0.003584 | 0.136371 | 0.003622 | 1.44E-01 | 4.98E-01 |
| Cer(t18:0_22:0) | 0.135615 | 0.004211 | 0.114532 | 0.004256 | -7.37E-01 | 7.41E-04 |
| Cer(t18:0_23:0) | 0.160466 | 0.005538 | 0.132575 | 0.005596 | -7.41E-01 | 6.92E-04 |
| Cer(t18:0_24:0) | 0.174621 | 0.005367 | 0.148114 | 0.005423 | -7.27E-01 | 8.64E-04 |
| Cer(t18:0_24:1) | 0.123285 | 0.00483 | 0.102863 | 0.004881 | -6.22E-01 | 4.05E-03 |
| **ChE** | | | | | | |
| ChE(16:0) | 3.206909 | 0.062071 | 2.723681 | 0.062725 | -1.15E+00 | 4.64E-07 |
| ChE(16:1) | 1.920488 | 0.090272 | 1.790043 | 0.091223 | -2.13E-01 | 3.16E-01 |
| ChE(17:0) | 0.579874 | 0.020429 | 0.447322 | 0.020644 | -9.55E-01 | 1.81E-05 |
| ChE(17:1) | 0.788871 | 0.030418 | 0.649172 | 0.030739 | -6.76E-01 | 1.87E-03 |
| ChE(18:0) | 1.678965 | 0.035614 | 1.38123 | 0.035989 | -1.23E+00 | 8.30E-08 |
| ChE(18:1) | 9.072682 | 0.176291 | 7.512771 | 0.178149 | -1.30E+00 | 1.80E-08 |
| ChE(18:2) | 18.36045 | 0.490437 | 17.59051 | 0.495605 | -2.31E-01 | 2.76E-01 |
| ChE(18:3) | 3.464311 | 0.135805 | 2.96483 | 0.137236 | -5.41E-01 | 1.19E-02 |
| ChE(20:2) | 3.389274 | 0.119029 | 3.53021 | 0.120284 | 1.74E-01 | 4.11E-01 |
| ChE(20:3) | 3.902688 | 0.098913 | 3.263353 | 0.099956 | -9.51E-01 | 1.94E-05 |
| ChE(20:4) | 14.07438 | 0.30035 | 11.87958 | 0.303515 | -1.08E+00 | 1.86E-06 |
| ChE(20:5) | 4.593777 | 0.193176 | 3.030475 | 0.195212 | -1.19E+00 | 1.86E-07 |
| ChE(22:4) | 0.902033 | 0.027066 | 0.832212 | 0.027351 | -3.80E-01 | 7.53E-02 |
| ChE(22:5) | 0.845659 | 0.03687 | 0.844914 | 0.037258 | -2.99E-03 | 9.89E-01 |
| ChE(22:6) | 4.749246 | 0.14742 | 3.786178 | 0.148974 | -9.62E-01 | 1.61E-05 |
| **DG** | | | | | | |
| DG(12:0_20:5) | 1.028787 | 0.083752 | 1.570017 | 0.084635 | 9.51E-01 | 1.95E-05 |
| DG(14:0_18:2) | 1.765663 | 0.072836 | 1.814619 | 0.073603 | 9.89E-02 | 6.40E-01 |
| DG(14:0_18:3) | 0.371339 | 0.02289 | 0.422835 | 0.023131 | 3.31E-01 | 1.20E-01 |
| DG(14:0_20:4) | 0.665945 | 0.045424 | 0.568896 | 0.045903 | -3.14E-01 | 1.40E-01 |
| DG(15:0_18:2) | 0.822742 | 0.027851 | 0.76575 | 0.028144 | -3.01E-01 | 1.57E-01 |
| DG(16:0_16:0) | 1.833867 | 0.064515 | 2.158846 | 0.065195 | 7.41E-01 | 6.90E-04 |
| DG(16:0_18:1) | 6.772621 | 0.171539 | 7.719276 | 0.173347 | 8.12E-01 | 2.20E-04 |
| DG(16:0_18:2) | 6.988684 | 0.226287 | 8.221765 | 0.228672 | 8.02E-01 | 2.60E-04 |
| DG(16:0_18:3) | 2.419869 | 0.147684 | 2.549816 | 0.14924 | 1.30E-01 | 5.41E-01 |
| DG(16:0_22:6) | 1.825655 | 0.070259 | 1.49526 | 0.071 | -6.92E-01 | 1.47E-03 |
| DG(16:1_18:2) | 3.010166 | 0.160147 | 3.474013 | 0.161835 | 4.26E-01 | 4.63E-02 |
| DG(16:1_18:3) | 0.96351 | 0.059088 | 0.882297 | 0.059711 | -2.02E-01 | 3.40E-01 |
| DG(17:0_18:1) | 0.601721 | 0.012974 | 0.553635 | 0.01311 | -5.46E-01 | 1.13E-02 |
| DG(17:1_18:1) | 1.218123 | 0.037269 | 1.283885 | 0.037662 | 2.60E-01 | 2.22E-01 |
| DG(17:1_18:2) | 0.950937 | 0.025036 | 0.962501 | 0.0253 | 6.80E-02 | 7.48E-01 |
| DG(18:0_16:0) | 1.472805 | 0.064643 | 1.842165 | 0.065324 | 8.41E-01 | 1.36E-04 |
| DG(18:0_18:0) | 0.691529 | 0.05154 | 1.111271 | 0.052083 | 1.20E+00 | 1.59E-07 |
| DG(18:0_18:1) | 2.928079 | 0.101018 | 3.298402 | 0.102083 | 5.40E-01 | 1.22E-02 |
| DG(18:1_14:0) | 2.35035 | 0.053355 | 2.449304 | 0.053917 | 2.73E-01 | 1.99E-01 |
| DG(18:1_18:1) | 3.746265 | 0.159586 | 4.274785 | 0.161268 | 4.87E-01 | 2.31E-02 |
| DG(18:1_18:2) | 10.90128 | 0.402394 | 11.85233 | 0.406634 | 3.48E-01 | 1.03E-01 |
| DG(18:1_18:3) | 2.17747 | 0.084026 | 2.382939 | 0.084912 | 3.60E-01 | 9.15E-02 |
| DG(18:1_20:3) | 1.772726 | 0.061127 | 1.766495 | 0.061771 | -1.50E-02 | 9.43E-01 |
| DG(18:1_20:4) | 3.943621 | 0.157227 | 4.279857 | 0.158884 | 3.15E-01 | 1.39E-01 |
| DG(18:1_22:4) | 1.107327 | 0.042359 | 1.118047 | 0.042805 | 3.73E-02 | 8.60E-01 |
| DG(18:1_22:5) | 1.851243 | 0.075707 | 1.467526 | 0.076505 | -7.46E-01 | 6.42E-04 |
| DG(18:1_22:6) | 2.666854 | 0.112782 | 2.06679 | 0.113971 | -7.83E-01 | 3.55E-04 |
| DG(18:2_18:2) | 7.540559 | 0.372737 | 8.9016 | 0.376665 | 5.37E-01 | 1.25E-02 |
| DG(18:2_20:4) | 3.801016 | 0.204694 | 4.177789 | 0.206851 | 2.71E-01 | 2.02E-01 |
| DG(18:2_22:6) | 2.126188 | 0.130966 | 1.733677 | 0.132346 | -4.41E-01 | 3.94E-02 |
| DG(18:3_18:2) | 2.828281 | 0.184772 | 3.420221 | 0.18672 | 4.72E-01 | 2.79E-02 |
| DG(20:0_18:1) | 0.623856 | 0.047016 | 0.664749 | 0.047511 | 1.28E-01 | 5.46E-01 |
| DG(20:0_18:2) | 0.350936 | 0.037514 | 0.358393 | 0.037909 | 2.93E-02 | 8.90E-01 |
| DG(20:1_18:1) | 1.190071 | 0.044175 | 1.177903 | 0.044641 | -4.06E-02 | 8.48E-01 |
| DG(20:1_18:2) | 1.407646 | 0.048736 | 1.549588 | 0.049249 | 4.29E-01 | 4.51E-02 |
| DG(20:3_18:2) | 1.756276 | 0.07736 | 1.831843 | 0.078175 | 1.44E-01 | 4.97E-01 |
| DG(20:5_18:2) | 2.011847 | 0.125786 | 1.632537 | 0.127112 | -4.44E-01 | 3.82E-02 |
| DG(22:3e) | 1.34661 | 0.088131 | 1.109164 | 0.08906 | -3.97E-01 | 6.34E-02 |
| DG(22:5_18:2) | 1.42303 | 0.072806 | 1.293734 | 0.073573 | -2.61E-01 | 2.19E-01 |
| DG(29:3) | 1.52954 | 0.109228 | 2.086592 | 0.110379 | 7.51E-01 | 5.97E-04 |
| DG(29:4) | 1.274535 | 0.10115 | 1.614083 | 0.102216 | 4.94E-01 | 2.14E-02 |
| DG(33:4) | 1.70227 | 0.223837 | 2.909557 | 0.226195 | 7.94E-01 | 2.98E-04 |
| **LPC** | | | | | | |
| LPC(14:0) | 0.346284 | 0.020059 | 0.459728 | 0.02027 | 8.32E-01 | 1.57E-04 |
| LPC(15:0) | 0.329124 | 0.015077 | 0.411301 | 0.015236 | 8.02E-01 | 2.59E-04 |
| LPC(16:0) | 2.992829 | 0.144617 | 3.205917 | 0.146141 | 2.17E-01 | 3.07E-01 |
| LPC(16:0e) | 0.293447 | 0.012647 | 0.311726 | 0.01278 | 2.13E-01 | 3.16E-01 |
| LPC(16:1) | 0.485211 | 0.025748 | 0.542756 | 0.026019 | 3.29E-01 | 1.22E-01 |
| LPC(17:0) | 0.430588 | 0.019402 | 0.533619 | 0.019607 | 7.82E-01 | 3.64E-04 |
| LPC(17:1) | 0.117405 | 0.011607 | 0.151039 | 0.01173 | 4.26E-01 | 4.62E-02 |
| LPC(18:0) | 1.489948 | 0.106511 | 1.820524 | 0.107634 | 4.57E-01 | 3.30E-02 |
| LPC(18:0e) | 0.111775 | 0.010214 | 0.106437 | 0.010321 | -7.70E-02 | 7.16E-01 |
| LPC(18:1) | 1.034332 | 0.102055 | 1.088147 | 0.10313 | 7.76E-02 | 7.14E-01 |
| LPC(18:2) | 1.425637 | 0.10437 | 1.743823 | 0.10547 | 4.49E-01 | 3.62E-02 |
| LPC(18:3) | 0.028214 | 0.002583 | 0.034571 | 0.00261 | 3.62E-01 | 8.94E-02 |
| LPC(20:0) | 0.091246 | 0.008914 | 0.104796 | 0.009008 | 2.24E-01 | 2.92E-01 |
| LPC(20:1) | 0.180712 | 0.00734 | 0.217038 | 0.007418 | 7.29E-01 | 8.46E-04 |
| LPC(20:2) | 0.108644 | 0.010446 | 0.104798 | 0.010556 | -5.43E-02 | 7.98E-01 |
| LPC(20:3) | 0.031932 | 0.002912 | 0.039045 | 0.002943 | 3.59E-01 | 9.19E-02 |
| LPC(20:4) | 0.081837 | 0.009708 | 0.116097 | 0.00981 | 5.19E-01 | 1.57E-02 |
| LPC(20:5) | 0.077333 | 0.007301 | 0.065566 | 0.007378 | -2.37E-01 | 2.64E-01 |
| LPC(22:0) | 0.063431 | 0.002406 | 0.074452 | 0.002432 | 6.74E-01 | 1.92E-03 |
| LPC(22:1) | 0.04809 | 0.002976 | 0.064671 | 0.003007 | 8.20E-01 | 1.93E-04 |
| LPC(22:4) | 0.091414 | 0.00951 | 0.098571 | 0.00961 | 1.11E-01 | 6.01E-01 |
| LPC(22:5) | 0.234541 | 0.010396 | 0.270418 | 0.010506 | 5.08E-01 | 1.81E-02 |
| LPC(22:6) | 0.377303 | 0.019649 | 0.54474 | 0.019856 | 1.25E+00 | 5.10E-08 |
| LPC(24:0) | 0.088116 | 0.002939 | 0.105625 | 0.00297 | 8.77E-01 | 7.30E-05 |
| LPC(24:1) | 0.021192 | 0.001308 | 0.027448 | 0.001322 | 7.04E-01 | 1.23E-03 |
| LPC(26:0) | 0.074477 | 0.00443 | 0.093888 | 0.004477 | 6.45E-01 | 2.94E-03 |
| **PC** | | | | | | |
| PC(15:0_18:2) | 0.704491 | 0.020388 | 0.641014 | 0.020603 | -4.58E-01 | 3.25E-02 |
| PC(15:0_20:4) | 0.882169 | 0.027794 | 0.739836 | 0.028087 | -7.54E-01 | 5.68E-04 |
| PC(16:1_18:1) | 0.847273 | 0.167366 | 0.878804 | 0.16913 | 2.77E-02 | 8.96E-01 |
| PC(16:1_22:5) | 0.021731 | 0.001052 | 0.017346 | 0.001063 | -6.13E-01 | 4.57E-03 |
| PC(18:1_13:0) | 0.332476 | 0.011597 | 0.285527 | 0.011719 | -5.96E-01 | 5.83E-03 |
| PC(18:1_18:1) | 0.910194 | 0.090047 | 0.93376 | 0.090996 | 3.85E-02 | 8.56E-01 |
| PC(18:2_13:0) | 0.43406 | 0.012156 | 0.401757 | 0.012284 | -3.91E-01 | 6.70E-02 |
| PC(18:2_18:2) | 5.150241 | 0.134273 | 4.687669 | 0.135688 | -5.07E-01 | 1.83E-02 |
| PC(18:2_20:4) | 0.099044 | 0.006229 | 0.094327 | 0.006295 | -1.12E-01 | 5.99E-01 |
| PC(20:2_18:2) | 0.065153 | 0.005609 | 0.060458 | 0.005668 | -1.23E-01 | 5.61E-01 |
| PC(21:3e) | 0.072626 | 0.007725 | 0.084526 | 0.007806 | 2.27E-01 | 2.85E-01 |
| PC(22:6_13:0) | 0.48828 | 0.016694 | 0.371374 | 0.01687 | -1.03E+00 | 4.41E-06 |
| PC(26:0) | 0.109225 | 0.006765 | 0.123363 | 0.006836 | 3.08E-01 | 1.48E-01 |
| PC(28:0) | 0.170662 | 0.009115 | 0.127129 | 0.009211 | -7.03E-01 | 1.25E-03 |
| PC(28:1) | 0.024329 | 0.002311 | 0.017694 | 0.002336 | -4.22E-01 | 4.83E-02 |
| PC(29:0) | 0.093119 | 0.005315 | 0.06516 | 0.005371 | -7.74E-01 | 4.09E-04 |
| PC(30:0) | 0.694546 | 0.026891 | 0.601129 | 0.027175 | -5.11E-01 | 1.74E-02 |
| PC(30:0e) | 0.116975 | 0.009479 | 0.104859 | 0.009579 | -1.88E-01 | 3.75E-01 |
| PC(30:1) | 0.182887 | 0.010937 | 0.143226 | 0.011053 | -5.34E-01 | 1.31E-02 |
| PC(30:1e) | 0.111217 | 0.005766 | 0.100122 | 0.005826 | -2.83E-01 | 1.83E-01 |
| PC(30:2) | 0.046894 | 0.002911 | 0.036294 | 0.002942 | -5.36E-01 | 1.28E-02 |
| PC(31:0) | 0.072851 | 0.003376 | 0.057787 | 0.003411 | -6.57E-01 | 2.48E-03 |
| PC(31:0e) | 0.057584 | 0.004612 | 0.056021 | 0.00466 | -4.98E-02 | 8.14E-01 |
| PC(31:1) | 0.152459 | 0.007568 | 0.124024 | 0.007648 | -5.53E-01 | 1.03E-02 |
| PC(32:0) | 1.386703 | 0.043788 | 1.253661 | 0.044249 | -4.47E-01 | 3.68E-02 |
| PC(32:0e) | 0.58026 | 0.01759 | 0.554168 | 0.017775 | -2.18E-01 | 3.04E-01 |
| PC(32:1) | 1.379243 | 0.05665 | 1.226461 | 0.057247 | -3.97E-01 | 6.31E-02 |
| PC(32:1e) | 0.276703 | 0.009771 | 0.274768 | 0.009874 | -2.92E-02 | 8.90E-01 |
| PC(32:2) | 0.678816 | 0.030658 | 0.615146 | 0.030981 | -3.06E-01 | 1.51E-01 |
| PC(32:3) | 0.021202 | 0.001695 | 0.017129 | 0.001713 | -3.53E-01 | 9.72E-02 |
| PC(33:0) | 0.35111 | 0.014829 | 0.301868 | 0.014985 | -4.89E-01 | 2.28E-02 |
| PC(33:0e) | 0.03915 | 0.004031 | 0.040254 | 0.004074 | 4.02E-02 | 8.49E-01 |
| PC(33:1) | 0.733924 | 0.026893 | 0.599194 | 0.027176 | -7.37E-01 | 7.35E-04 |
| PC(33:2) | 0.701561 | 0.031777 | 0.716366 | 0.032112 | 6.86E-02 | 7.46E-01 |
| PC(33:3) | 0.091146 | 0.004777 | 0.08009 | 0.004827 | -3.41E-01 | 1.10E-01 |
| PC(33:4) | 0.111459 | 0.005593 | 0.129332 | 0.005652 | 4.70E-01 | 2.83E-02 |
| PC(33:5) | 0.094866 | 0.005111 | 0.066574 | 0.005165 | -8.15E-01 | 2.11E-04 |
| PC(34:0) | 0.700155 | 0.020276 | 0.615297 | 0.02049 | -6.16E-01 | 4.42E-03 |
| PC(34:0e) | 0.238838 | 0.008807 | 0.212208 | 0.008899 | -4.45E-01 | 3.77E-02 |
| PC(34:1) | 5.184064 | 0.154226 | 4.870143 | 0.155851 | -3.00E-01 | 1.59E-01 |
| PC(34:1e) | 0.770845 | 0.034377 | 0.673893 | 0.03474 | -4.15E-01 | 5.22E-02 |
| PC(34:2) | 7.155615 | 0.235465 | 7.081141 | 0.237946 | -4.66E-02 | 8.26E-01 |
| PC(34:2e) | 0.68114 | 0.022048 | 0.722799 | 0.02228 | 2.78E-01 | 1.91E-01 |
| PC(34:3) | 0.667253 | 0.046825 | 0.666453 | 0.047319 | -2.51E-03 | 9.91E-01 |
| PC(34:4) | 0.092483 | 0.006447 | 0.075312 | 0.006515 | -3.92E-01 | 6.64E-02 |
| PC(34:5) | 0.102598 | 0.005405 | 0.068424 | 0.005462 | -9.31E-01 | 2.82E-05 |
| PC(35:0) | 0.12498 | 0.007023 | 0.095595 | 0.007097 | -6.16E-01 | 4.43E-03 |
| PC(35:1) | 0.806133 | 0.029392 | 0.69994 | 0.029702 | -5.32E-01 | 1.35E-02 |
| PC(35:2) | 0.085742 | 0.008062 | 0.063188 | 0.008147 | -4.12E-01 | 5.41E-02 |
| PC(35:3) | 0.08233 | 0.006117 | 0.073156 | 0.006182 | -2.21E-01 | 2.98E-01 |
| PC(35:5) | 0.215629 | 0.015264 | 0.182835 | 0.015425 | -3.16E-01 | 1.37E-01 |
| PC(35:6) | 0.095495 | 0.003807 | 0.091373 | 0.003848 | -1.59E-01 | 4.52E-01 |
| PC(36:0) | 0.254999 | 0.006705 | 0.215924 | 0.006775 | -8.58E-01 | 1.02E-04 |
| PC(36:0e) | 0.028597 | 0.002221 | 0.023662 | 0.002244 | -3.27E-01 | 1.25E-01 |
| PC(36:1) | 2.39016 | 0.065642 | 2.167595 | 0.066334 | -4.99E-01 | 2.01E-02 |
| PC(36:1e) | 0.196824 | 0.01101 | 0.202692 | 0.011126 | 7.85E-02 | 7.11E-01 |
| PC(36:2) | 5.562486 | 0.132679 | 5.329245 | 0.134077 | -2.59E-01 | 2.23E-01 |
| PC(36:2e) | 0.555348 | 0.017422 | 0.546495 | 0.017606 | -7.48E-02 | 7.24E-01 |
| PC(36:3) | 1.443425 | 0.03781 | 1.308916 | 0.038208 | -5.24E-01 | 1.49E-02 |
| PC(36:4) | 2.088361 | 0.070252 | 2.103081 | 0.070993 | 3.08E-02 | 8.84E-01 |
| PC(36:4e) | 0.062988 | 0.003974 | 0.063282 | 0.004016 | 1.07E-02 | 9.59E-01 |
| PC(36:5) | 1.738419 | 0.067853 | 1.306342 | 0.068568 | -9.37E-01 | 2.50E-05 |
| PC(36:6) | 0.277119 | 0.00994 | 0.191833 | 0.010044 | -1.26E+00 | 4.20E-08 |
| PC(37:1) | 0.204193 | 0.007829 | 0.150695 | 0.007912 | -1.01E+00 | 7.07E-06 |
| PC(37:2) | 0.443721 | 0.012574 | 0.386372 | 0.012706 | -6.71E-01 | 2.00E-03 |
| PC(37:3) | 0.566188 | 0.015567 | 0.495974 | 0.015731 | -6.64E-01 | 2.23E-03 |
| PC(37:4) | 0.09094 | 0.005203 | 0.079153 | 0.005258 | -3.34E-01 | 1.18E-01 |
| PC(37:5) | 0.204592 | 0.007934 | 0.163425 | 0.008017 | -7.64E-01 | 4.85E-04 |
| PC(37:6) | 0.278331 | 0.010498 | 0.200291 | 0.010609 | -1.09E+00 | 1.29E-06 |
| PC(38:1) | 0.262856 | 0.011935 | 0.187625 | 0.012061 | -9.28E-01 | 2.97E-05 |
| PC(38:2) | 0.681917 | 0.026391 | 0.607515 | 0.026669 | -4.15E-01 | 5.23E-02 |
| PC(38:2e) | 0.067816 | 0.005837 | 0.063054 | 0.005898 | -1.20E-01 | 5.71E-01 |
| PC(38:3) | 2.463355 | 0.060377 | 2.103478 | 0.061013 | -8.77E-01 | 7.26E-05 |
| PC(38:4) | 3.909531 | 0.104821 | 3.57073 | 0.105925 | -4.76E-01 | 2.66E-02 |
| PC(38:4e) | 0.98684 | 0.033373 | 0.977701 | 0.033725 | -4.03E-02 | 8.49E-01 |
| PC(38:5) | 1.99513 | 0.069088 | 1.490765 | 0.069816 | -1.07E+00 | 1.90E-06 |
| PC(38:6) | 3.795809 | 0.102627 | 3.070746 | 0.103708 | -1.04E+00 | 3.70E-06 |
| PC(38:6e) | 0.284947 | 0.013655 | 0.24976 | 0.013799 | -3.79E-01 | 7.56E-02 |
| PC(38:8) | 0.12395 | 0.00459 | 0.09305 | 0.004638 | -9.91E-01 | 9.34E-06 |
| PC(39:2) | 0.024933 | 0.001546 | 0.028411 | 0.001562 | 3.31E-01 | 1.20E-01 |
| PC(39:3) | 0.145809 | 0.004575 | 0.112982 | 0.004623 | -1.06E+00 | 2.71E-06 |
| PC(39:4) | 0.259412 | 0.008437 | 0.214328 | 0.008526 | -7.86E-01 | 3.36E-04 |
| PC(39:5) | 0.335761 | 0.013468 | 0.260121 | 0.01361 | -8.27E-01 | 1.73E-04 |
| PC(39:6) | 0.252957 | 0.015913 | 0.165757 | 0.016081 | -8.07E-01 | 2.41E-04 |
| PC(39:7) | 0.073526 | 0.005384 | 0.051798 | 0.005441 | -5.94E-01 | 5.98E-03 |
| PC(40:1) | 0.059633 | 0.003218 | 0.052099 | 0.003252 | -3.44E-01 | 1.06E-01 |
| PC(40:2) | 0.122546 | 0.004746 | 0.109217 | 0.004796 | -4.13E-01 | 5.31E-02 |
| PC(40:2e) | 0.093913 | 0.006324 | 0.092618 | 0.006391 | -3.03E-02 | 8.87E-01 |
| PC(40:3) | 0.096523 | 0.006863 | 0.121309 | 0.006935 | 5.32E-01 | 1.35E-02 |
| PC(40:4) | 0.632689 | 0.02908 | 0.635666 | 0.029387 | 1.51E-02 | 9.43E-01 |
| PC(40:5) | 0.924742 | 0.026695 | 0.824942 | 0.026976 | -5.50E-01 | 1.06E-02 |
| PC(40:5e) | 0.527373 | 0.01631 | 0.517658 | 0.016482 | -8.77E-02 | 6.79E-01 |
| PC(40:6) | 2.07241 | 0.057536 | 1.556652 | 0.058142 | -1.32E+00 | 1.30E-08 |
| PC(40:6e) | 0.321213 | 0.013297 | 0.324511 | 0.013437 | 3.65E-02 | 8.63E-01 |
| PC(40:7) | 0.997433 | 0.038845 | 0.626358 | 0.039254 | -1.41E+00 | 2.00E-09 |
| PC(40:8) | 0.440858 | 0.009855 | 0.357667 | 0.009959 | -1.24E+00 | 6.50E-08 |
| PC(40:9) | 0.086032 | 0.003471 | 0.057127 | 0.003507 | -1.23E+00 | 9.10E-08 |
| PC(41:5) | 0.023287 | 0.00121 | 0.025946 | 0.001222 | 3.24E-01 | 1.29E-01 |
| PC(41:6) | 0.070347 | 0.004584 | 0.050808 | 0.004632 | -6.27E-01 | 3.77E-03 |
| PC(41:7) | 0.04926 | 0.002061 | 0.041013 | 0.002083 | -5.89E-01 | 6.41E-03 |
| PC(42:10) | 0.183112 | 0.004481 | 0.146222 | 0.004528 | -1.21E+00 | 1.22E-07 |
| PC(42:2) | 0.031255 | 0.002555 | 0.021426 | 0.002582 | -5.66E-01 | 8.65E-03 |
| PC(42:2e) | 0.016363 | 0.000903 | 0.0159 | 0.000912 | -7.50E-02 | 7.22E-01 |
| PC(42:3) | 0.06529 | 0.003031 | 0.063087 | 0.003063 | -1.07E-01 | 6.13E-01 |
| PC(42:4) | 0.100759 | 0.003329 | 0.098491 | 0.003364 | -1.00E-01 | 6.36E-01 |
| PC(42:5) | 0.084015 | 0.005719 | 0.076007 | 0.005779 | -2.06E-01 | 3.31E-01 |
| PC(42:6) | 0.275691 | 0.007215 | 0.275876 | 0.007292 | 3.67E-03 | 9.86E-01 |
| PC(42:6e) | 0.304342 | 0.010621 | 0.281917 | 0.010732 | -3.11E-01 | 1.44E-01 |
| PC(42:7) | 0.205557 | 0.007341 | 0.14686 | 0.007418 | -1.18E+00 | 2.48E-07 |
| PC(42:8) | 0.17593 | 0.004979 | 0.164078 | 0.005031 | -3.50E-01 | 1.00E-01 |
| PC(42:9) | 0.108933 | 0.003316 | 0.087599 | 0.003351 | -9.47E-01 | 2.10E-05 |
| PC(44:10) | 0.050853 | 0.002714 | 0.048173 | 0.002742 | -1.45E-01 | 4.93E-01 |
| PC(44:11) | 0.075616 | 0.003284 | 0.067387 | 0.003319 | -3.69E-01 | 8.39E-02 |
| PC(44:12) | 0.11221 | 0.005661 | 0.124932 | 0.00572 | 3.31E-01 | 1.20E-01 |
| PC(44:5) | 0.071965 | 0.002785 | 0.059985 | 0.002814 | -6.33E-01 | 3.47E-03 |
| **PE** | | | | | | |
| PE(10:0e_10:4) | 0.53949 | 0.01507 | 0.495209 | 0.015229 | -4.32E-01 | 4.33E-02 |
| PE(16:0_18:1) | 0.483452 | 0.0189 | 0.408003 | 0.019099 | -5.88E-01 | 6.52E-03 |
| PE(16:0_18:2) | 0.634765 | 0.021629 | 0.571982 | 0.021857 | -4.27E-01 | 4.58E-02 |
| PE(16:0_18:3) | 0.064494 | 0.002994 | 0.054442 | 0.003026 | -4.94E-01 | 2.14E-02 |
| PE(16:0_20:4) | 0.649539 | 0.021608 | 0.562585 | 0.021835 | -5.92E-01 | 6.11E-03 |
| PE(16:0_20:5) | 0.141091 | 0.009095 | 0.095325 | 0.009191 | -7.41E-01 | 6.99E-04 |
| PE(16:0_22:6) | 0.714113 | 0.029175 | 0.538347 | 0.029483 | -8.87E-01 | 6.16E-05 |
| PE(16:0p_16:0) | 0.047066 | 0.002886 | 0.038974 | 0.002916 | -4.13E-01 | 5.35E-02 |
| PE(16:0p_18:1) | 0.322265 | 0.010025 | 0.298507 | 0.01013 | -3.49E-01 | 1.02E-01 |
| PE(16:0p_18:2) | 0.478487 | 0.017405 | 0.48469 | 0.017588 | 5.24E-02 | 8.04E-01 |
| PE(16:0p_20:4) | 1.020605 | 0.02871 | 0.945445 | 0.029012 | -3.85E-01 | 7.11E-02 |
| PE(16:0p_20:5) | 0.239445 | 0.012861 | 0.170101 | 0.012996 | -7.94E-01 | 2.99E-04 |
| PE(16:0p_22:5) | 0.938507 | 0.029084 | 0.927472 | 0.029391 | -5.58E-02 | 7.92E-01 |
| PE(16:0p_22:6) | 0.688744 | 0.01898 | 0.595093 | 0.01918 | -7.26E-01 | 8.75E-04 |
| PE(18:0_18:1) | 0.423485 | 0.015641 | 0.343039 | 0.015806 | -7.57E-01 | 5.40E-04 |
| PE(18:0_20:4) | 1.256824 | 0.038552 | 1.048802 | 0.038958 | -7.94E-01 | 2.96E-04 |
| PE(18:0_22:6) | 0.622197 | 0.025974 | 0.445255 | 0.026248 | -1.00E+00 | 7.50E-06 |
| PE(18:0e) | 0.559864 | 0.016841 | 0.490686 | 0.017018 | -6.05E-01 | 5.17E-03 |
| PE(18:0p_18:2) | 0.679921 | 0.024778 | 0.637261 | 0.025039 | -2.53E-01 | 2.33E-01 |
| PE(18:0p_20:3) | 0.160376 | 0.007634 | 0.159346 | 0.007715 | -1.99E-02 | 9.25E-01 |
| PE(18:0p_20:4) | 1.249421 | 0.038496 | 1.143243 | 0.038901 | -4.06E-01 | 5.75E-02 |
| PE(18:0p_20:5) | 0.841211 | 0.027824 | 0.957684 | 0.028117 | 6.16E-01 | 4.41E-03 |
| PE(18:0p_22:4) | 0.353291 | 0.012629 | 0.303879 | 0.012762 | -5.76E-01 | 7.62E-03 |
| PE(18:0p_22:5) | 0.39461 | 0.013207 | 0.35604 | 0.013346 | -4.30E-01 | 4.45E-02 |
| PE(18:0p_22:6) | 0.730266 | 0.020722 | 0.59088 | 0.020941 | -9.90E-01 | 9.50E-06 |
| PE(18:1_18:2) | 0.542126 | 0.026032 | 0.492103 | 0.026306 | -2.83E-01 | 1.83E-01 |
| PE(18:1_20:4) | 0.545861 | 0.023327 | 0.433355 | 0.023573 | -7.10E-01 | 1.12E-03 |
| PE(18:1_22:6) | 0.208148 | 0.014586 | 0.129728 | 0.01474 | -7.91E-01 | 3.10E-04 |
| PE(18:1p_18:2) | 0.535249 | 0.02747 | 0.548884 | 0.027759 | 7.30E-02 | 7.30E-01 |
| PE(18:1p_22:5) | 0.140759 | 0.014547 | 0.124402 | 0.0147 | -1.66E-01 | 4.35E-01 |
| PE(18:1p_22:6) | 0.52346 | 0.015148 | 0.417289 | 0.015308 | -1.03E+00 | 4.34E-06 |
| PE(18:2e) | 0.695624 | 0.022785 | 0.657483 | 0.023025 | -2.46E-01 | 2.46E-01 |
| PE(18:2p_18:2) | 0.087127 | 0.005401 | 0.069048 | 0.005458 | -4.93E-01 | 2.18E-02 |
| PE(20:0p_22:5) | 0.135337 | 0.00823 | 0.108457 | 0.008317 | -4.81E-01 | 2.51E-02 |
| PE(20:0p_22:6) | 0.149114 | 0.004978 | 0.117739 | 0.005031 | -9.28E-01 | 2.98E-05 |
| PE(20:1p_22:6) | 0.187525 | 0.008249 | 0.19778 | 0.008335 | 1.83E-01 | 3.88E-01 |
| PE(20:3e) | 0.08898 | 0.007033 | 0.06975 | 0.007108 | -4.02E-01 | 5.96E-02 |
| **PI** | | | | | | |
| PI(16:0_18:1) | 0.957254 | 0.042841 | 0.998827 | 0.043292 | 1.43E-01 | 5.00E-01 |
| PI(16:0_18:2) | 1.252448 | 0.032963 | 1.286323 | 0.033311 | 1.51E-01 | 4.75E-01 |
| PI(16:0_20:4) | 1.393188 | 0.03275 | 1.336804 | 0.033095 | -2.53E-01 | 2.33E-01 |
| PI(16:0_22:6) | 0.506045 | 0.012302 | 0.434006 | 0.012431 | -8.62E-01 | 9.48E-05 |
| PI(18:0_18:1) | 1.630036 | 0.044053 | 1.567202 | 0.044518 | -2.10E-01 | 3.22E-01 |
| PI(18:0_18:2) | 2.790681 | 0.077879 | 3.000595 | 0.078699 | 3.97E-01 | 6.33E-02 |
| PI(18:0_20:3) | 1.724872 | 0.034641 | 1.537461 | 0.035006 | -7.96E-01 | 2.86E-04 |
| PI(18:0_20:4) | 4.758889 | 0.097906 | 4.45182 | 0.098938 | -4.62E-01 | 3.12E-02 |
| PI(18:0_22:5) | 0.82156 | 0.019707 | 0.736728 | 0.019914 | -6.34E-01 | 3.45E-03 |
| PI(18:0_22:6) | 1.029129 | 0.022036 | 0.835389 | 0.022268 | -1.29E+00 | 2.20E-08 |
| PI(18:1_18:2) | 0.865028 | 0.034971 | 0.948267 | 0.03534 | 3.50E-01 | 1.00E-01 |
| PI(18:1_20:4) | 0.917445 | 0.046797 | 0.914241 | 0.047291 | -1.01E-02 | 9.62E-01 |
| PI(32:1) | 0.413938 | 0.020288 | 0.361881 | 0.020502 | -3.78E-01 | 7.68E-02 |
| PI(34:1) | 0.324579 | 0.016257 | 0.343062 | 0.016429 | 1.67E-01 | 4.30E-01 |
| PI(35:2) | 0.330363 | 0.009314 | 0.320982 | 0.009412 | -1.48E-01 | 4.84E-01 |
| PI(36:3) | 0.256229 | 0.015737 | 0.185343 | 0.015903 | -6.63E-01 | 2.26E-03 |
| **SM** | | | | | | |
| SM(d16:1_20:0) | 0.295586 | 0.020592 | 0.310667 | 0.020809 | 1.08E-01 | 6.11E-01 |
| SM(d17:1_13:0) | 16.08314 | 0.525481 | 18.22602 | 0.531018 | 6.00E-01 | 5.49E-03 |
| SM(d18:1_13:0) | 1.802445 | 0.071011 | 1.783333 | 0.07176 | -3.96E-02 | 8.51E-01 |
| SM(d18:1_18:3) | 2.411311 | 0.101229 | 2.487112 | 0.102296 | 1.10E-01 | 6.03E-01 |
| SM(d18:1_21:0) | 1.906074 | 0.086612 | 1.645775 | 0.087525 | -4.42E-01 | 3.88E-02 |
| SM(d18:1_21:1) | 1.337757 | 0.054929 | 1.235541 | 0.055508 | -2.74E-01 | 1.98E-01 |
| SM(d18:2_24:3) | 1.57894 | 0.074785 | 1.586367 | 0.075573 | 1.46E-02 | 9.45E-01 |
| SM(d28:1) | 1.200193 | 0.055807 | 1.185706 | 0.056395 | -3.82E-02 | 8.57E-01 |
| SM(d30:1) | 0.48682 | 0.031372 | 0.526342 | 0.031703 | 1.85E-01 | 3.82E-01 |
| SM(d31:1) | 2.049446 | 0.097558 | 1.838076 | 0.098587 | -3.19E-01 | 1.34E-01 |
| SM(d32:0) | 2.769136 | 0.130063 | 2.65542 | 0.131434 | -1.29E-01 | 5.43E-01 |
| SM(d32:1) | 14.43498 | 0.615507 | 13.65457 | 0.621993 | -1.87E-01 | 3.79E-01 |
| SM(d32:2) | 4.345521 | 0.188856 | 4.102631 | 0.190847 | -1.89E-01 | 3.72E-01 |
| SM(d32:4) | 1.754707 | 0.11178 | 2.037035 | 0.112958 | 3.72E-01 | 8.15E-02 |
| SM(d33:1) | 12.65806 | 0.55703 | 12.25575 | 0.5629 | -1.06E-01 | 6.16E-01 |
| SM(d33:2) | 1.822243 | 0.08966 | 1.737473 | 0.090605 | -1.39E-01 | 5.11E-01 |
| SM(d34:0) | 11.9084 | 0.492281 | 12.51105 | 0.497468 | 1.80E-01 | 3.95E-01 |
| SM(d34:1) | 50.1362 | 1.865084 | 54.45301 | 1.884738 | 3.41E-01 | 1.10E-01 |
| SM(d34:2) | 18.16218 | 0.719046 | 18.1884 | 0.726623 | 5.37E-03 | 9.80E-01 |
| SM(d34:3) | 1.055395 | 0.063482 | 0.935595 | 0.064151 | -2.78E-01 | 1.91E-01 |
| SM(d34:4) | 1.392095 | 0.067422 | 1.268079 | 0.068132 | -2.71E-01 | 2.03E-01 |
| SM(d34:5) | 0.566368 | 0.030398 | 0.502004 | 0.030719 | -3.12E-01 | 1.43E-01 |
| SM(d35:0) | 1.825346 | 0.09989 | 1.778765 | 0.100943 | -6.86E-02 | 7.46E-01 |
| SM(d35:1) | 5.218311 | 0.303354 | 5.293592 | 0.30655 | 3.65E-02 | 8.63E-01 |
| SM(d35:2) | 3.877199 | 0.175755 | 3.642415 | 0.177607 | -1.97E-01 | 3.54E-01 |
| SM(d35:4) | 1.031907 | 0.046827 | 1.025877 | 0.04732 | -1.90E-02 | 9.29E-01 |
| SM(d36:0) | 5.361553 | 0.261954 | 5.977871 | 0.264714 | 3.46E-01 | 1.04E-01 |
| SM(d36:1) | 26.21305 | 1.014757 | 25.48465 | 1.02545 | -1.06E-01 | 6.18E-01 |
| SM(d36:2) | 19.59465 | 0.822383 | 20.05889 | 0.831049 | 8.31E-02 | 6.95E-01 |
| SM(d36:3) | 4.892069 | 0.221349 | 4.886213 | 0.223681 | -3.90E-03 | 9.85E-01 |
| SM(d37:1) | 2.207178 | 0.179439 | 2.527765 | 0.18133 | 2.63E-01 | 2.16E-01 |
| SM(d37:2) | 3.095283 | 0.152922 | 2.792539 | 0.154534 | -2.91E-01 | 1.71E-01 |
| SM(d38:0) | 3.835825 | 0.176309 | 3.691447 | 0.178167 | -1.21E-01 | 5.69E-01 |
| SM(d38:1) | 20.65792 | 0.808613 | 19.20468 | 0.817134 | -2.65E-01 | 2.13E-01 |
| SM(d38:2) | 13.11343 | 0.498813 | 12.93494 | 0.50407 | -5.27E-02 | 8.03E-01 |
| SM(d38:3) | 3.032981 | 0.19563 | 3.448125 | 0.197692 | 3.12E-01 | 1.42E-01 |
| SM(d39:1) | 12.20295 | 0.522437 | 11.06393 | 0.527942 | -3.21E-01 | 1.32E-01 |
| SM(d39:2) | 5.304985 | 0.240968 | 5.061746 | 0.243507 | -1.49E-01 | 4.83E-01 |
| SM(d40:1) | 26.70497 | 1.033456 | 26.0822 | 1.044346 | -8.87E-02 | 6.75E-01 |
| SM(d40:2) | 23.27616 | 0.905171 | 23.49571 | 0.91471 | 3.57E-02 | 8.66E-01 |
| SM(d40:3) | 7.295615 | 0.315199 | 7.291913 | 0.31852 | -1.73E-03 | 9.93E-01 |
| SM(d40:4) | 2.206031 | 0.111403 | 2.450111 | 0.112577 | 3.22E-01 | 1.30E-01 |
| SM(d41:0) | 1.161129 | 0.099443 | 1.174988 | 0.100491 | 2.05E-02 | 9.23E-01 |
| SM(d41:1) | 16.15173 | 0.65476 | 15.1788 | 0.66166 | -2.19E-01 | 3.03E-01 |
| SM(d41:2) | 7.234794 | 0.486234 | 7.096586 | 0.491358 | -4.18E-02 | 8.43E-01 |
| SM(d41:3) | 4.699242 | 0.243513 | 4.652907 | 0.246079 | -2.80E-02 | 8.95E-01 |
| SM(d42:1) | 20.38739 | 0.948597 | 19.80587 | 0.958593 | -9.02E-02 | 6.70E-01 |
| SM(d42:2) | 28.80003 | 1.306383 | 28.06772 | 1.320149 | -8.25E-02 | 6.97E-01 |
| SM(d42:3) | 25.73467 | 1.000555 | 25.8268 | 1.011099 | 1.36E-02 | 9.49E-01 |
| SM(d42:4) | 8.736626 | 0.345257 | 9.496637 | 0.348895 | 3.24E-01 | 1.28E-01 |
| SM(d42:5) | 2.162551 | 0.161707 | 3.043105 | 0.163411 | 8.02E-01 | 2.63E-04 |
| SM(d42:6) | 0.927958 | 0.070906 | 1.187304 | 0.071653 | 5.38E-01 | 1.24E-02 |
| SM(d43:1) | 4.510041 | 0.211424 | 4.100792 | 0.213652 | -2.85E-01 | 1.80E-01 |
| SM(d43:2) | 6.951314 | 0.410773 | 6.096474 | 0.415102 | -3.06E-01 | 1.50E-01 |
| SM(d43:3) | 3.287074 | 0.18832 | 3.118075 | 0.190305 | -1.32E-01 | 5.33E-01 |
| SM(d43:4) | 2.169575 | 0.086869 | 2.100986 | 0.087784 | -1.16E-01 | 5.83E-01 |
| SM(d44:1) | 0.827872 | 0.07281 | 0.625162 | 0.073577 | -4.10E-01 | 5.52E-02 |
| SM(d44:2) | 2.140288 | 0.08963 | 2.123712 | 0.090575 | -2.72E-02 | 8.98E-01 |
| SM(d44:3) | 2.779827 | 0.120146 | 2.810198 | 0.121412 | 3.72E-02 | 8.60E-01 |
| SM(d44:4) | 2.849447 | 0.109711 | 2.792041 | 0.110867 | -7.70E-02 | 7.16E-01 |
| SM(d44:5) | 1.61848 | 0.138097 | 1.799103 | 0.139553 | 1.93E-01 | 3.64E-01 |
| SM(d44:6) | 1.736955 | 0.071514 | 1.989925 | 0.072268 | 5.21E-01 | 1.55E-02 |
| SM(t18:0_16:1) | 0.730961 | 0.049183 | 0.707183 | 0.049701 | -7.12E-02 | 7.37E-01 |
| SM(t18:0_23:1) | 1.345992 | 0.077812 | 1.489733 | 0.078632 | 2.72E-01 | 2.01E-01 |
| SM(t18:0_24:2) | 1.886048 | 0.101957 | 1.902884 | 0.103031 | 2.43E-02 | 9.09E-01 |
| SM(t32:1) | 0.894241 | 0.042859 | 0.869655 | 0.043311 | -8.45E-02 | 6.90E-01 |
| SM(t34:0) | 2.229325 | 0.091798 | 2.222593 | 0.092765 | -1.08E-02 | 9.59E-01 |
| SM(t34:1) | 3.366205 | 0.146953 | 3.358656 | 0.148501 | -7.56E-03 | 9.71E-01 |
| SM(t34:2) | 1.519317 | 0.065851 | 1.54917 | 0.066545 | 6.67E-02 | 7.53E-01 |
| SM(t36:1) | 0.915317 | 0.040164 | 0.917581 | 0.040588 | 8.28E-03 | 9.69E-01 |
| SM(t36:2) | 0.931872 | 0.051372 | 0.933268 | 0.051913 | 4.01E-03 | 9.85E-01 |
| SM(t39:5) | 3.538721 | 0.162417 | 3.635474 | 0.164128 | 8.77E-02 | 6.79E-01 |
| SM(t40:0) | 1.541165 | 0.06587 | 1.512446 | 0.066564 | -6.42E-02 | 7.62E-01 |
| SM(t40:1) | 1.588844 | 0.073349 | 1.668332 | 0.074122 | 1.60E-01 | 4.52E-01 |
| SM(t40:7) | 3.994719 | 0.162269 | 3.722477 | 0.163979 | -2.47E-01 | 2.45E-01 |
| SM(t42:1) | 3.211708 | 0.140942 | 3.22369 | 0.142428 | 1.25E-02 | 9.53E-01 |
| SM(t42:2) | 0.976884 | 0.063111 | 1.030586 | 0.063776 | 1.25E-01 | 5.54E-01 |
| SM(t42:3) | 1.59203 | 0.080835 | 1.606511 | 0.081687 | 2.64E-02 | 9.01E-01 |
| SM(t42:4) | 0.581773 | 0.034756 | 0.600657 | 0.035123 | 8.00E-02 | 7.06E-01 |
| **TG** | | | | | | |
| TG(10:0_12:0_12:0) | 0.04766 | 0.003423 | 0.030481 | 0.003459 | -7.39E-01 | 7.20E-04 |
| TG(10:0_18:1_18:1) | 0.160309 | 0.0095 | 0.122348 | 0.0096 | -5.88E-01 | 6.47E-03 |
| TG(10:0_18:2_18:2) | 0.101186 | 0.008003 | 0.099042 | 0.008087 | -3.94E-02 | 8.52E-01 |
| TG(10:0_18:2_18:3) | 0.039956 | 0.01059 | 0.106868 | 0.010701 | 9.30E-01 | 2.85E-05 |
| TG(11:0_18:0_18:0) | 0.067217 | 0.004186 | 0.049743 | 0.00423 | -6.14E-01 | 4.51E-03 |
| TG(12:0_12:0_12:0) | 0.076769 | 0.005932 | 0.040963 | 0.005994 | -8.89E-01 | 5.97E-05 |
| TG(12:0_12:0_14:0) | 0.109865 | 0.009616 | 0.109335 | 0.009717 | -8.11E-03 | 9.69E-01 |
| TG(12:0_12:0_18:2) | 0.125533 | 0.009007 | 0.090174 | 0.009102 | -5.78E-01 | 7.43E-03 |
| TG(12:0_14:0_14:0) | 0.005136 | 0.000528 | 0.006625 | 0.000533 | 4.15E-01 | 5.22E-02 |
| TG(12:0_14:0_14:3) | 0.024148 | 0.003664 | 0.035242 | 0.003702 | 4.46E-01 | 3.74E-02 |
| TG(12:0_14:0_18:3) | 0.069043 | 0.004747 | 0.050011 | 0.004797 | -5.90E-01 | 6.30E-03 |
| TG(12:0_14:0_22:6) | 0.04175 | 0.00213 | 0.033952 | 0.002152 | -5.39E-01 | 1.23E-02 |
| TG(12:0_17:1_18:2) | 0.077961 | 0.003866 | 0.055234 | 0.003907 | -8.65E-01 | 8.96E-05 |
| TG(12:0_18:2_18:2) | 0.267339 | 0.013695 | 0.222323 | 0.013839 | -4.84E-01 | 2.42E-02 |
| TG(12:0_18:2_18:3) | 0.100147 | 0.009431 | 0.107605 | 0.009531 | 1.16E-01 | 5.83E-01 |
| TG(12:0_18:2_20:5) | 0.027234 | 0.001886 | 0.018252 | 0.001905 | -7.01E-01 | 1.28E-03 |
| TG(12:0_18:2_22:6) | 0.04572 | 0.002689 | 0.027027 | 0.002718 | -1.02E+00 | 5.11E-06 |
| TG(12:0_18:3_18:3) | 0.044304 | 0.002422 | 0.035293 | 0.002447 | -5.48E-01 | 1.10E-02 |
| TG(14:0_14:0_22:6) | 0.08001 | 0.003756 | 0.051439 | 0.003796 | -1.12E+00 | 7.84E-07 |
| TG(14:0_18:2_18:2) | 0.970424 | 0.033423 | 0.897645 | 0.033775 | -3.21E-01 | 1.32E-01 |
| TG(14:0_18:2_18:3) | 0.168773 | 0.008231 | 0.155423 | 0.008318 | -2.39E-01 | 2.61E-01 |
| TG(14:0_18:2_20:5) | 0.128607 | 0.003645 | 0.146482 | 0.003683 | 7.22E-01 | 9.37E-04 |
| TG(14:0_18:3_20:5) | 0.060555 | 0.002436 | 0.061843 | 0.002462 | 7.79E-02 | 7.13E-01 |
| TG(14:0_18:3_22:6) | 0.037332 | 0.002446 | 0.022414 | 0.002472 | -8.98E-01 | 5.07E-05 |
| TG(15:0_12:0_16:0) | 0.073242 | 0.004593 | 0.038756 | 0.004641 | -1.11E+00 | 1.04E-06 |
| TG(15:0_12:0_18:1) | 0.076406 | 0.004392 | 0.041953 | 0.004438 | -1.15E+00 | 3.89E-07 |
| TG(15:0_12:0_18:2) | 0.084928 | 0.00456 | 0.051519 | 0.004608 | -1.08E+00 | 1.76E-06 |
| TG(15:0_12:0_18:3) | 0.026219 | 0.001797 | 0.016552 | 0.001816 | -7.92E-01 | 3.09E-04 |
| TG(15:0_14:0_16:1) | 0.137047 | 0.006863 | 0.090141 | 0.006936 | -1.01E+00 | 7.05E-06 |
| TG(15:0_14:0_18:1) | 0.145292 | 0.006127 | 0.116924 | 0.006192 | -6.82E-01 | 1.72E-03 |
| TG(15:0_14:0_18:2) | 0.24899 | 0.009172 | 0.184376 | 0.009269 | -1.04E+00 | 3.92E-06 |
| TG(15:0_14:0_18:3) | 0.059285 | 0.003086 | 0.046109 | 0.003118 | -6.29E-01 | 3.71E-03 |
| TG(15:0_14:0_20:5) | 0.026657 | 0.002099 | 0.012916 | 0.002121 | -9.64E-01 | 1.55E-05 |
| TG(15:0_14:0_22:6) | 0.030235 | 0.00172 | 0.012484 | 0.001738 | -1.52E+00 | <1E-10 |
| TG(15:0_15:0_15:0) | 0.10894 | 0.005619 | 0.085059 | 0.005679 | -6.26E-01 | 3.87E-03 |
| TG(15:0_16:0_16:0) | 0.270535 | 0.009856 | 0.241049 | 0.00996 | -4.40E-01 | 3.97E-02 |
| TG(15:0_16:0_18:1) | 0.646548 | 0.012281 | 0.602523 | 0.01241 | -5.28E-01 | 1.42E-02 |
| TG(15:0_16:0_18:2) | 0.597595 | 0.007852 | 0.555433 | 0.007934 | -7.90E-01 | 3.15E-04 |
| TG(15:0_16:0_18:3) | 0.125903 | 0.007333 | 0.103731 | 0.00741 | -4.45E-01 | 3.77E-02 |
| TG(15:0_16:0_20:4) | 0.165341 | 0.006929 | 0.168599 | 0.007002 | 6.93E-02 | 7.44E-01 |
| TG(15:0_16:0_20:5) | 0.063954 | 0.00295 | 0.047504 | 0.002981 | -8.21E-01 | 1.90E-04 |
| TG(15:0_16:1_16:1) | 0.06798 | 0.004137 | 0.045816 | 0.004181 | -7.88E-01 | 3.25E-04 |
| TG(15:0_16:1_18:2) | 0.302211 | 0.00777 | 0.255892 | 0.007852 | -8.77E-01 | 7.24E-05 |
| TG(15:0_16:1_18:3) | 0.055808 | 0.002882 | 0.036403 | 0.002912 | -9.91E-01 | 9.32E-06 |
| TG(15:0_18:1_18:2) | 0.815514 | 0.015442 | 0.81389 | 0.015605 | -1.54E-02 | 9.42E-01 |
| TG(15:0_18:1_18:3) | 0.037933 | 0.001637 | 0.037582 | 0.001654 | -3.15E-02 | 8.82E-01 |
| TG(15:0_18:1_20:4) | 0.018317 | 0.001401 | 0.012757 | 0.001416 | -5.84E-01 | 6.84E-03 |
| TG(15:0_18:1_20:5) | 0.089631 | 0.004742 | 0.050497 | 0.004792 | -1.21E+00 | 1.15E-07 |
| TG(15:0_18:1_22:6) | 0.143998 | 0.005718 | 0.101146 | 0.005778 | -1.10E+00 | 1.09E-06 |
| TG(15:0_18:2_18:2) | 0.428139 | 0.013667 | 0.379572 | 0.013811 | -5.23E-01 | 1.50E-02 |
| TG(15:0_18:2_18:3) | 0.099473 | 0.006963 | 0.087041 | 0.007037 | -2.63E-01 | 2.16E-01 |
| TG(15:0_18:2_22:6) | 0.082762 | 0.004055 | 0.046226 | 0.004098 | -1.33E+00 | 1.10E-08 |
| TG(15:0_9:0_9:0) | 0.20255 | 0.018465 | 0.278459 | 0.01866 | 6.05E-01 | 5.14E-03 |
| TG(16:0_10:0_18:2) | 0.224781 | 0.013171 | 0.164295 | 0.01331 | -6.76E-01 | 1.87E-03 |
| TG(16:0_10:1_16:0) | 0.028637 | 0.002601 | 0.024765 | 0.002629 | -2.19E-01 | 3.02E-01 |
| TG(16:0_10:1_18:1) | 0.053782 | 0.004105 | 0.043067 | 0.004148 | -3.84E-01 | 7.19E-02 |
| TG(16:0_11:1_16:0) | 0.024508 | 0.001867 | 0.018471 | 0.001886 | -4.76E-01 | 2.64E-02 |
| TG(16:0_11:1_18:1) | 0.056073 | 0.002176 | 0.047517 | 0.002199 | -5.79E-01 | 7.33E-03 |
| TG(16:0_11:1_18:2) | 0.023256 | 0.001607 | 0.021108 | 0.001624 | -1.97E-01 | 3.54E-01 |
| TG(16:0_12:0_14:0) | 0.00669 | 0.000356 | 0.006261 | 0.00036 | -1.78E-01 | 4.03E-01 |
| TG(16:0_12:0_18:1) | 0.799925 | 0.03399 | 0.652807 | 0.034349 | -6.37E-01 | 3.29E-03 |
| TG(16:0_12:0_18:3) | 0.146561 | 0.010264 | 0.095558 | 0.010372 | -7.31E-01 | 8.07E-04 |
| TG(16:0_12:0_20:4) | 0.086951 | 0.002871 | 0.08847 | 0.002902 | 7.79E-02 | 7.13E-01 |
| TG(16:0_12:1_18:1) | 0.096614 | 0.006274 | 0.097908 | 0.00634 | 3.03E-02 | 8.86E-01 |
| TG(16:0_14:0_14:0) | 0.424405 | 0.02388 | 0.310874 | 0.024131 | -7.00E-01 | 1.31E-03 |
| TG(16:0_14:0_16:0) | 0.526914 | 0.024891 | 0.486936 | 0.025153 | -2.36E-01 | 2.66E-01 |
| TG(16:0_14:0_16:1) | 0.18465 | 0.014969 | 0.166182 | 0.015127 | -1.82E-01 | 3.92E-01 |
| TG(16:0_14:0_18:1) | 1.260473 | 0.03633 | 1.233109 | 0.036713 | -1.11E-01 | 6.01E-01 |
| TG(16:0_14:0_18:2) | 0.109861 | 0.007775 | 0.127821 | 0.007857 | 3.40E-01 | 1.11E-01 |
| TG(16:0_14:0_18:3) | 0.090014 | 0.005381 | 0.081492 | 0.005437 | -2.33E-01 | 2.72E-01 |
| TG(16:0_14:0_20:5) | 0.145807 | 0.00825 | 0.089425 | 0.008337 | -1.01E+00 | 7.06E-06 |
| TG(16:0_14:1_18:1) | 1.227142 | 0.036185 | 1.150045 | 0.036566 | -3.14E-01 | 1.41E-01 |
| TG(16:0_16:0_16:0) | 0.59012 | 0.025657 | 0.673433 | 0.025928 | 4.78E-01 | 2.59E-02 |
| TG(16:0_16:0_17:0) | 0.295254 | 0.010328 | 0.265665 | 0.010437 | -4.22E-01 | 4.86E-02 |
| TG(16:0_16:0_18:1) | 1.6793 | 0.042907 | 1.978033 | 0.043359 | 1.02E+00 | 4.94E-06 |
| TG(16:0_16:0_18:2) | 0.578738 | 0.023328 | 0.638369 | 0.023574 | 3.76E-01 | 7.79E-02 |
| TG(16:0_16:0_18:3) | 0.668237 | 0.045145 | 0.763241 | 0.045621 | 3.10E-01 | 1.46E-01 |
| TG(16:0_16:0_23:0) | 0.26279 | 0.012239 | 0.306868 | 0.012368 | 5.30E-01 | 1.38E-02 |
| TG(16:0_16:0_24:0) | 0.123046 | 0.006138 | 0.124391 | 0.006202 | 3.21E-02 | 8.79E-01 |
| TG(16:0_16:1_16:1) | 1.342682 | 0.035329 | 1.255706 | 0.035701 | -3.62E-01 | 8.93E-02 |
| TG(16:0_16:1_18:1) | 2.237965 | 0.037066 | 2.459005 | 0.037456 | 8.78E-01 | 7.21E-05 |
| TG(16:0_16:1_18:3) | 0.349807 | 0.016433 | 0.292608 | 0.016606 | -5.12E-01 | 1.72E-02 |
| TG(16:0_16:1_20:5) | 0.160546 | 0.004998 | 0.202641 | 0.005051 | 1.24E+00 | 6.80E-08 |
| TG(16:0_17:0_18:1) | 0.661512 | 0.012636 | 0.620536 | 0.01277 | -4.77E-01 | 2.61E-02 |
| TG(16:0_17:1_18:1) | 0.258065 | 0.01167 | 0.197397 | 0.011793 | -7.65E-01 | 4.74E-04 |
| TG(16:0_17:1_18:2) | 0.22695 | 0.020537 | 0.189775 | 0.020753 | -2.66E-01 | 2.10E-01 |
| TG(16:0_18:1_18:1) | 2.925707 | 0.070485 | 3.525688 | 0.071228 | 1.25E+00 | 5.20E-08 |
| TG(16:0_18:1_18:2) | 3.299132 | 0.087264 | 3.861078 | 0.088184 | 9.48E-01 | 2.07E-05 |
| TG(16:0_18:1_18:3) | 2.693846 | 0.086181 | 3.138436 | 0.087089 | 7.59E-01 | 5.20E-04 |
| TG(16:0_18:1_19:0) | 0.398184 | 0.005295 | 0.395588 | 0.005351 | -7.23E-02 | 7.33E-01 |
| TG(16:0_18:1_20:4) | 0.024334 | 0.001421 | 0.026545 | 0.001436 | 2.29E-01 | 2.81E-01 |
| TG(16:0_18:1_20:5) | 0.136283 | 0.006526 | 0.188277 | 0.006595 | 1.17E+00 | 2.70E-07 |
| TG(16:0_18:1_21:0) | 0.190009 | 0.005435 | 0.179977 | 0.005492 | -2.72E-01 | 2.01E-01 |
| TG(16:0_18:1_22:6) | 0.844942 | 0.028965 | 0.730262 | 0.02927 | -5.83E-01 | 6.95E-03 |
| TG(16:0_18:1_23:0) | 0.543903 | 0.01374 | 0.643522 | 0.013885 | 1.07E+00 | 2.19E-06 |
| TG(16:0_18:1_23:1) | 0.154185 | 0.008349 | 0.166018 | 0.008437 | 2.09E-01 | 3.25E-01 |
| TG(16:0_18:1_24:0) | 0.197016 | 0.012788 | 0.199676 | 0.012923 | 3.06E-02 | 8.85E-01 |
| TG(16:0_18:2_18:2) | 0.543459 | 0.021807 | 0.569655 | 0.022036 | 1.77E-01 | 4.04E-01 |
| TG(16:0_18:2_18:3) | 1.303862 | 0.062225 | 1.362149 | 0.062881 | 1.38E-01 | 5.15E-01 |
| TG(16:0_18:2_20:5) | 0.209425 | 0.008085 | 0.27378 | 0.00817 | 1.17E+00 | 2.76E-07 |
| TG(16:0_18:2_22:6) | 0.745976 | 0.031155 | 0.570442 | 0.031483 | -8.29E-01 | 1.65E-04 |
| TG(16:0_18:3_18:3) | 0.03047 | 0.001833 | 0.040294 | 0.001852 | 7.89E-01 | 3.23E-04 |
| TG(16:0_18:3_20:5) | 0.066984 | 0.003075 | 0.067917 | 0.003108 | 4.45E-02 | 8.33E-01 |
| TG(16:0_20:4_22:6) | 0.16753 | 0.007958 | 0.114338 | 0.008042 | -9.84E-01 | 1.07E-05 |
| TG(16:0_22:1_22:6) | 0.05646 | 0.004024 | 0.053874 | 0.004067 | -9.47E-02 | 6.55E-01 |
| TG(16:0_22:6_22:6) | 0.098644 | 0.006266 | 0.041831 | 0.006332 | -1.33E+00 | 9.00E-09 |
| TG(16:0_9:0_18:1) | 0.061353 | 0.003597 | 0.041797 | 0.003635 | -8.00E-01 | 2.68E-04 |
| TG(16:0e_16:0_16:0) | 0.06445 | 0.002865 | 0.075467 | 0.002895 | 5.66E-01 | 8.68E-03 |
| TG(16:0e_16:0_18:1) | 0.135821 | 0.006583 | 0.173356 | 0.006652 | 8.39E-01 | 1.40E-04 |
| TG(16:0e_16:0_18:2) | 0.083767 | 0.004826 | 0.114901 | 0.004877 | 9.49E-01 | 2.00E-05 |
| TG(16:1_14:0_18:1) | 1.371144 | 0.034935 | 1.271544 | 0.035303 | -4.20E-01 | 4.97E-02 |
| TG(16:1_16:1_18:1) | 1.758332 | 0.040205 | 1.856612 | 0.040629 | 3.60E-01 | 9.16E-02 |
| TG(16:1_18:1_18:1) | 0.995228 | 0.048568 | 1.225848 | 0.049079 | 6.99E-01 | 1.33E-03 |
| TG(16:1_18:2_18:2) | 0.156283 | 0.007788 | 0.140502 | 0.00787 | -2.98E-01 | 1.61E-01 |
| TG(16:1_18:2_18:3) | 0.123915 | 0.00809 | 0.092887 | 0.008175 | -5.65E-01 | 8.85E-03 |
| TG(16:1_18:2_20:5) | 0.156339 | 0.007585 | 0.183691 | 0.007665 | 5.31E-01 | 1.36E-02 |
| TG(16:1_18:3_18:3) | 0.023156 | 0.001412 | 0.021796 | 0.001427 | -1.42E-01 | 5.03E-01 |
| TG(16:1_18:3_20:5) | 0.035271 | 0.002833 | 0.033667 | 0.002863 | -8.31E-02 | 6.94E-01 |
| TG(17:0_18:1_18:1) | 0.81816 | 0.010013 | 0.804176 | 0.010118 | -2.06E-01 | 3.33E-01 |
| TG(17:0_18:1_18:3) | 0.547815 | 0.020691 | 0.587939 | 0.020909 | 2.85E-01 | 1.80E-01 |
| TG(17:0_18:1_22:4) | 0.057452 | 0.002299 | 0.06298 | 0.002324 | 3.54E-01 | 9.70E-02 |
| TG(17:0_18:1_22:6) | 0.121271 | 0.006336 | 0.090468 | 0.006403 | -7.16E-01 | 1.03E-03 |
| TG(17:0_18:2_20:4) | 0.213963 | 0.009184 | 0.208986 | 0.009281 | -7.98E-02 | 7.06E-01 |
| TG(17:0_20:5_22:6) | 0.02186 | 0.001437 | 0.016793 | 0.001452 | -5.19E-01 | 1.58E-02 |
| TG(18:0_10:0_18:0) | 0.32515 | 0.01571 | 0.266699 | 0.015876 | -5.48E-01 | 1.10E-02 |
| TG(18:0_12:1_16:0) | 0.269804 | 0.015319 | 0.187692 | 0.015481 | -7.89E-01 | 3.23E-04 |
| TG(18:0_16:0_16:0) | 0.647609 | 0.027927 | 0.729422 | 0.028221 | 4.31E-01 | 4.39E-02 |
| TG(18:0_16:0_17:0) | 0.235791 | 0.00927 | 0.212534 | 0.009368 | -3.69E-01 | 8.35E-02 |
| TG(18:0_16:0_17:1) | 0.076475 | 0.003355 | 0.06054 | 0.003391 | -6.99E-01 | 1.33E-03 |
| TG(18:0_16:0_18:0) | 0.461903 | 0.024243 | 0.509517 | 0.024499 | 2.89E-01 | 1.74E-01 |
| TG(18:0_16:0_18:1) | 1.458408 | 0.036519 | 1.653581 | 0.036903 | 7.87E-01 | 3.35E-04 |
| TG(18:0_16:0_24:0) | 0.073814 | 0.003848 | 0.074769 | 0.003889 | 3.63E-02 | 8.63E-01 |
| TG(18:0_16:1_17:0) | 0.421687 | 0.005357 | 0.423056 | 0.005414 | 3.76E-02 | 8.59E-01 |
| TG(18:0_17:0_18:0) | 0.120809 | 0.005008 | 0.115727 | 0.00506 | -1.49E-01 | 4.81E-01 |
| TG(18:0_17:0_18:1) | 0.393095 | 0.011817 | 0.353396 | 0.011942 | -4.94E-01 | 2.13E-02 |
| TG(18:0_17:0_20:4) | 0.064541 | 0.00368 | 0.065324 | 0.003719 | 3.12E-02 | 8.82E-01 |
| TG(18:0_18:0_18:0) | 0.203362 | 0.011623 | 0.216069 | 0.011746 | 1.61E-01 | 4.48E-01 |
| TG(18:0_18:0_18:1) | 0.765782 | 0.038749 | 0.840721 | 0.039158 | 2.85E-01 | 1.81E-01 |
| TG(18:0_18:1_18:1) | 1.516099 | 0.043678 | 1.810253 | 0.044138 | 9.91E-01 | 9.28E-06 |
| TG(18:0_18:1_20:0) | 0.286324 | 0.018319 | 0.293044 | 0.018512 | 5.40E-02 | 7.99E-01 |
| TG(18:0_18:1_20:3) | 0.041215 | 0.001908 | 0.048676 | 0.001928 | 5.76E-01 | 7.65E-03 |
| TG(18:0_18:1_20:4) | 0.077932 | 0.002921 | 0.102935 | 0.002952 | 1.26E+00 | 4.50E-08 |
| TG(18:0_18:1_22:4) | 0.256738 | 0.007988 | 0.283302 | 0.008072 | 4.89E-01 | 2.26E-02 |
| TG(18:0_18:1_22:6) | 0.446977 | 0.015052 | 0.539644 | 0.015211 | 9.06E-01 | 4.37E-05 |
| TG(18:0_18:1_24:0) | 0.108107 | 0.006234 | 0.105271 | 0.006299 | -6.71E-02 | 7.52E-01 |
| TG(18:0_20:4_22:5) | 0.147238 | 0.007881 | 0.123837 | 0.007964 | -4.37E-01 | 4.12E-02 |
| TG(18:0_20:5_22:6) | 0.070848 | 0.003711 | 0.045363 | 0.003751 | -1.01E+00 | 6.45E-06 |
| TG(18:0_22:6_22:6) | 0.062938 | 0.003028 | 0.076002 | 0.00306 | 6.35E-01 | 3.38E-03 |
| TG(18:0e_16:0_16:0) | 0.103356 | 0.005407 | 0.134085 | 0.005464 | 8.37E-01 | 1.46E-04 |
| TG(18:0e_16:0_18:1) | 0.042071 | 0.002154 | 0.05701 | 0.002177 | 1.02E+00 | 5.34E-06 |
| TG(18:0e_16:0_20:4) | 0.068986 | 0.004048 | 0.096175 | 0.004091 | 9.89E-01 | 9.77E-06 |
| TG(18:0e_18:1_18:1) | 0.180907 | 0.011766 | 0.124928 | 0.01189 | -7.00E-01 | 1.30E-03 |
| TG(18:1_12:0_12:0) | 0.39896 | 0.021315 | 0.265412 | 0.021539 | -9.22E-01 | 3.28E-05 |
| TG(18:1_12:0_14:0) | 0.043757 | 0.003284 | 0.028312 | 0.003319 | -6.92E-01 | 1.47E-03 |
| TG(18:1_12:0_14:3) | 0.377931 | 0.017555 | 0.286532 | 0.01774 | -7.66E-01 | 4.65E-04 |
| TG(18:1_12:0_18:1) | 0.689536 | 0.029097 | 0.529005 | 0.029404 | -8.12E-01 | 2.21E-04 |
| TG(18:1_12:0_18:2) | 0.095221 | 0.00629 | 0.083297 | 0.006356 | -2.79E-01 | 1.89E-01 |
| TG(18:1_12:0_22:6) | 0.164093 | 0.005149 | 0.137178 | 0.005203 | -7.69E-01 | 4.43E-04 |
| TG(18:1_13:0_18:1) | 0.754411 | 0.0233 | 0.815245 | 0.023546 | 3.84E-01 | 7.19E-02 |
| TG(18:1_14:0_18:1) | 0.381168 | 0.014975 | 0.253433 | 0.015133 | -1.26E+00 | 4.90E-08 |
| TG(18:1_14:0_22:6) | 0.337007 | 0.016246 | 0.297253 | 0.016418 | -3.60E-01 | 9.13E-02 |
| TG(18:1_14:1_18:1) | 0.104574 | 0.002796 | 0.108731 | 0.002825 | 2.19E-01 | 3.02E-01 |
| TG(18:1_14:3_18:2) | 0.310459 | 0.01234 | 0.252885 | 0.01247 | -6.87E-01 | 1.60E-03 |
| TG(18:1_17:1_18:1) | 0.586607 | 0.018825 | 0.576807 | 0.019023 | -7.66E-02 | 7.17E-01 |
| TG(18:1_17:1_18:2) | 0.136921 | 0.005792 | 0.116759 | 0.005853 | -5.12E-01 | 1.71E-02 |
| TG(18:1_17:1_20:5) | 0.138494 | 0.004192 | 0.147567 | 0.004236 | 3.18E-01 | 1.35E-01 |
| TG(18:1_17:1_22:1) | 0.085874 | 0.006764 | 0.06396 | 0.006835 | -4.77E-01 | 2.62E-02 |
| TG(18:1_17:1_22:6) | 1.891827 | 0.061486 | 2.313775 | 0.062134 | 1.01E+00 | 6.52E-06 |
| TG(18:1_18:1_18:1) | 2.094555 | 0.078669 | 2.619358 | 0.079498 | 9.82E-01 | 1.11E-05 |
| TG(18:1_18:1_18:2) | 0.344043 | 0.017733 | 0.399069 | 0.01792 | 4.57E-01 | 3.30E-02 |
| TG(18:1_18:1_18:3) | 0.739733 | 0.036441 | 0.963696 | 0.036825 | 9.05E-01 | 4.49E-05 |
| TG(18:1_18:1_20:2) | 0.609525 | 0.034957 | 0.67578 | 0.035326 | 2.79E-01 | 1.89E-01 |
| TG(18:1_18:1_20:3) | 0.492772 | 0.01991 | 0.571566 | 0.02012 | 5.82E-01 | 6.98E-03 |
| TG(18:1_18:1_20:4) | 0.698688 | 0.030822 | 0.965495 | 0.031147 | 1.27E+00 | 3.30E-08 |
| TG(18:1_18:1_21:0) | 0.29542 | 0.017122 | 0.312132 | 0.017303 | 1.44E-01 | 4.98E-01 |
| TG(18:1_18:1_22:0) | 0.285723 | 0.015911 | 0.329117 | 0.016079 | 4.01E-01 | 6.03E-02 |
| TG(18:1_18:1_22:1) | 0.355122 | 0.019834 | 0.347787 | 0.020043 | -5.45E-02 | 7.97E-01 |
| TG(18:1_18:1_22:4) | 0.320812 | 0.012753 | 0.299854 | 0.012887 | -2.42E-01 | 2.55E-01 |
| TG(18:1_18:1_22:5) | 0.538455 | 0.023796 | 0.464106 | 0.024047 | -4.60E-01 | 3.19E-02 |
| TG(18:1_18:1_22:6) | 0.131213 | 0.006249 | 0.130313 | 0.006315 | -2.12E-02 | 9.20E-01 |
| TG(18:1_18:1_23:0) | 0.117316 | 0.005248 | 0.130158 | 0.005303 | 3.60E-01 | 9.12E-02 |
| TG(18:1_18:1_23:1) | 0.175319 | 0.011019 | 0.174639 | 0.011136 | -9.08E-03 | 9.66E-01 |
| TG(18:1_18:1_24:0) | 0.188195 | 0.01071 | 0.195636 | 0.010822 | 1.02E-01 | 6.29E-01 |
| TG(18:1_18:1_24:1) | 1.047826 | 0.032561 | 1.095109 | 0.032905 | 2.14E-01 | 3.14E-01 |
| TG(18:1_18:2_18:2) | 1.10059 | 0.055168 | 1.197393 | 0.05575 | 2.58E-01 | 2.24E-01 |
| TG(18:1_18:2_18:3) | 1.145965 | 0.038524 | 1.211667 | 0.03893 | 2.51E-01 | 2.37E-01 |
| TG(18:1_18:2_20:3) | 0.86917 | 0.034625 | 0.958489 | 0.034989 | 3.80E-01 | 7.53E-02 |
| TG(18:1_18:2_20:4) | 0.136835 | 0.007701 | 0.198853 | 0.007782 | 1.19E+00 | 2.09E-07 |
| TG(18:1_18:2_20:5) | 0.132991 | 0.004179 | 0.145367 | 0.004223 | 4.36E-01 | 4.17E-02 |
| TG(18:1_18:2_21:1) | 0.493004 | 0.023107 | 0.414745 | 0.023351 | -4.99E-01 | 2.03E-02 |
| TG(18:1_18:2_22:6) | 0.107364 | 0.008136 | 0.171467 | 0.008221 | 1.16E+00 | 3.51E-07 |
| TG(18:1_18:2_23:0) | 0.034881 | 0.00149 | 0.04043 | 0.001506 | 5.48E-01 | 1.10E-02 |
| TG(18:1_18:3_18:3) | 0.076836 | 0.002503 | 0.085537 | 0.002529 | 5.12E-01 | 1.73E-02 |
| TG(18:1_18:3_20:4) | 0.233962 | 0.005751 | 0.272458 | 0.005812 | 9.85E-01 | 1.04E-05 |
| TG(18:1_18:3_24:1) | 0.124613 | 0.006347 | 0.096886 | 0.006414 | -6.43E-01 | 3.02E-03 |
| TG(18:1_20:3_22:6) | 0.09022 | 0.00618 | 0.085654 | 0.006246 | -1.09E-01 | 6.08E-01 |
| TG(18:1_20:4_22:0) | 0.51673 | 0.015023 | 0.6372 | 0.015181 | 1.18E+00 | 2.31E-07 |
| TG(18:1_20:4_22:1) | 0.174178 | 0.008709 | 0.132593 | 0.008801 | -7.03E-01 | 1.25E-03 |
| TG(18:1_20:4_22:6) | 0.067594 | 0.003897 | 0.056208 | 0.003938 | -4.30E-01 | 4.45E-02 |
| TG(18:1_20:4_24:0) | 0.025939 | 0.001742 | 0.025113 | 0.00176 | -7.01E-02 | 7.42E-01 |
| TG(18:1_22:1_23:1) | 0.034881 | 0.002741 | 0.023344 | 0.00277 | -6.20E-01 | 4.20E-03 |
| TG(18:1_22:5_22:6) | 0.058365 | 0.003967 | 0.030976 | 0.004009 | -1.02E+00 | 5.82E-06 |
| TG(18:2_14:1_18:2) | 0.286518 | 0.014839 | 0.259481 | 0.014995 | -2.68E-01 | 2.07E-01 |
| TG(18:2_17:1_18:2) | 0.154286 | 0.007244 | 0.148751 | 0.007321 | -1.13E-01 | 5.95E-01 |
| TG(18:2_18:2_18:2) | 0.147545 | 0.012552 | 0.158506 | 0.012684 | 1.29E-01 | 5.44E-01 |
| TG(18:2_18:2_20:4) | 0.459277 | 0.026066 | 0.407423 | 0.02634 | -2.93E-01 | 1.69E-01 |
| TG(18:2_18:2_22:6) | 0.267853 | 0.016302 | 0.230079 | 0.016473 | -3.41E-01 | 1.09E-01 |
| TG(18:2_20:4_22:6) | 0.083533 | 0.005736 | 0.063172 | 0.005797 | -5.22E-01 | 1.51E-02 |
| TG(18:2_22:6_22:6) | 0.039246 | 0.002825 | 0.022392 | 0.002855 | -8.78E-01 | 7.17E-05 |
| TG(18:3_14:1_18:2) | 0.048679 | 0.003731 | 0.037317 | 0.00377 | -4.48E-01 | 3.64E-02 |
| TG(18:3_14:3_18:2) | 0.012667 | 0.001052 | 0.013097 | 0.001063 | 6.02E-02 | 7.76E-01 |
| TG(18:3_17:1_18:2) | 0.129615 | 0.006634 | 0.102278 | 0.006704 | -6.07E-01 | 5.04E-03 |
| TG(18:3_18:2_18:2) | 0.369081 | 0.020709 | 0.313314 | 0.020927 | -3.96E-01 | 6.35E-02 |
| TG(18:3_18:2_18:3) | 0.194763 | 0.009676 | 0.123251 | 0.009778 | -1.09E+00 | 1.46E-06 |
| TG(18:3_18:2_20:4) | 0.140701 | 0.009759 | 0.127627 | 0.009862 | -1.97E-01 | 3.53E-01 |
| TG(18:3_18:2_20:5) | 0.037252 | 0.003627 | 0.029715 | 0.003666 | -3.06E-01 | 1.51E-01 |
| TG(18:3_18:2_22:6) | 0.094692 | 0.007821 | 0.078677 | 0.007904 | -3.01E-01 | 1.57E-01 |
| TG(18:3_18:3_18:3) | 0.016657 | 0.001588 | 0.015487 | 0.001604 | -1.08E-01 | 6.08E-01 |
| TG(18:3_18:3_20:5) | 0.018298 | 0.001931 | 0.022124 | 0.001952 | 2.92E-01 | 1.70E-01 |
| TG(18:4_16:0_18:1) | 0.106435 | 0.003848 | 0.142932 | 0.003888 | 1.40E+00 | 2.00E-09 |
| TG(19:0_18:1_18:1) | 0.338268 | 0.007012 | 0.348427 | 0.007086 | 2.13E-01 | 3.15E-01 |
| TG(19:0_19:0_20:5) | 0.250729 | 0.014755 | 0.308094 | 0.01491 | 5.72E-01 | 8.00E-03 |
| TG(19:1_15:0_17:0) | 0.049064 | 0.00248 | 0.047928 | 0.002506 | -6.77E-02 | 7.50E-01 |
| TG(19:1_16:0_18:1) | 0.105506 | 0.003522 | 0.086831 | 0.003559 | -7.81E-01 | 3.71E-04 |
| TG(19:1_17:1_17:1) | 0.88219 | 0.016915 | 0.913922 | 0.017093 | 2.76E-01 | 1.94E-01 |
| TG(19:1_18:0_18:1) | 0.264557 | 0.006178 | 0.256932 | 0.006243 | -1.82E-01 | 3.92E-01 |
| TG(19:1_18:1_18:1) | 0.380272 | 0.01034 | 0.419148 | 0.010449 | 5.53E-01 | 1.02E-02 |
| TG(19:1_18:1_18:2) | 0.276875 | 0.006506 | 0.294353 | 0.006575 | 3.95E-01 | 6.42E-02 |
| TG(19:1_18:2_18:2) | 0.113051 | 0.006459 | 0.132894 | 0.006527 | 4.52E-01 | 3.48E-02 |
| TG(20:0_18:1_18:1) | 0.563839 | 0.020484 | 0.70851 | 0.0207 | 1.04E+00 | 3.73E-06 |
| TG(20:0e_16:0_18:0) | 0.02301 | 0.001389 | 0.028137 | 0.001404 | 5.44E-01 | 1.17E-02 |
| TG(20:0e_16:0_18:1) | 0.046724 | 0.002743 | 0.060106 | 0.002772 | 7.18E-01 | 9.94E-04 |
| TG(20:0e_18:1_18:1) | 0.048538 | 0.002718 | 0.064227 | 0.002747 | 8.50E-01 | 1.17E-04 |
| TG(20:0e_18:1_18:2) | 0.047943 | 0.002733 | 0.067057 | 0.002761 | 1.03E+00 | 4.51E-06 |
| TG(20:1_18:1_18:1) | 0.520939 | 0.018764 | 0.622566 | 0.018962 | 7.97E-01 | 2.82E-04 |
| TG(20:1_18:1_22:5) | 0.133745 | 0.007122 | 0.139473 | 0.007197 | 1.18E-01 | 5.76E-01 |
| TG(20:1_18:1_22:6) | 0.117839 | 0.004888 | 0.117902 | 0.00494 | 1.81E-03 | 9.93E-01 |
| TG(20:2_18:2_18:2) | 0.500648 | 0.029435 | 0.631694 | 0.029745 | 6.55E-01 | 2.53E-03 |
| TG(20:3_18:2_18:2) | 0.228724 | 0.018272 | 0.237721 | 0.018465 | 7.25E-02 | 7.32E-01 |
| TG(20:5_18:2_18:2) | 0.129147 | 0.00733 | 0.17848 | 0.007407 | 9.91E-01 | 9.39E-06 |
| TG(20:5_18:2_22:6) | 0.036714 | 0.002719 | 0.019262 | 0.002748 | -9.45E-01 | 2.19E-05 |
| TG(22:1_18:2_22:1) | 0.082888 | 0.003706 | 0.089567 | 0.003746 | 2.65E-01 | 2.12E-01 |
| TG(22:4_18:2_18:2) | 0.349435 | 0.01505 | 0.304124 | 0.015209 | -4.43E-01 | 3.85E-02 |
| TG(22:5_17:1_18:2) | 0.049079 | 0.003906 | 0.028171 | 0.003947 | -7.88E-01 | 3.28E-04 |
| TG(24:0_18:2_18:2) | 0.18614 | 0.0076 | 0.20871 | 0.00768 | 4.37E-01 | 4.11E-02 |
| TG(25:0_16:0_16:0) | 0.058887 | 0.002778 | 0.065374 | 0.002808 | 3.44E-01 | 1.07E-01 |
| TG(25:0_16:0_18:1) | 0.079051 | 0.003982 | 0.076799 | 0.004023 | -8.32E-02 | 6.94E-01 |
| TG(25:0_18:0_18:1) | 0.037089 | 0.002007 | 0.033264 | 0.002028 | -2.80E-01 | 1.87E-01 |
| TG(25:0_18:1_18:1) | 0.063178 | 0.003519 | 0.063109 | 0.003556 | -2.93E-03 | 9.89E-01 |
| TG(26:0_16:0_18:0) | 0.034227 | 0.00198 | 0.033939 | 0.002 | -2.16E-02 | 9.19E-01 |
| TG(26:0_18:1_18:1) | 0.075967 | 0.004384 | 0.072996 | 0.00443 | -9.97E-02 | 6.38E-01 |
| TG(26:0_18:1_18:2) | 0.080438 | 0.004114 | 0.07677 | 0.004158 | -1.31E-01 | 5.35E-01 |
| TG(28:0_18:1_18:1) | 0.033612 | 0.001766 | 0.029641 | 0.001784 | -3.31E-01 | 1.20E-01 |
| TG(29:0_16:0_18:1) | 0.025804 | 0.001383 | 0.021274 | 0.001398 | -4.82E-01 | 2.47E-02 |
| TG(29:0_16:0_18:2) | 0.034867 | 0.0017 | 0.029215 | 0.001718 | -4.89E-01 | 2.27E-02 |
| TG(29:0_18:1_18:1) | 0.028807 | 0.001498 | 0.0225 | 0.001513 | -6.20E-01 | 4.19E-03 |
| TG(29:0_18:1_18:2) | 0.028104 | 0.001647 | 0.022989 | 0.001664 | -4.58E-01 | 3.28E-02 |
| TG(29:1) | 0.131246 | 0.008672 | 0.180251 | 0.008763 | 8.32E-01 | 1.59E-04 |
| TG(30:0_18:1_18:1) | 0.023749 | 0.001357 | 0.022212 | 0.001371 | -1.67E-01 | 4.31E-01 |
| TG(33:0) | 0.019907 | 0.001817 | 0.02193 | 0.001836 | 1.64E-01 | 4.39E-01 |
| TG(33:1) | 0.00992 | 0.00098 | 0.01401 | 0.00099 | 6.14E-01 | 4.51E-03 |
| TG(4:0_14:0_16:0) | 0.024675 | 0.001842 | 0.0189 | 0.001861 | -4.61E-01 | 3.13E-02 |
| TG(4:0_14:2_18:1) | 0.041926 | 0.002685 | 0.050459 | 0.002713 | 4.68E-01 | 2.91E-02 |
| TG(4:0_16:0_16:0) | 0.031144 | 0.002673 | 0.02914 | 0.002701 | -1.10E-01 | 6.02E-01 |
| TG(4:0_16:0_18:1) | 0.051388 | 0.005042 | 0.04501 | 0.005095 | -1.86E-01 | 3.80E-01 |
| TG(4:0_16:0_20:4) | 0.01721 | 0.001292 | 0.020187 | 0.001306 | 3.39E-01 | 1.12E-01 |
| TG(51:3) | 0.040822 | 0.002 | 0.044621 | 0.002021 | 2.80E-01 | 1.89E-01 |
| TG(55:4) | 0.16101 | 0.005738 | 0.147643 | 0.005798 | -3.43E-01 | 1.08E-01 |
| TG(58:1e) | 0.02965 | 0.001472 | 0.035828 | 0.001488 | 6.18E-01 | 4.32E-03 |
| TG(58:3e) | 0.051755 | 0.002558 | 0.066199 | 0.002585 | 8.31E-01 | 1.60E-04 |
| TG(6:0_11:1_14:2) | 0.075384 | 0.007264 | 0.101937 | 0.007341 | 5.38E-01 | 1.25E-02 |
| TG(6:0_11:1_18:2) | 0.034866 | 0.004163 | 0.049822 | 0.004207 | 5.29E-01 | 1.40E-02 |
| TG(6:0_11:1_18:3) | 0.077918 | 0.006749 | 0.097618 | 0.00682 | 4.30E-01 | 4.46E-02 |
| TG(60:2e) | 0.025393 | 0.001154 | 0.029273 | 0.001166 | 4.95E-01 | 2.12E-02 |
| TG(60:3e) | 0.038983 | 0.001657 | 0.046714 | 0.001675 | 6.86E-01 | 1.60E-03 |
| TG(60:4e) | 0.038925 | 0.001872 | 0.048228 | 0.001892 | 7.31E-01 | 8.06E-04 |
| TG(8:0_18:1_18:3) | 0.041983 | 0.003497 | 0.046058 | 0.003534 | 1.71E-01 | 4.18E-01 |
| TG(8:0_8:0_10:0) | 0.07456 | 0.008081 | 0.063636 | 0.008166 | -1.99E-01 | 3.48E-01 |
| TG(8:0_8:0_8:0) | 0.047817 | 0.00475 | 0.04426 | 0.0048 | -1.10E-01 | 6.03E-01 |
| TG(9:0_10:0_10:0) | 0.10099 | 0.009004 | 0.133829 | 0.009099 | 5.37E-01 | 1.26E-02 |
