# Supplementary material for: Plasma lipidomic biomarker analysis reveals distinct lipid changes in vascular dementia
Source: Comput Struct Biotechnol J. 2020 Jun 9;18:1613–24. doi: 10.1016/j.csbj.2020.06.001 (PMC7334482; doi:10.1016/j.csbj.2020.06.001)
Supplement: Supplementary data 5 [file mmc5.docx]

Supplementary table 3. Random forest rank and GLM average beta of lipid classifications

| **Lipid Class** | **LipidName** | **Rank** | **AverageBeta** |
| --- | --- | --- | --- |
| GroupLipids | GroupCer | 1 | -2.287871334 |
| GroupLipids | GroupPC | 2 | -0.493071567 |
| GroupLipids | GroupTG | 3 | 0.379349599 |
| GroupLipids | GroupPE | 4 | 0.145985119 |
| GroupLipids | GroupChE | 5 | -0.184520099 |
| GroupLipids | GroupLPC | 6 | -0.004073649 |
| GroupLipids | GroupDG | 7 | -0.094359835 |
| GroupLipids | GroupPI | 8 | -0.301068016 |
| GroupLipids | GroupSM | 9 | 0.654555936 |
| Cer | Cer(d18:2_24:0) | 1 | -1.089945629 |
| Cer | Cer(m18:1_23:0) | 2 | -0.62628509 |
| Cer | Cer(m18:0_23:0) | 3 | -0.136009602 |
| Cer | Cer(m18:1_20:0) | 4 | -0.074740035 |
| Cer | Cer(m18:1_22:0) | 5 | -0.930540193 |
| Cer | Cer(d18:2_25:0) | 6 | -0.118895334 |
| Cer | Cer(m18:0_18:0) | 7 | -0.003142103 |
| Cer | Cer(m18:1_18:0) | 8 | -0.061518856 |
| Cer | Cer(d18:1_22:0) | 9 | -0.114709847 |
| Cer | Cer(d18:2_24:1) | 10 | 0.004690133 |
| Cer | Cer(m18:0_24:0) | 11 | -0.179647884 |
| Cer | Cer(d18:1_18:0) | 12 | -0.002348589 |
| Cer | Cer(t16:1_12:0) | 13 | 0.100903295 |
| Cer | Cer(m18:0_20:0) | 14 | -0.037608163 |
| Cer | Cer(d18:1_20:0) | 15 | -0.044148044 |
| Cer | Cer(d16:1_23:0) | 16 | -0.056095849 |
| Cer | Cer(d18:2_20:0) | 17 | -0.498040629 |
| Cer | Cer(d18:0_18:0) | 18 | 0.016975227 |
| Cer | Cer(d18:1_23:0) | 19 | -0.040683945 |
| Cer | Cer(m18:0_22:0) | 20 | -0.046639206 |
| Cer | Cer(m18:1_24:1) | 21 | 0.102748519 |
| Cer | Cer(d18:1_24:0) | 22 | -0.045242256 |
| Cer | Cer(m18:1_24:0_O) | 23 | -0.046989312 |
| Cer | Cer(t16:1_16:0) | 24 | -0.081750188 |
| Cer | Cer(m18:0_24:1) | 25 | 0.014901145 |
| Cer | Cer(m18:1_24:2) | 26 | 0.052363597 |
| Cer | Cer(d16:1_16:0) | 27 | -0.13762902 |
| Cer | Cer(d16:1_21:2) | 28 | -0.26057568 |
| Cer | Cer(d18:1_25:0) | 29 | -0.044392638 |
| Cer | Cer(d19:1_24:0) | 30 | -0.440757064 |
| Cer | Cer(d18:2_23:0) | 31 | -0.133515463 |
| Cer | Cer(d17:1_20:0) | 32 | -0.05889554 |
| Cer | Cer(d18:1_16:0) | 33 | 0.23916294 |
| Cer | Cer(d18:0_22:0) | 34 | -0.062539496 |
| Cer | Cer(d18:0_24:0) | 35 | -0.014466376 |
| Cer | Cer(d19:1_24:1) | 36 | -0.003612473 |
| Cer | Cer(d18:0_24:1) | 37 | 0.060861151 |
| Cer | Cer(d17:1_16:0) | 38 | -0.175051519 |
| Cer | Cer(d19:2_23:0_O) | 39 | -0.012727188 |
| Cer | Cer(d18:1_24:1) | 40 | 0.018072393 |
| Cer | Cer(d18:0_16:0) | 41 | 0.01419142 |
| Cer | Cer(d18:0_23:0) | 42 | -0.230095119 |
| Cer | Cer(d16:0_24:1) | 43 | <0.000000001 |
| Cer | Cer(t18:0_22:0) | 44 | -0.004290905 |
| Cer | Cer(d18:1_26:1) | 45 | 0.018414748 |
| Cer | Cer(t18:0_23:0) | 46 | -0.00102772 |
| Cer | Cer(d18:2_22:0) | 47 | 0.090515952 |
| Cer | Cer(t18:0_24:1) | 48 | 0.059951063 |
| Cer | Cer(t18:0_24:0) | 49 | -0.026809863 |
| ChE | ChE(20:5) | 1 | -1.001548973 |
| ChE | ChE(16:0) | 2 | -0.666928127 |
| ChE | ChE(18:1) | 3 | -0.213197609 |
| ChE | ChE(22:6) | 4 | -1.254665201 |
| ChE | ChE(20:4) | 5 | -1.03910177 |
| ChE | ChE(16:1) | 6 | -0.038952462 |
| ChE | ChE(17:1) | 7 | -0.108234056 |
| ChE | ChE(18:0) | 8 | -1.952151386 |
| ChE | ChE(18:3) | 9 | 0.914833026 |
| ChE | ChE(18:2) | 10 | 1.43332399 |
| ChE | ChE(17:0) | 11 | 0.24639872 |
| ChE | ChE(20:3) | 12 | -1.9998118 |
| ChE | ChE(14:0) | 13 | 0.126677982 |
| ChE | ChE(22:5) | 14 | 1.377758174 |
| ChE | ChE(20:2) | 15 | -0.230926539 |
| ChE | ChE(22:4) | 16 | 0.152609134 |
| ChE_Cer | Cer(m18:1_23:0) | 1 | -0.374823239 |
| ChE_Cer | Cer(d18:2_24:0) | 2 | -1.132107353 |
| ChE_Cer | ChE(18:2) | 3 | -0.347557755 |
| ChE_Cer | Cer(d18:2_25:0) | 4 | -0.201168608 |
| ChE_Cer | Cer(m18:0_23:0) | 5 | -1.193057374 |
| ChE_Cer | ChE(20:3) | 6 | -0.529137422 |
| ChE_Cer | ChE(18:1) | 7 | -0.174688571 |
| ChE_Cer | ChE(20:5) | 8 | -0.144373088 |
| ChE_Cer | ChE(18:0) | 9 | -0.414221946 |
| ChE_Cer | Cer(m18:1_20:0) | 10 | -0.119949955 |
| ChE_Cer | ChE(16:0) | 11 | -0.473528191 |
| ChE_Cer | Cer(d18:1_25:0) | 12 | -0.036709698 |
| ChE_Cer | Cer(d18:2_20:0) | 13 | -0.3627301 |
| ChE_Cer | ChE(20:4) | 14 | -0.523083308 |
| ChE_Cer | ChE(18:3) | 15 | 0.310481013 |
| ChE_Cer | Cer(d18:1_22:0) | 16 | -0.08960639 |
| ChE_Cer | Cer(d19:1_24:1) | 17 | 0.048599102 |
| ChE_Cer | Cer(m18:1_24:0_O) | 18 | -0.261202654 |
| ChE_Cer | ChE(22:4) | 19 | 0.026385666 |
| ChE_Cer | Cer(m18:1_22:0) | 20 | -0.266430936 |
| ChE_Cer | ChE(17:1) | 21 | -0.393097707 |
| ChE_Cer | Cer(d18:1_23:0) | 22 | -0.072909149 |
| ChE_Cer | Cer(t16:1_12:0) | 23 | 0.512057131 |
| ChE_Cer | ChE(17:0) | 24 | -0.053714395 |
| ChE_Cer | Cer(d18:1_26:1) | 25 | 0.040909802 |
| ChE_Cer | Cer(d18:0_23:0) | 26 | -0.017211894 |
| ChE_Cer | ChE(20:2) | 27 | -0.214734896 |
| ChE_Cer | Cer(d18:1_20:0) | 28 | -0.109448681 |
| ChE_Cer | ChE(14:0) | 29 | -0.069178264 |
| ChE_Cer | ChE(22:6) | 30 | -0.75564082 |
| ChE_Cer | Cer(d17:1_16:0) | 31 | -0.249147878 |
| ChE_Cer | Cer(d17:1_20:0) | 32 | -0.038611889 |
| ChE_Cer | Cer(m18:1_18:0) | 33 | -0.282903391 |
| ChE_Cer | Cer(m18:0_22:0) | 34 | -0.205660247 |
| ChE_Cer | Cer(m18:1_24:2) | 35 | 0.003405092 |
| ChE_Cer | Cer(m18:1_24:1) | 36 | 0.135941483 |
| ChE_Cer | Cer(d16:1_23:0) | 37 | -0.164300461 |
| ChE_Cer | Cer(d16:1_16:0) | 38 | -0.205286932 |
| ChE_Cer | Cer(m18:0_24:0) | 39 | -0.961276669 |
| ChE_Cer | Cer(m18:0_20:0) | 40 | -0.277419883 |
| ChE_Cer | Cer(m18:0_24:1) | 41 | -0.049621345 |
| ChE_Cer | Cer(t16:1_16:0) | 42 | -0.401619283 |
| ChE_Cer | Cer(t18:0_24:1) | 43 | 0.334339417 |
| ChE_Cer | Cer(t18:0_23:0) | 44 | 0.096320018 |
| ChE_Cer | Cer(d18:0_16:0) | 45 | 0.188676593 |
| ChE_Cer | Cer(d18:1_16:0) | 46 | 0.283083105 |
| ChE_Cer | Cer(d18:2_24:1) | 47 | -0.009543512 |
| ChE_Cer | ChE(22:5) | 48 | 0.60505718 |
| ChE_Cer | Cer(d18:2_23:0) | 49 | 0.075574021 |
| ChE_Cer | Cer(d18:0_22:0) | 50 | -0.067931128 |
| ChE_Cer | Cer(d16:1_21:2) | 51 | -0.482185263 |
| ChE_Cer | Cer(d19:1_24:0) | 52 | -0.118381516 |
| ChE_Cer | Cer(d18:0_24:0) | 53 | -0.078680716 |
| ChE_Cer | Cer(d18:1_24:0) | 54 | -0.131229452 |
| ChE_Cer | Cer(d18:1_18:0) | 55 | -0.011419087 |
| ChE_Cer | Cer(d18:0_18:0) | 56 | 0.053452967 |
| ChE_Cer | Cer(t18:0_22:0) | 57 | 0.015898553 |
| ChE_Cer | Cer(d18:1_24:1) | 58 | 0.035783461 |
| ChE_Cer | Cer(d16:0_24:1) | 59 | -0.016998745 |
| ChE_Cer | Cer(d18:2_22:0) | 60 | 0.045285667 |
| ChE_Cer | Cer(d18:0_24:1) | 61 | 0.630386389 |
| ChE_Cer | ChE(16:1) | 62 | 0.049675126 |
| ChE_Cer | Cer(t18:0_24:0) | 63 | -0.024574349 |
| ChE_Cer | Cer(d19:2_23:0_O) | 64 | 0.076116239 |
| ChE_Cer | Cer(m18:0_18:0) | 65 | -0.185859763 |
| DG | DG(12:0_20:5) | 1 | 0.102925484 |
| DG | DG(18:0_18:0) | 2 | 0.526791459 |
| DG | DG(20:5_18:2) | 3 | -0.016583837 |
| DG | DG(14:0_20:4) | 4 | -0.04562419 |
| DG | DG(16:0_18:2) | 5 | 0.141219471 |
| DG | DG(18:1_22:6) | 6 | -0.298010784 |
| DG | DG(29:3) | 7 | 0.129451026 |
| DG | DG(18:3_18:2) | 8 | 0.133358527 |
| DG | DG(18:0_16:0) | 9 | 0.014577599 |
| DG | DG(16:0_18:1) | 10 | 0.547450288 |
| DG | DG(33:4) | 11 | 0.023862214 |
| DG | DG(18:2_20:4) | 12 | 0.016260249 |
| DG | DG(29:4) | 13 | 0.002928086 |
| DG | DG(16:0_20:5) | 14 | -0.109684349 |
| DG | DG(18:1_18:1) | 15 | -0.011122342 |
| DG | DG(18:1_22:5) | 16 | -0.416976425 |
| DG | DG(20:1_18:2) | 17 | 0 |
| DG | DG(18:2_18:2) | 18 | 0.05990681 |
| DG | DG(22:5_18:2) | 19 | -0.049392989 |
| DG | DG(16:0_16:0) | 20 | 0.009445709 |
| DG | DG(18:1_18:2) | 21 | -0.001566137 |
| DG | DG(14:0_18:3) | 22 | 0.000929836 |
| DG | DG(20:0_18:2) | 23 | 0.064020587 |
| DG | DG(14:0_18:2) | 24 | -0.017329978 |
| DG | DG(17:1_18:1) | 25 | 0.093438171 |
| DG | DG(17:0_18:1) | 26 | -0.15785839 |
| DG | DG(22:3e) | 27 | 0.055555363 |
| DG | DG(16:1_18:3) | 28 | 0.003656767 |
| DG | DG(18:1_20:3) | 29 | 0.002788485 |
| DG | DG(18:1_22:4) | 30 | 0.006486311 |
| DG | DG(17:1_18:2) | 31 | 0.017455031 |
| DG | DG(20:0_18:1) | 32 | 2.07E-05 |
| DG | DG(18:1_14:0) | 33 | -0.013645135 |
| DG | DG(18:0_18:1) | 34 | 0.024557332 |
| DG | DG(16:1_18:2) | 35 | 0.023144626 |
| DG | DG(20:3_18:2) | 36 | -0.012796228 |
| DG | DG(18:1_18:3) | 37 | 0.003353326 |
| DG | DG(18:1_20:4) | 38 | 0.022629443 |
| DG | DG(20:1_18:1) | 39 | -0.036807826 |
| DG | DG(16:0_22:6) | 40 | -0.10726008 |
| DG | DG(15:0_18:2) | 41 | -0.024502448 |
| DG | DG(16:0_18:3) | 42 | 0.016653242 |
| DG | DG(18:2_22:6) | 43 | 0.002835755 |
| LPC | LPC(24:0) | 1 | -1.829322158 |
| LPC | LPC(15:0) | 2 | -0.183154621 |
| LPC | LPC(14:0) | 3 | -1.928891674 |
| LPC | LPC(17:0) | 4 | -0.528321899 |
| LPC | LPC(17:1) | 5 | 0.084595917 |
| LPC | LPC(22:6) | 6 | 0.119478551 |
| LPC | LPC(22:4) | 7 | -0.071910984 |
| LPC | LPC(20:2) | 8 | 0.183947278 |
| LPC | LPC(18:0e) | 9 | 0.49935022 |
| LPC | LPC(26:0) | 10 | -0.570750198 |
| LPC | LPC(22:1) | 11 | -0.601149524 |
| LPC | LPC(16:0) | 12 | 0.120734645 |
| LPC | LPC(16:0e) | 13 | 1.337568995 |
| LPC | LPC(18:3) | 14 | -0.247555322 |
| LPC | LPC(24:1) | 15 | -0.407668415 |
| LPC | LPC(18:0) | 16 | 0.05425985 |
| LPC | LPC(20:1) | 17 | 0.113055836 |
| LPC | LPC(22:5) | 18 | 0.174062696 |
| LPC | LPC(20:4) | 19 | 0.044851011 |
| LPC | LPC(16:1) | 20 | 0.447390963 |
| LPC | LPC(20:5) | 21 | 0.000213261 |
| LPC | LPC(22:0) | 22 | 0.169136831 |
| LPC | LPC(18:2) | 23 | 0.15863827 |
| LPC | LPC(18:1) | 24 | 0.18544999 |
| LPC | LPC(20:3) | 25 | 0.217332513 |
| PC | PC(36:6) | 1 | -0.086159661 |
| PC | PC(40:9) | 2 | -0.08923806 |
| PC | PC(40:7) | 3 | -0.683239264 |
| PC | PC(38:8) | 4 | -0.025940326 |
| PC | PC(37:6) | 5 | -0.049128766 |
| PC | PC(22:6_13:0) | 6 | -0.069500873 |
| PC | PC(40:8) | 7 | -0.14123781 |
| PC | PC(40:6) | 8 | -0.116998208 |
| PC | PC(42:7) | 9 | -0.131078445 |
| PC | PC(42:10) | 10 | -0.146225079 |
| PC | PC(39:6) | 11 | -0.014842581 |
| PC | PC(15:0_20:4) | 12 | -0.0305863 |
| PC | PC(37:1) | 13 | -0.061258841 |
| PC | PC(38:3) | 14 | -0.108876476 |
| PC | PC(34:5) | 15 | -0.038688622 |
| PC | PC(36:5) | 16 | -0.007538145 |
| PC | PC(38:6) | 17 | -0.116615113 |
| PC | PC(33:5) | 18 | -0.005883443 |
| PC | PC(39:3) | 19 | -0.075800851 |
| PC | PC(40:3) | 20 | 0.377447034 |
| PC | PC(41:6) | 21 | 0.002383551 |
| PC | PC(29:0) | 22 | -0.118106988 |
| PC | PC(30:1) | 23 | -0.271013684 |
| PC | PC(28:0) | 24 | -0.133953932 |
| PC | PC(38:1) | 25 | -0.071407791 |
| PC | PC(42:9) | 26 | -0.051588414 |
| PC | PC(39:4) | 27 | 0.021140207 |
| PC | PC(40:1) | 28 | 0.012231076 |
| PC | PC(36:1e) | 29 | -0.002440312 |
| PC | PC(32:1e) | 30 | 0.036222792 |
| PC | PC(42:4) | 31 | 0.080058461 |
| PC | PC(37:5) | 32 | -0.017043819 |
| PC | PC(42:6) | 33 | 0.050631781 |
| PC | PC(41:7) | 34 | -0.152263973 |
| PC | PC(31:0) | 35 | -0.016479408 |
| PC | PC(38:5) | 36 | -0.016539664 |
| PC | PC(36:4) | 37 | 0.076018655 |
| PC | PC(34:2e) | 38 | 0.005121015 |
| PC | PC(44:5) | 39 | 0.010349266 |
| PC | PC(42:2) | 40 | -0.088869232 |
| PC | PC(42:2e) | 41 | -0.380880476 |
| PC | PC(38:6e) | 42 | -0.004544277 |
| PC | PC(36:1) | 43 | 0.020217964 |
| PC | PC(35:2) | 44 | -0.048926628 |
| PC | PC(35:0) | 45 | -0.016675966 |
| PC | PC(36:4e) | 46 | 0.15194361 |
| PC | PC(36:2e) | 47 | -0.00128685 |
| PC | PC(42:5) | 48 | -0.021925459 |
| PC | PC(32:1) | 49 | -0.022084664 |
| PC | PC(38:4) | 50 | 0.007500744 |
| PC | PC(16:1_22:5) | 51 | -0.076253772 |
| PC | PC(35:1) | 52 | -0.007068742 |
| PC | PC(33:1) | 53 | -0.012285007 |
| PC | PC(33:2) | 54 | 0.023562801 |
| PC | PC(18:2_18:2) | 55 | 0.007435984 |
| PC | PC(40:2e) | 56 | 0.054837217 |
| PC | PC(37:3) | 57 | -0.035301124 |
| PC | PC(31:0e) | 58 | -0.000568465 |
| PC | PC(34:1e) | 59 | -0.026647722 |
| PC | PC(35:5) | 60 | 0.013180318 |
| PC | PC(40:5e) | 61 | 0.022287874 |
| PC | PC(38:4e) | 62 | 0.022650363 |
| PC | PC(44:11) | 63 | -0.031637652 |
| PC | PC(32:2) | 64 | -0.038316299 |
| PC | PC(37:4) | 65 | 0.055090384 |
| PC | PC(40:5) | 66 | 0.004124921 |
| PC | PC(18:1_18:1) | 67 | 0.002043396 |
| PC | PC(40:6e) | 68 | 0.078709528 |
| PC | PC(34:0) | 69 | -0.021429824 |
| PC | PC(34:0e) | 70 | -0.007404302 |
| PC | PC(39:7) | 71 | -0.002605753 |
| PC | PC(33:0) | 72 | -0.039758817 |
| PC | PC(30:0) | 73 | -0.023812673 |
| PC | PC(40:4) | 74 | 0.04447077 |
| PC | PC(26:0) | 75 | 0.1383933 |
| PC | PC(38:2) | 76 | 0.01188878 |
| PC | PC(30:2) | 77 | -0.001526112 |
| PC | PC(18:1_13:0) | 78 | -0.003024919 |
| PC | PC(36:0) | 79 | -0.042356327 |
| PC | PC(40:2) | 80 | -0.048176224 |
| PC | PC(33:0e) | 81 | 0.030987164 |
| PC | PC(44:10) | 82 | 0.15919913 |
| PC | PC(32:0e) | 83 | 0.000793476 |
| PC | PC(16:1_18:1) | 84 | -0.137726753 |
| PC | PC(36:3) | 85 | 0.009839861 |
| PC | PC(15:0_18:2) | 86 | -0.025918317 |
| PC | PC(30:0e) | 87 | -0.022847105 |
| PC | PC(39:5) | 88 | -0.037775737 |
| PC | PC(31:1) | 89 | -0.012114824 |
| PC | PC(42:8) | 90 | 0.00106738 |
| PC | PC(18:2_20:4) | 91 | -0.001829174 |
| PC | PC(33:4) | 92 | 0.207094948 |
| PC | PC(42:6e) | 93 | 0.051464206 |
| PC | PC(37:2) | 94 | -0.000370442 |
| PC | PC(32:0) | 95 | 0.017399156 |
| PC | PC(30:1e) | 96 | -0.058509322 |
| PC | PC(35:3) | 97 | -0.021710566 |
| PC | PC(18:2_13:0) | 98 | -0.03555756 |
| PC | PC(39:2) | 99 | 0.356323844 |
| PC | PC(36:0e) | 100 | 0.035396411 |
| PC | PC(20:2_18:2) | 101 | 0.00034912 |
| PC | PC(36:2) | 102 | 0.000686293 |
| PC | PC(34:4) | 103 | -0.051590931 |
| PC | PC(28:1) | 104 | -0.004617142 |
| PC | PC(41:5) | 105 | 0.083813143 |
| PC | PC(21:3e) | 106 | 0.156844219 |
| PC | PC(35:6) | 107 | 0.032746655 |
| PC | PC(44:12) | 108 | 0.052688807 |
| PC | PC(38:2e) | 109 | -0.073761192 |
| PC | PC(32:3) | 110 | 0.000677718 |
| PC | PC(33:3) | 111 | 0 |
| PC | PC(34:1) | 112 | 0.015117312 |
| PC | PC(34:2) | 113 | -0.039790236 |
| PC | PC(42:3) | 114 | 0.111007065 |
| PC | PC(34:3) | 115 | 0.132495371 |
| PE | PE(18:0_22:6) | 1 | -0.181105905 |
| PE | PE(16:0_22:6) | 2 | 0.012785465 |
| PE | PE(16:0_20:5) | 3 | -0.106394061 |
| PE | PE(18:1_22:6) | 4 | -0.426539825 |
| PE | PE(18:0p_22:6) | 5 | -0.553044694 |
| PE | PE(18:0_18:1) | 6 | -0.172682503 |
| PE | PE(16:0p_22:6) | 7 | 0.13819066 |
| PE | PE(16:0p_20:5) | 8 | 0.006400567 |
| PE | PE(18:1_18:2) | 9 | 0.254879939 |
| PE | PE(18:0_20:4) | 10 | -0.101600298 |
| PE | PE(18:0p_20:5) | 11 | 0.618321655 |
| PE | PE(18:0p_22:5) | 12 | 0.006614042 |
| PE | PE(18:0p_22:4) | 13 | 0.105464683 |
| PE | PE(18:1_20:4) | 14 | -0.399918993 |
| PE | PE(10:0e_10:4) | 15 | -0.13429983 |
| PE | PE(16:0p_16:1) | 16 | -0.326181333 |
| PE | PE(18:0p_18:2) | 17 | 0.070265496 |
| PE | PE(18:1p_22:5) | 18 | -0.345581889 |
| PE | PE(18:2p_18:2) | 19 | -0.208153785 |
| PE | PE(16:0p_16:0) | 20 | -0.006998083 |
| PE | PE(16:0_18:2) | 21 | 0.027697064 |
| PE | PE(16:0p_18:1) | 22 | -0.029282965 |
| PE | PE(20:3e) | 23 | -0.124902023 |
| PE | PE(18:1p_22:6) | 24 | -0.830481149 |
| PE | PE(16:0p_18:2) | 25 | 0.136953365 |
| PE | PE(16:0_20:4) | 26 | 0.129790828 |
| PE | PE(18:2e) | 27 | 0.075349668 |
| PE | PE(20:1p_22:6) | 28 | 0.428794724 |
| PE | PE(16:0p_20:4) | 29 | -0.297102598 |
| PE | PE(18:1p_18:2) | 30 | -0.198617365 |
| PE | PE(16:0p_22:5) | 31 | 0.174023987 |
| PE | PE(18:0e) | 32 | -0.042954803 |
| PE | PE(16:0_18:3) | 33 | -0.132293764 |
| PE | PE(18:0p_20:4) | 34 | 0.140212894 |
| PE | PE(16:0_18:1) | 35 | 0.065842256 |
| PE | PE(20:0p_22:6) | 36 | 0.1249126 |
| PE | PE(18:0p_20:3) | 37 | -0.02180141 |
| PE | PE(20:0p_22:5) | 38 | -0.038054966 |
| PI | PI(18:0_22:6) | 1 | -1.018679994 |
| PI | PI(18:0_18:2) | 2 | 0.757993031 |
| PI | PI(18:0_20:4) | 3 | -0.616933443 |
| PI | PI(36:3) | 4 | -0.659205096 |
| PI | PI(18:0_20:3) | 5 | -0.817925041 |
| PI | PI(18:0_22:5) | 6 | 0.096123105 |
| PI | PI(18:1_18:2) | 7 | 0.230645964 |
| PI | PI(16:0_18:2) | 8 | 0.408265325 |
| PI | PI(16:0_22:6) | 9 | 0.126056932 |
| PI | PI(18:1_20:4) | 10 | -0.179675889 |
| PI | PI(35:2) | 11 | -0.813737689 |
| PI | PI(32:1) | 12 | -0.485675904 |
| PI | PI(16:0_20:4) | 13 | 0.214365211 |
| PI | PI(18:0_18:1) | 14 | -0.18244053 |
| PI | PI(34:1) | 15 | 0.291279682 |
| PI | PI(16:0_18:1) | 16 | 0.446026749 |
| SM | SM_d42:5) | 1 | 0.966963615 |
| SM | SM_d18:1_18:3) | 2 | -0.000550681 |
| SM | SM_t42:2) | 3 | 0.258713543 |
| SM | SM_d42:3) | 4 | -0.094222781 |
| SM | SM_d37:2) | 5 | -0.172902185 |
| SM | SM_d39:1) | 6 | -0.101413887 |
| SM | SM_d37:1) | 7 | 0.367747934 |
| SM | SM_d31:1) | 8 | -0.062574336 |
| SM | SM_d18:1_21:0) | 9 | -0.202342927 |
| SM | SM_d43:3) | 10 | -0.013758559 |
| SM | SM_d38:1) | 11 | -0.850075812 |
| SM | SM_t18:0_23:1) | 12 | 0.747098381 |
| SM | SM_d44:1) | 13 | -0.097883108 |
| SM | SM_t34:2) | 14 | 0.0401437 |
| SM | SM_d34:5) | 15 | -0.433595806 |
| SM | SM_d33:2) | 16 | 0.111236033 |
| SM | SM_d43:2) | 17 | -0.19495214 |
| SM | SM_d18:1_21:1) | 18 | -0.150965774 |
| SM | SM_d44:4) | 19 | -0.187626075 |
| SM | SM_d35:2) | 20 | -0.098204122 |
| SM | SM_d40:3) | 21 | -0.457187459 |
| SM | SM_t42:4) | 22 | -0.009405384 |
| SM | SM_d42:4) | 23 | 0.182611499 |
| SM | SM_d36:0) | 24 | 0.075812525 |
| SM | SM_d34:4) | 25 | -0.213048333 |
| SM | SM_d18:2_24:3) | 26 | -0.372188927 |
| SM | SM_d42:2) | 27 | -0.092526727 |
| SM | SM_d42:1) | 28 | 0.024416022 |
| SM | SM_d18:1_13:0) | 29 | -0.090685901 |
| SM | SM_d32:2) | 30 | -0.211321061 |
| SM | SM_d38:2) | 31 | -0.193397918 |
| SM | SM_d43:1) | 32 | -0.018192622 |
| SM | SM_t32:1) | 33 | 0.060966384 |
| SM | SM_t40:7) | 34 | -0.64743245 |
| SM | SM_t36:1) | 35 | 0.053796018 |
| SM | SM_d40:1) | 36 | -0.04635364 |
| SM | SM_d33:1) | 37 | 0.035411906 |
| SM | SM_d35:4) | 38 | 0.224979841 |
| SM | SM_d28:1) | 39 | -0.118667228 |
| SM | SM_d38:0) | 40 | -0.066495033 |
| SM | SM_t34:0) | 41 | -0.076047629 |
| SM | SM_d39:2) | 42 | 0.026539674 |
| SM | SM_t42:3) | 43 | 0.08163629 |
| SM | SM_d44:6) | 44 | 0.416350988 |
| SM | SM_d36:1) | 45 | -0.528952424 |
| SM | SM_d41:3) | 46 | 0.075060142 |
| SM | SM_d41:2) | 47 | 0.055310633 |
| SM | SM_t40:0) | 48 | -0.001911241 |
| SM | SM_d41:0) | 49 | 0.100470012 |
| SM | SM_d35:0) | 50 | 0.013520669 |
| SM | SM_d32:1) | 51 | -0.221060829 |
| SM | SM_t34:1) | 52 | 0.042589492 |
| SM | SM_d43:4) | 53 | -0.136873054 |
| SM | SM_d36:3) | 54 | -0.156800348 |
| SM | SM_d41:1) | 55 | -0.275476773 |
| SM | SM_d44:2) | 56 | 0.111966358 |
| SM | SM_d44:5) | 57 | 0.083206186 |
| SM | SM_d42:6) | 58 | -0.1511341 |
| SM | SM_d34:3) | 59 | -0.186364153 |
| SM | SM_d40:2) | 60 | 0.132097895 |
| SM | SM_t39:5) | 61 | 0.078978895 |
| SM | SM_d38:3) | 62 | 0.089989183 |
| SM | SM_d17:1_13:0) | 63 | 0.661977862 |
| SM | SM_d35:1) | 64 | -0.195355688 |
| SM | SM_t18:0_16:1) | 65 | -0.011115615 |
| SM | SM_t40:1) | 66 | 0.644939428 |
| SM | SM_d16:1_20:0) | 67 | 0.225706287 |
| SM | SM_d44:3) | 68 | 0.030256913 |
| SM | SM_d36:2) | 69 | -0.107831168 |
| SM | SM_t18:0_24:2) | 70 | 0.35512775 |
| SM | SM_d34:2) | 71 | -0.0546292 |
| SM | SM_t42:1) | 72 | 0.067190174 |
| SM | SM_d32:0) | 73 | -0.312837615 |
| SM | SM_d34:0) | 74 | 0.104072688 |
| SM | SM_d34:1) | 75 | 0.168970439 |
| SM | SM_d32:4) | 76 | 0.561710245 |
| SM | SM_t36:2) | 77 | -0.0274125 |
| SM | SM_d40:4) | 78 | 0.09274373 |
| SM | SM_d30:1) | 79 | 3.66E-05 |
| TG | TG(12:0_18:2_22:6) | 1 | -0.031821899 |
| TG | TG(15:0_14:0_22:6) | 2 | -0.034206254 |
| TG | TG(18:0_18:1_20:4) | 3 | 0.126903184 |
| TG | TG(15:0_18:2_22:6) | 4 | -0.071283153 |
| TG | TG(18:4_16:0_18:1) | 5 | 0.0278822 |
| TG | TG(15:0_16:1_18:3) | 6 | -0.121524604 |
| TG | TG(16:0_16:0_18:1) | 7 | 0.012402807 |
| TG | TG(18:1_18:2_22:6) | 8 | 0.033708291 |
| TG | TG(16:0_22:6_22:6) | 9 | -0.117209611 |
| TG | TG(18:1_20:4_22:6) | 10 | -0.057244634 |
| TG | TG(18:3_18:2_18:3) | 11 | -0.010092081 |
| TG | TG(18:1_18:2_20:4) | 12 | 0.043889688 |
| TG | TG(16:0_18:1_23:0) | 13 | 0.055528448 |
| TG | TG(24:0_18:2_18:2) | 14 | 0.029908623 |
| TG | TG(16:0_18:2_20:5) | 15 | 0.013771565 |
| TG | TG(16:0_16:1_20:5) | 16 | 0.056804064 |
| TG | TG(18:1_18:3_20:4) | 17 | 0.026475005 |
| TG | TG(16:0_16:1_18:1) | 18 | 0.010115254 |
| TG | TG(22:1_18:2_22:1) | 19 | 0.01415138 |
| TG | TG(14:0_14:0_22:6) | 20 | -0.004293846 |
| TG | TG(17:0_18:1_18:3) | 21 | -0.002190104 |
| TG | TG(16:0_12:1_18:1) | 22 | -0.000201978 |
| TG | TG(15:0_14:0_20:5) | 23 | -0.052863354 |
| TG | TG(18:3_14:1_18:2) | 24 | 0 |
| TG | TG(18:1_18:1_20:4) | 25 | 0.031395823 |
| TG | TG(18:2_18:2_20:4) | 26 | -0.016577372 |
| TG | TG(12:0_14:0_18:3) | 27 | -0.015072154 |
| TG | TG(15:0_12:0_16:0) | 28 | -0.043324003 |
| TG | TG(19:1_16:0_18:1) | 29 | -0.01508641 |
| TG | TG(18:1_14:1_18:1) | 30 | 0.011345105 |
| TG | TG(14:0_18:3_20:5) | 31 | 0.007812056 |
| TG | TG(8:0_18:1_18:3) | 32 | 0.019124464 |
| TG | TG(16:0_18:1_20:5) | 33 | 0.027223568 |
| TG | TG(6:0_11:1_18:3) | 34 | -0.031976658 |
| TG | TG(16:0_14:0_18:1) | 35 | 0 |
| TG | TG(18:1_20:3_22:6) | 36 | 0.004798354 |
| TG | TG(20:1_18:1_18:1) | 37 | 0.05240072 |
| TG | TG(18:1_18:1_22:5) | 38 | 0.002729172 |
| TG | TG(18:1_12:0_14:3) | 39 | -0.011283304 |
| TG | TG(18:3_18:2_22:6) | 40 | 0.008886836 |
| TG | TG(20:5_18:2_22:6) | 41 | 0.009919589 |
| TG | TG(19:1_18:1_18:2) | 42 | 0.027129291 |
| TG | TG(18:1_18:1_20:2) | 43 | -0.007931958 |
| TG | TG(16:0_14:0_20:5) | 44 | -0.033147271 |
| TG | TG(33:0) | 45 | -0.044956387 |
| TG | TG(22:5_17:1_18:2) | 46 | -0.077511829 |
| TG | TG(16:0_12:0_14:0) | 47 | -0.043518185 |
| TG | TG(6:0_11:1_14:2) | 48 | 0.028970038 |
| TG | TG(60:3e) | 49 | -0.000496326 |
| TG | TG(9:0_10:0_10:0) | 50 | 0.012095439 |
| TG | TG(8:0_8:0_10:0) | 51 | -0.027886234 |
| TG | TG(16:0e_16:0_16:0) | 52 | 0 |
| TG | TG(15:0_16:0_18:2) | 53 | -0.016702647 |
| TG | TG(15:0_16:1_18:2) | 54 | -0.003622163 |
| TG | TG(15:0_18:1_22:6) | 55 | -0.042559375 |
| TG | TG(6:0_11:1_18:2) | 56 | 0.02242135 |
| TG | TG(60:4e) | 57 | 0.008064385 |
| TG | TG(17:0_20:5_22:6) | 58 | 0 |
| TG | TG(16:0_17:1_18:2) | 59 | -0.064814206 |
| TG | TG(18:2_17:1_18:2) | 60 | 0.006828928 |
| TG | TG(16:0_18:3_20:5) | 61 | 0.001101134 |
| TG | TG(16:0_18:3_18:3) | 62 | 0 |
| TG | TG(18:1_18:2_20:3) | 63 | 0 |
| TG | TG(16:1_18:1_18:1) | 64 | 0.022463305 |
| TG | TG(14:0_18:3_22:6) | 65 | -0.023119031 |
| TG | TG(16:0_18:1_19:0) | 66 | -0.00455346 |
| TG | TG(33:1) | 67 | 0.000996423 |
| TG | TG(18:1_13:0_18:1) | 68 | -0.023210393 |
| TG | TG(18:0_18:1_20:0) | 69 | 0 |
| TG | TG(18:1_22:5_22:6) | 70 | 0 |
| TG | TG(20:1_18:1_22:5) | 71 | -0.033460955 |
| TG | TG(18:0_16:0_18:1) | 72 | 0.010880346 |
| TG | TG(18:1_18:2_18:3) | 73 | 0.00103138 |
| TG | TG(29:0_18:0_18:1) | 74 | -0.101994949 |
| TG | TG(16:0_18:2_22:6) | 75 | -0.033802256 |
| TG | TG(15:0_18:1_20:4) | 76 | 0 |
| TG | TG(15:0_12:0_18:3) | 77 | -0.00229625 |
| TG | TG(18:2_22:6_22:6) | 78 | 0.00161451 |
| TG | TG(16:0_14:0_16:1) | 79 | -0.035513675 |
| TG | TG(18:3_18:2_18:2) | 80 | -0.002783435 |
| TG | TG(18:0_20:4_22:5) | 81 | 0.00220728 |
| TG | TG(15:0_16:0_20:4) | 82 | 0.007403082 |
| TG | TG(16:0_11:1_18:1) | 83 | -0.001997759 |
| TG | TG(12:0_12:0_18:2) | 84 | -0.004643483 |
| TG | TG(18:1_18:1_20:3) | 85 | 0.018648792 |
| TG | TG(18:1_14:3_18:2) | 86 | -0.009944124 |
| TG | TG(18:3_18:3_18:3) | 87 | 0.022249975 |
| TG | TG(18:1_20:4_24:0) | 88 | -0.008165358 |
| TG | TG(29:0_16:0_18:2) | 89 | -0.057149856 |
| TG | TG(16:0_16:0_16:0) | 90 | 0.00086955 |
| TG | TG(16:0_10:1_16:0) | 91 | 0.002208327 |
| TG | TG(16:0_18:1_23:1) | 92 | 0.019021038 |
| TG | TG(18:0_17:0_18:1) | 93 | -0.001765009 |
| TG | TG(18:1_12:0_18:2) | 94 | 0.012305766 |
| TG | TG(16:0_16:0_17:0) | 95 | -0.002481186 |
| TG | TG(4:0_16:0_20:4) | 96 | 0.000305432 |
| TG | TG(12:0_18:2_18:3) | 97 | 0.058235097 |
| TG | TG(17:0_18:1_18:1) | 98 | -0.042598745 |
| TG | TG(12:0_14:0_14:0) | 99 | 0 |
| TG | TG(18:2_18:2_18:2) | 100 | 0.027097699 |
| TG | TG(20:5_18:2_18:2) | 101 | 0.032697846 |
| TG | TG(42:6e) | 102 | 0.082937448 |
| TG | TG(12:0_14:0_14:3) | 103 | 0.009030381 |
| TG | TG(15:0_12:0_18:2) | 104 | -0.012909064 |
| TG | TG(15:0_16:0_20:5) | 105 | -0.000765269 |
| TG | TG(18:0e_16:0_16:0) | 106 | 0.00807256 |
| TG | TG(18:1_14:0_22:6) | 107 | 0.007630818 |
| TG | TG(16:0_16:0_18:2) | 108 | -0.000734563 |
| TG | TG(18:1_18:1_18:2) | 109 | 0.002879641 |
| TG | TG(20:0e_16:0_18:1) | 110 | -0.000102349 |
| TG | TG(18:0_17:0_20:4) | 111 | 0.016116817 |
| TG | TG(19:0_18:1_18:1) | 112 | 0.009904131 |
| TG | TG(58:3e) | 113 | 0.006372394 |
| TG | TG(18:0_22:6_22:6) | 114 | 0.000170709 |
| TG | TG(18:1_18:1_22:0) | 115 | 0.015272921 |
| TG | TG(15:0_14:0_18:2) | 116 | -0.033354058 |
| TG | TG(18:0_20:5_22:6) | 117 | -0.017400407 |
| TG | TG(15:0_14:0_18:3) | 118 | -0.065128916 |
| TG | TG(18:1_18:1_24:0) | 119 | 0 |
| TG | TG(20:0e_16:0_18:0) | 120 | -0.00347274 |
| TG | TG(15:0_18:1_18:2) | 121 | -0.003024188 |
| TG | TG(16:0_16:1_18:3) | 122 | -0.000981167 |
| TG | TG(20:0e_18:1_18:2) | 123 | 0.029677869 |
| TG | TG(18:3_17:1_18:2) | 124 | -0.106786477 |
| TG | TG(58:1e) | 125 | -0.009555472 |
| TG | TG(29:1) | 126 | 0 |
| TG | TG(18:2_14:1_18:2) | 127 | -0.00977119 |
| TG | TG(16:0_18:1_20:4) | 128 | 0.005392538 |
| TG | TG(16:0_17:1_18:1) | 129 | -0.000922175 |
| TG | TG(16:0_18:2_18:3) | 130 | 0.002651214 |
| TG | TG(18:3_14:3_18:2) | 131 | 0.004778778 |
| TG | TG(16:1_18:3_18:3) | 132 | 0.000605591 |
| TG | TG(16:0_18:1_18:1) | 133 | 0.033982509 |
| TG | TG(19:1_17:1_17:1) | 134 | -0.009873695 |
| TG | TG(20:1_18:1_22:6) | 135 | 0.020539992 |
| TG | TG(16:0_16:0_24:0) | 136 | 0.000252648 |
| TG | TG(18:1_17:1_20:5) | 137 | 0.021386361 |
| TG | TG(15:0_18:2_18:2) | 138 | -0.023970778 |
| TG | TG(12:0_17:1_18:2) | 139 | -0.000509884 |
| TG | TG(18:0_16:0_17:0) | 140 | -0.000412276 |
| TG | TG(19:1_18:1_18:1) | 141 | 0.008589652 |
| TG | TG(18:1_18:2_20:5) | 142 | 0.025373282 |
| TG | TG(16:0_17:0_18:1) | 143 | -0.00093823 |
| TG | TG(18:1_18:2_18:2) | 144 | 0.000579356 |
| TG | TG(18:1_18:1_22:6) | 145 | 0 |
| TG | TG(20:0e_18:1_18:1) | 146 | 0.000889489 |
| TG | TG(18:0_18:0_18:1) | 147 | 0.022364685 |
| TG | TG(4:0_14:2_18:1) | 148 | 0.007148538 |
| TG | TG(10:0_18:2_18:3) | 149 | 0.034909273 |
| TG | TG(18:1_18:2_23:0) | 150 | 0.00039266 |
| TG | TG(14:0_18:2_20:5) | 151 | 0.007682885 |
| TG | TG(18:1_18:3_18:3) | 152 | 0.00493077 |
| TG | TG(11:0_18:0_18:0) | 153 | -5.63E-05 |
| TG | TG(14:0_18:2_18:2) | 154 | 0 |
| TG | TG(18:0_16:0_17:1) | 155 | -0.004185083 |
| TG | TG(15:0_12:0_18:1) | 156 | -0.067925108 |
| TG | TG(18:1_14:0_18:1) | 157 | -0.11753103 |
| TG | TG(16:0_16:0_18:3) | 158 | 0.008093222 |
| TG | TG(16:0_18:1_22:6) | 159 | -0.013530812 |
| TG | TG(18:0_16:1_17:0) | 160 | 0.004120935 |
| TG | TG(17:0_18:1_22:4) | 161 | -0.001245997 |
| TG | TG(18:1_18:2_21:1) | 162 | 0 |
| TG | TG(12:0_18:2_20:5) | 163 | 0 |
| TG | TG(20:0_18:1_18:1) | 164 | 0.09060886 |
| TG | TG(18:0_16:0_16:0) | 165 | 0.001037537 |
| TG | TG(15:0_16:1_16:1) | 166 | -0.011973255 |
| TG | TG(16:0_18:1_21:0) | 167 | 0 |
| TG | TG(55:4) | 168 | -6.72E-05 |
| TG | TG(16:0_11:1_18:2) | 169 | -0.003766259 |
| TG | TG(29:0_18:1_18:1) | 170 | -0.034239867 |
| TG | TG(12:0_14:0_22:6) | 171 | 0 |
| TG | TG(25:0_18:1_18:1) | 172 | 0.008189873 |
| TG | TG(22:4_18:2_18:2) | 173 | 0.003447684 |
| TG | TG(17:0_18:2_20:4) | 174 | 0.012351391 |
| TG | TG(18:3_18:3_20:5) | 175 | 0.058647199 |
| TG | TG(19:1_18:0_18:1) | 176 | 0 |
| TG | TG(15:0_12:0_12:0) | 177 | -0.015047093 |
| TG | TG(18:1_17:1_18:1) | 178 | -0.060625891 |
| TG | TG(18:1_22:1_23:1) | 179 | 0.024841836 |
| TG | TG(18:1_12:0_14:0) | 180 | -0.000451515 |
| TG | TG(25:0_16:0_16:0) | 181 | 0.022229919 |
| TG | TG(15:0_15:0_15:0) | 182 | 0 |
| TG | TG(12:0_12:0_12:0) | 183 | -0.00686371 |
| TG | TG(18:0_18:1_22:4) | 184 | 0.027250482 |
| TG | TG(18:0_18:1_20:3) | 185 | 0.048825279 |
| TG | TG(16:0_18:1_18:3) | 186 | 0.001726233 |
| TG | TG(16:0_18:1_18:2) | 187 | 2.36E-05 |
| TG | TG(16:0e_16:0_18:1) | 188 | 0.000552985 |
| TG | TG(15:0_18:1_20:5) | 189 | -0.050649205 |
| TG | TG(16:0e_16:0_18:2) | 190 | 0.000362384 |
| TG | TG(15:0_16:0_16:0) | 191 | 0.003015945 |
| TG | TG(16:1_18:2_18:3) | 192 | -0.001322292 |
| TG | TG(15:0_16:0_18:1) | 193 | -0.000793881 |
| TG | TG(16:0_22:1_22:6) | 194 | 0.006149966 |
| TG | TG(26:0_16:0_18:0) | 195 | -0.002767777 |
| TG | TG(20:3_18:2_18:2) | 196 | 0.006961558 |
| TG | TG(18:0_18:0_18:0) | 197 | 0.007889933 |
| TG | TG(26:0_18:1_18:2) | 198 | 0 |
| TG | TG(18:1_18:1_23:1) | 199 | 0 |
| TG | TG(17:0_18:1_22:6) | 200 | -0.005164268 |
| TG | TG(16:0_14:0_16:0) | 201 | 0 |
| TG | TG(18:1_17:1_22:6) | 202 | 0.015679698 |
| TG | TG(16:0_20:4_22:6) | 203 | -0.053413849 |
| TG | TG(16:0_16:1_16:1) | 204 | -0.005473639 |
| TG | TG(18:1_12:0_12:0) | 205 | -0.015127666 |
| TG | TG(10:0_12:0_12:0) | 206 | -0.015107141 |
| TG | TG(15:0_14:0_16:1) | 207 | -0.014568725 |
| TG | TG(19:0_19:0_20:5) | 208 | -0.00739423 |
| TG | TG(18:1_12:0_18:1) | 209 | -0.018929561 |
| TG | TG(8:0_8:0_8:0) | 210 | -0.021868492 |
| TG | TG(18:1_18:1_21:0) | 211 | 0.000713836 |
| TG | TG(18:1_18:1_18:1) | 212 | 0.023002174 |
| TG | TG(18:0_18:1_18:1) | 213 | 0.04048716 |
| TG | TG(18:1_20:4_22:1) | 214 | -0.001797851 |
| TG | TG(18:1_18:3_24:1) | 215 | -0.008075867 |
| TG | TG(18:1_18:1_24:1) | 216 | 0.005213811 |
| TG | TG(18:0e_18:1_18:1) | 217 | -0.003805401 |
| TG | TG(18:0e_16:0_20:4) | 218 | 0.023861142 |
| TG | TG(18:0_17:0_18:0) | 219 | 0.015491162 |
| TG | TG(18:0_10:0_18:0) | 220 | -0.000504192 |
| TG | TG(4:0_16:0_18:1) | 221 | 0 |
| TG | TG(16:1_14:0_18:1) | 222 | 0 |
| TG | TG(4:0_14:0_16:0) | 223 | 0 |
| TG | TG(12:0_12:0_14:0) | 224 | 0 |
| TG | TG(14:0_18:2_18:3) | 225 | 0.001081944 |
| TG | TG(29:0_18:1_18:2) | 226 | -0.014058313 |
| TG | TG(28:0_18:1_18:1) | 227 | -0.020932367 |
| TG | TG(16:0_16:0_23:0) | 228 | 0.001817499 |
| TG | TG(16:0_12:0_18:1) | 229 | -0.010322457 |
| TG | TG(18:1_20:4_22:0) | 230 | 0.016087811 |
| TG | TG(19:1_15:0_17:0) | 231 | 0.001633536 |
| TG | TG(18:0_16:0_24:0) | 232 | 0 |
| TG | TG(18:3_18:2_20:4) | 233 | 0.013617717 |
| TG | TG(60:2e) | 234 | -0.024716302 |
| TG | TG(16:1_18:2_18:2) | 235 | -0.004200049 |
| TG | TG(16:1_18:3_20:5) | 236 | -0.063591425 |
| TG | TG(18:0e_16:0_18:1) | 237 | 0.013854985 |
| TG | TG(20:5_18:2_20:5) | 238 | 0 |
| TG | TG(18:1_12:0_22:6) | 239 | -0.001968424 |
| TG | TG(15:0_18:1_18:3) | 240 | -0.023714632 |
| TG | TG(16:0_12:0_18:3) | 241 | -0.006038493 |
| TG | TG(15:0_18:2_18:3) | 242 | -0.025074567 |
| TG | TG(16:0_14:0_18:3) | 243 | 0 |
| TG | TG(18:1_24:0_24:0) | 244 | -0.028423368 |
| TG | TG(18:0_16:0_18:0) | 245 | 0.015684595 |
| TG | TG(16:0_11:1_16:0) | 246 | -0.005739858 |
| TG | TG(16:0_18:2_18:2) | 247 | 0 |
| TG | TG(18:1_18:1_18:3) | 248 | 0.031557637 |
| TG | TG(25:0_18:0_18:1) | 249 | 0 |
| TG | TG(18:0_18:1_24:0) | 250 | 0 |
| TG | TG(12:0_18:2_18:2) | 251 | -0.002493884 |
| TG | TG(18:2_20:4_22:6) | 252 | 0 |
| TG | TG(16:0_10:0_18:2) | 253 | 0 |
| TG | TG(16:0_14:0_18:2) | 254 | -0.002771041 |
| TG | TG(16:1_16:1_18:1) | 255 | 0.001154278 |
| TG | TG(15:0_16:0_18:3) | 256 | -0.043252138 |
| TG | TG(4:0_16:0_16:0) | 257 | 0 |
| TG | TG(10:0_18:2_18:2) | 258 | 0.095238078 |
| TG | TG(18:3_18:2_20:5) | 259 | -0.000618656 |
| TG | TG(16:0_12:0_20:4) | 260 | 0.000527185 |
| TG | TG(16:0_18:1_24:0) | 261 | 0 |
| TG | TG(18:1_18:1_23:0) | 262 | 0.015170461 |
| TG | TG(16:0_9:0_18:1) | 263 | -0.001123198 |
| TG | TG(18:1_18:1_22:4) | 264 | -0.010320842 |
| TG | TG(19:1_18:2_18:2) | 265 | 0.036135565 |
| TG | TG(18:2_18:2_22:6) | 266 | 0.000990571 |
| TG | TG(18:0_18:1_22:6) | 267 | 0.013543874 |
| TG | TG(10:0_18:1_18:1) | 268 | 0 |
| TG | TG(18:1_17:1_22:1) | 269 | -0.004734682 |
| TG | TG(15:0_14:0_18:1) | 270 | -0.012866472 |
| TG | TG(12:0_18:3_18:3) | 271 | -0.000208401 |
| TG | TG(18:1_17:1_18:2) | 272 | -0.005439541 |
| TG | TG(18:1_18:1_22:1) | 273 | -0.054129044 |
| TG | TG(51:3) | 274 | -0.029391046 |
| TG | TG(15:0_9:0_9:0) | 275 | 0.002301986 |
| TG | TG(16:0_10:1_18:1) | 276 | -0.001647245 |
| TG | TG(16:0_14:0_14:0) | 277 | -0.002247256 |
| TG | TG(16:0_14:1_18:1) | 278 | -0.001852697 |
| TG | TG(16:1_18:2_20:5) | 279 | 0.005963749 |
| TG | TG(18:0_12:1_16:0) | 280 | -0.004055333 |
| TG | TG(20:2_18:2_18:2) | 281 | 0.019963844 |
| TG | TG(25:0_16:0_18:1) | 282 | 0.003928204 |
| TG | TG(26:0_18:1_18:1) | 283 | 0 |
| TG | TG(29:0_16:0_18:1) | 284 | -0.004553897 |
| TG | TG(30:0_18:1_18:1) | 285 | 0.007585477 |
